# Cytosolic Phospholipase A_2_α and Eicosanoids Regulate Expression of Genes in Macrophages Involved in Host Defense and Inflammation

**DOI:** 10.1371/journal.pone.0069002

**Published:** 2013-07-25

**Authors:** Saritha Suram, Lori J. Silveira, Spencer Mahaffey, Gordon D. Brown, Joseph V. Bonventre, David L. Williams, Neil A. R. Gow, Donna L. Bratton, Robert C. Murphy, Christina C. Leslie

**Affiliations:** 1 Department of Pediatrics, National Jewish Health, Denver, Colorado, United States of America; 2 Division of Biostatistics and Bioinformatics, National Jewish Health, Denver, Colorado, United States of America; 3 Institute of Medical Sciences, University of Aberdeen, Aberdeen, United Kingdom; 4 Renal Division, Brigham and Women’s Hospital, Boston, Massachusetts, United States of America; 5 Department of Surgery, James H. Quillen College of Medicine, Johnson City, Tennessee, United States of America; 6 Department of Pharmacology, University of Colorado Denver, Aurora, Colorado, United States of America; 7 Department of Pathology, University of Colorado Denver, Aurora, Colorado, United States of America; Institute of Microbiology, Switzerland

## Abstract

The role of Group IVA cytosolic phospholipase A_2_ (cPLA_2_α) activation in regulating macrophage transcriptional responses to *Candida albicans* infection was investigated. cPLA_2_α releases arachidonic acid for the production of eicosanoids. In mouse resident peritoneal macrophages, prostacyclin, prostaglandin E_2_ and leukotriene C_4_ were produced within minutes of *C. albicans* addition before cyclooxygenase 2 expression. The production of TNFα was lower in *C. albicans*-stimulated cPLA_2_α^+/+^ than cPLA_2_α^-/-^ macrophages due to an autocrine effect of prostaglandins that increased cAMP to a greater extent in cPLA_2_α^+/+^ than cPLA_2_α^-/-^ macrophages. For global insight, differential gene expression in *C. albicans*-stimulated cPLA_2_α^+/+^ and cPLA_2_α^-/-^ macrophages (3 h) was compared by microarray. cPLA_2_α^+/+^ macrophages expressed 86 genes at lower levels and 181 genes at higher levels than cPLA_2_α^-/-^ macrophages (≥2-fold, p<0.05). Several pro-inflammatory genes were expressed at lower levels (*Tnfα*, *Cx3cl1*, *Cd40*, *Ccl5*, *Csf1*, *Edn1*, CxCr7, *Irf1*, *Irf4*, *Akna, Ifnγ*, several IFNγ-inducible GTPases). Genes that dampen inflammation (*Socs3*, *Il10*, *Crem*, *Stat3*, *Thbd*, *Thbs1*, *Abca1*) and genes involved in host defense (*Gja1*, *Csf3*, *Trem1*, *Hdc*) were expressed at higher levels in cPLA_2_α^+/+^ macrophages. Representative genes expressed lower in cPLA_2_α^+/+^ macrophages (*Tnfα, Csf1*) were increased by treatment with a prostacyclin receptor antagonist and protein kinase A inhibitor, whereas genes expressed at higher levels (*Crem*, *Nr4a2*, *Il10*, *Csf3*) were suppressed. The results suggest that *C. albicans* stimulates an autocrine loop in macrophages involving cPLA_2_α, cyclooxygenase 1-derived prostaglandins and increased cAMP that globally effects expression of genes involved in host defense and inflammation.

## Introduction

The oxygenated metabolites of arachidonic acid comprise a large family of bioactive lipids that have diverse roles in regulating homeostatic processes and in modulating inflammation and immune responses [1]. The production of eicosanoids is initiated by the release of arachidonic acid that is metabolized through the 5-lipoxygenase pathway to leukotrienes and by cyclooxygenases (COX) to prostanoids and thromboxane. Eicosanoids are secreted and act locally in an autocrine or paracrine fashion through interaction with specific G-protein coupled receptors (GPCR) to exert their biological effects [2–4]. Leukotrienes are pro-inflammatory mediators but prostaglandins (PG) have pro- and anti-inflammatory effects depending on the cell type-specific GPCR-dependent signal transduction pathways that are triggered [1].

Macrophages are an important source of eicosanoids that are produced rapidly in response to stimulation by bacterial and fungal pathogens [5–8]. Resident tissue macrophages are a first line of defense against invading microorganisms that are recognized by pattern recognition receptors that engage microbial surface structures. We have used resident mouse peritoneal macrophages (RPM) to study the regulation of eicosanoid production in response to the model fungal agonist zymosan, cell wall particles of *Saccharomyces cerevisiae* [9–11]. Zymosan stimulates activation of the Group IVA cytosolic phospholipase A_2_ (cPLA_2_α), the first key regulatory enzyme in RPM that releases arachidonic acid for eicosanoid production [12]. To identify the pattern recognition receptors on RPM that mediate cPLA_2_α activation and eicosanoid production, the more medically relevant fungal pathogen *Candida albicans* was studied [13,14]. We found a role for dectin-1 and -2 that engage β-glucan and mannans on the *C. albicans* cell wall that, together with a MyD88-dependent pathway, promote cPLA_2_α activation and eicosanoid production [13,14]. Although *C. albicans* is a normal commensal organism, it is an opportunistic pathogen that is a leading cause of mycoses particularly in the immunocompromised and critically ill [15]. There has been considerable interest in elucidating the mechanisms regulating immune responses to *C. albicans* because of the prevalence of fungal infections [16].

Eicosanoids affect immune regulation by modulating cellular differentiation, phagocytic potential, migration and cytokine/chemokine production [5,17–19]. The types and balance of cytokines produced during the early responses of innate immune cells to infection influence the macrophage phenotype, differentiation of lymphocytes and adaptive immune responses [20–23]. In this study, we compared cPLA_2_α^+/+^ and cPLA_2_α^-/-^ RPM to investigate the functional consequences of cPLA_2_α activation and the effect of endogenously produced eicosanoids on gene expression in response to *C. albicans*. Our results demonstrate that *C. albicans*-stimulated cPLA_2_α activation and the early production of prostanoids promotes an autocrine pathway in RPM that affects the expression of genes involved in host defense and to dampen inflammation.

## Materials and Methods

### Ethics Statement

The work with mice in this study was approved by the National Jewish Health Institutional Animal Care and Use Committee (IACUC) and conducted in accordance with their guidelines.

### Materials

DMEM was from Cambrex BioScience. FBS (Gemini Bio-Products) was heat inactivated at 56°C for 30 min before use. Human serum albumin was obtained from Intergen. Polyclonal antibodies to murine COX1 and COX2, the protein kinase A inhibitor H-89, the COX inhibitor NS-398, the IP receptor antagonist CAY10441, the IP receptor agonist iloprost and the EP2 receptor agonist butaprost were from Cayman Chemical Co. Antibodies to β-actin were from Cell Signaling. The stable cAMP analogue 8-Br-cAMP was from Santa Cruz Biotechnology, Inc. The mouse TNFα cytoset ELISA kit was from Invitrogen. cAMP was quantified in macrophage lysates using the cAMP Biotrak EIA (non-acetylation protocol) from GE Healthcare according to the manufacturer’s protocol. RNA was isolated using the on-column DNase treatment with the RNeasy mini kit from Qiagen.

### Mouse Strains

Pathogen-free Balb/c mice were obtained from Harlan Sprague Dawley. cPLA_2_α^-/-^ mice were generated as previously described and backcrossed onto a Balb/c background for 10 generations [24]. The TLR4 mutant mouse strain C3H/HeJ and control strain C3H/HeOuJ were obtained from The Jackson Laboratory. TLR2^-/-^ (C57BL/6) and MyD88^-/-^ mice (C57BL/6/129) were generated as previously described [25]. MyD88^+/-^ C57BL/6/129 mice were crossed to generate MyD88^-/-^ mice and MyD88^+/+^ littermate controls. C57BL/6 control mice were obtained from The Jackson Laboratory. Dectin-1^-/-^ mice (129sv/ev) were produced as described previously [26], and age and strain matched controls obtained from Taconic. Mice were used for macrophage isolation at 7-12 wk of age.

### *C. albicans* Strains and Culture

*C. albicans* (ATCC 10261) was used for experiments unless otherwise indicated. The *C. albicans Capmr1*∆ null mutant defective in glycosylation, the re-integrant strain (*Capmr1∆+CaPMR1*) and parental wild-type control were generated as previously described [27]. *C. albicans* strains were grown on Sabouraud dextrose agar plates and maintained at 4°C.

### RPM Infection

The day before the experiment, the strains were streaked onto fresh Sabouraud dextrose agar plates and incubated overnight at 37°C. *C. albicans* was scraped from the plate and washed twice in endotoxin-free PBS. Live *C. albicans* at a multiplicity of infection (moi) of 2 was used for all experiments.

### RPM Isolation

RPM were obtained by peritoneal lavage as previously described [13]. Cells were plated at a density of 5 x 10^5^/cm^2^ (48 well plate) and incubated for 2 h at 37°C in a humidified atmosphere of 5% CO_2_ in air. After washing the cultures to remove non-adherent cells, the adherent macrophages were incubated in DMEM containing 10% heat inactivated FBS, 100 µg/ml streptomycin sulfate, 100 units/ml penicillin G, 0.29 mg/ml glutamine for 16-18 h at 37°C. The cells were washed twice with serum-free DMEM containing 0.1% human serum albumin (stimulation medium) and then infected with *C. albicans.*

### *C. albicans* Uptake and Killing assays

The ability of cPLA_2_α^+/+^ and cPLA_2_α^-/-^ RPM to bind and internalize *C. albicans* was compared using an *in vitro* recognition assay as described previously with modifications [26]. RPM were incubated for 30 min with Alex Fluor 488-labeled *C. albicans* (m.o.i. 10) prepared as described [28]. RPM were washed 3 times with stimulation media and incubated further for 1 h. Cells were lysed with 3% Triton X-100 and the fluorescence intensity was measured. The killing assay involved incubating cPLA_2_α^+/+^ and cPLA_2_α^-/-^ RPM with *C. albicans* (m.o.i. 5) for 30 min, followed by 3 washes and further incubation for 1 and 4 h. Cells were lysed with 3% Triton X-100 and the lysates streaked on Sabouraud dextrose agar plates to measure colony forming units (CFU).

### Cytokine Measurement

The culture medium was removed at the indicated times after infection of RPM with *C. albicans* and stored at -80°C for cytokine measurement and eicosanoid analysis (see below). TNFα in the culture medium was quantified by ELISA and by Luminex assay, which gave similar results.

### Mass Spectrometry Eicosanoid Analysis

The samples of culture media were thawed and mixed with an equal volume of cold methanol. Just prior to analysis they were diluted in water to a final methanol concentration of <15% and then extracted using a solid phase extraction cartridge (Strata Polymeric Reversed Phase 60 mg/ml, Phenomenex, Torrance, CA). The eluate (1 ml of methanol) was dried and reconstituted in 75 µl of HPLC solvent A (8.3 mM acetic acid buffered to pH 5.7 with NH_4_OH) and 25 µl of solvent B (acetonitrile/methanol, 65/35, v/v). An aliquot of each sample (50 µl) was injected into an HPLC and metabolites separated on a C18 column (Ascentis 15 cm x 2.1 mm, 5 µm, Supelco) eluted at a flow rate of 200 µl/min with a linear gradient from 25% to 75% solvent B in 13 min then increased to 98% in 2 min and held for 11 min. The HPLC system was directly interfaced into the electrospray ionization source of a triple quadrapole mass spectrometer (Sciex API 3000, PE-Sciex, Thornhill Ontario, Canada). Mass spectrometric analyses were performed in the negative ion mode using multiple reaction monitoring (MRM) for specific analytes. Deuterated internal standards were detected using the following transitions: *m/z* 355→275 for [d_4_]PGE_2_, *m/z* 373→167 for [d_4_]6-keto-PGF_1_α, *mz* 311→213 and *mz* 629→272 for [d_4_]LTC_4_. Eicosanoids were detected centered in specific retention time (RT) windows using the following transitions and limits of quantitation: PGE_2_, RT 9.3 min, *m/z* 351→271, 8 pg/ml; 6-keto-PGF_1_α, RT 6.4 min, *m/z* 369→163, 40 pg/ml and LTC_4_, RT 10.1 min, *m/z* 624→272, 40 pg/ml. MRM chromatograms using a similar analytic scheme have previously been described [29]. Quantitative results were calculated by determining the ratio of the signal of an analyte to that for an internal standard and comparing to a standard isotope dilution curve [30].

### Western Blots

To prepare lysates for western blots, cell monolayers were washed twice in ice cold PBS and then scraped in lysis buffer: 50 mM Hepes, pH 7.4, 150 mM sodium chloride, 10% glycerol, 1% Triton X-100, 1 mM EGTA, 1 mM EDTA, 200 µM sodium vanadate, 10 mM tetrasodium pyrophosphate, 100 mM sodium fluoride, 300 nM p-nitrophenyl phosphate, 1 mM phenylmethylsulfonylfluoride, 10 µg/ml leupeptin, and 10 µg/ml aprotinin. After incubation on ice for 30 min, lysates were centrifuged at 15,000 rpm for 15 min and protein concentration in the supernatant determined by the bicinchoninic acid method. Lysates were boiled for 5 min after addition of Laemmli electrophoresis sample buffer, and then proteins were separated on 10% SDS-polyacrylamide gels. After transfer to nitrocellulose membrane, samples were incubated in blocking buffer (20 mM Tris-HCl, pH 7.6, 137 mM NaCl, 0.05% Tween (TTBS)) containing 5% nonfat milk for 1 h, and then incubated overnight at 4°C with primary antibodies in TTBS. The membranes were incubated with anti-rabbit IgG horseradish peroxidase antibody (1:5000) in TTBS for 30 min at room temperature. The immunoreactive proteins were detected using the Amersham ECL system.

### Microarray Analysis

RPM cultured and stimulated with *C. albicans* for 3 h as described above were washed twice with endotoxin-free PBS and total RNA isolated. Template RNA quality was assessed with the Agilent Bioanalyzer 2100 and an Agilent Nano RNA 6000 kit per the Agilent protocol. RNA quality ranged from a RNA Integrity Number (RIN) of 8.1 to 10.0. An Agilent Quick Amp Labeling kit was used to generate Cy3 labeled RNA. Yields of 3.7-6.8 µg were obtained with specific activities of 7.5-9.4 pmol/µg. Fragmentation followed by hybridization was performed (Agilent Gene Expression Hybridization Kit) on Agilent Whole Mouse Genome kit 4x44 microarray slides at 65^°^C for 16 hr. Slides were washed according to the Agilent Quick Amp Labeling Kit protocol and scanned immediately on an Agilent G2505B scanner. The microarray results were log base 2 transformed and data normalization was applied using the 75% percentile shift method to adjust for experimental variability. Boxplots of resulting expression were examined for consistency and all quality control metrics were within acceptable ranges. Filtering was performed to exclude gene expression probes that did not reach a relative expression value of ≥35 across all groups. Microarray samples were grouped by unstimulated cPLA_2_α^-/-^ RPM, *C. albicans*-stimulated cPLA_2_α^-/-^ RPM, unstimulated cPLA_2_α^+/+^ RPM and *C. albicans*-stimulated cPLA_2_α^+/+^ RPM. Differences between *C. albicans*-stimulated cPLA_2_α^+/+^ and *C. albicans*-stimulated cPLA_2_α^-/-^ RPM were compared using Student’s unpaired t-tests, while comparisons for unstimulated cPLA_2_α^+/+^ and *C. albicans*-stimulated cPLA_2_α^+/+^ RPM were evaluated using paired t-tests. For evaluating differential gene expression between *C. albicans*-stimulated cPLA_2_α^+/+^ and *C. albicans*-stimulated cPLA_2_α^-/-^ RPM, genes that were not significantly affected by *C. albicans* treatment (p<0.05) in both cPLA_2_α^+/+^ and cPLA_2_α^-/-^ RPM were excluded from the analysis. All processing and analyses were performed in Genespring GX 11.5 (Agilent Technologies, Santa Clara, CA). The data were analyzed using the DAVID bioinformatics resource to evaluate the functional clustering of genes [31]. The complete microarray results can be accessed in the Gene Expression Omnibus (GEO; www.ncbi.nlm.nih.gov/geo/) of the National Center for Biotechnology Information using the GEO Series accession number GSE46533.

### Real-time PCR

RPM were isolated from cPLA_2_α^+/+^ and cPLA_2_α^-/-^ mice, cultured as described above, and RNA isolated at 1, 3 and 6 h after stimulation with *C. albicans*. RNA concentration and purity were determined by UV spectrophotometry, and RNA integrity verified using an Agilent Bioanalyzer 2100. cDNA was synthesized from RNA (200 ng) using RT^2^ First Strand kit (SA Biosciences). Real-time PCR was performed using RT^2^ qPCR Mastermix and custom-made RT^2^ Profiler PCR Array System according to the manufacturer’s protocol using the StepOnePlus Real-Time PCR System (Applied Biosystems). PCR arrays in a 96-well format were used containing pre-validated primers tested for efficiency (SA Biosciences). The RT^2^ Profiler PCR Array System included a reverse transcription control preloaded into the primer buffer of the RT^2^ First Strand cDNA synthesis kit that measured the relative efficiency of the reverse transcription for all the samples. A genomic DNA control and a positive PCR control were also included in the system. The RT^2^ Profiler PCR Array data were normalized to two housekeeping genes *Gapdh* and *Hprt* and the relative gene expression level (2^(-ΔC_t_) was calculated using the formula ΔC_t_= C_t_ (gene of interest)-C_t_ (housekeeping gene). The data were analyzed on the PCR array data analysis SA Biosciences web portal (http://pcrdataanalysis.sabiosciences.com/pcr/arrayanalysis.php).

Real-time PCR was also performed with cDNA synthesized with random hexamer primers (Superscript III polymerase, Invitrogen) using TaqMan fast universal PCR master mix. TaqMan assay probes used were:

*Csf1* (1Mm00432686_m1),
*Csf3* (Mm00438335_g1),
*Tnf* (Mm99999068_m1),
*Il10* (Mm00439614_m1),
*Nr4a2* (Mm00443060_m1),
*Crem* (Mm00516346_m1),
*Stat3* (Mm01219775_m1) and

*Gapdh* (Mm99999915_g1). The housekeeping gene *Gapdh* and a calibrator containing mRNA from unstimulated cPLA_2_α^+/+^ and cPLA_2_α^-/-^ RPM were used for normalization. Threshold cycle values (*C*_*T*_) were determined and used for ∆∆C_T_ analysis of gene expression [32].

## Results

**Figure 1:**
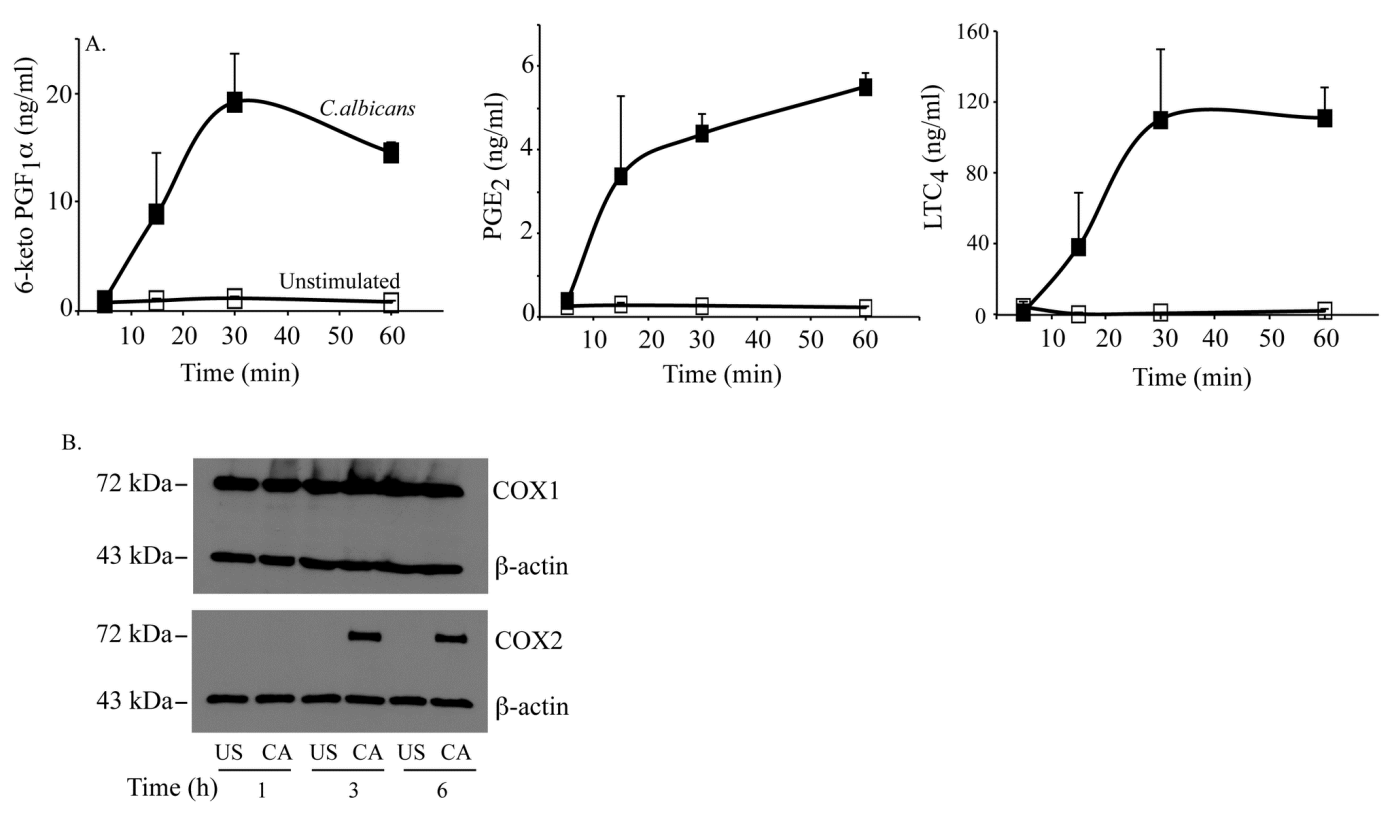
Time course of *C. albicans*-stimulated eicosanoid production. (A) RPM were incubated with *C. albicans* for the indicated times. The culture medium from unstimulated (open squares) or *C. albicans*-stimulated (closed squares) RPM was analyzed for eicosanoids by mass spectrometry. The data are the average of triplicate samples (±S.D.) from a representative experiment. (B) Cell lysates from unstimulated RPM (US) or RPM stimulated with *C. albicans* (CA) for 1, 3 and 6 h were analyzed for COX1 and COX2 expression by Western blotting. Sample loading was evaluated by probing with antibodies to β-actin.

The production of eicosanoids by RPM is initiated by the activation of cPLA_2_α, which occurs rapidly in response to *C. albicans* or zymosan due to post-translational processes [9–12]. The major arachidonic acid metabolites produced by RPM in response to *C. albicans* and zymosan are PGI_2_, PGE_2_, and LTC_4_, and their production is dependent on cPLA_2_α activation to provide arachidonic acid substrate [12–14]. As shown in Figure 1A, eicosanoids were produced most rapidly during the first 30 min after *C. albicans* addition. Prostaglandin production occurred before the increase in COX2 expression stimulated by *C. albicans*, which was detected 3 h after addition of *C. albicans* but not at 1 h (Figure 1B). In contrast, COX1 was constitutively expressed in RPM and expression was not affected by *C. albicans* infection. Microarray data also confirmed that COX2 expression was very low compared to COX1 in unstimulated cPLA_2_α^+/+^ RPM, but there was a significant increase in expression of COX2 (*Ptgs2*) but not COX1 (*Ptgs1*) in cPLA_2_α^+/+^ RPM treated with *C. albicans* for 3 h (Table 1). The results suggest that cPLA_2_α-mediated release of arachidonic acid couples to COX1 for early production of prostaglandins.

**Table 1:** Relative expression values of cyclooxygenases and prostaglandin receptors in RPM.

**Official Symbol**	**Entrez_Gene_ID**	**Unstimulated**	***C. albicans*-treated**
		Mean Expression	Mean Expression
Ptgs2*	19225	67 ± 14	11243 ± 2938
*Ptgs1*	19224	4694 ± 2731	2027 ± 655
*Ptger2*	19217	204 ± 18	177 ± 65
*Ptger4*	19219	257 ± 24	248 ± 50
Ptgir*	19222	589 ± 217	1168 ± 179

cPLA_2_α^+/+^ RPM were stimulated with *C. albicans* for 3 h and gene expression determined by microarray analysis. The * denotes a significant (p<0.05) increase in expression by *C. albicans* treatment.

### Role of cPLA_2_α in regulating TNFα production

**Figure 2:**
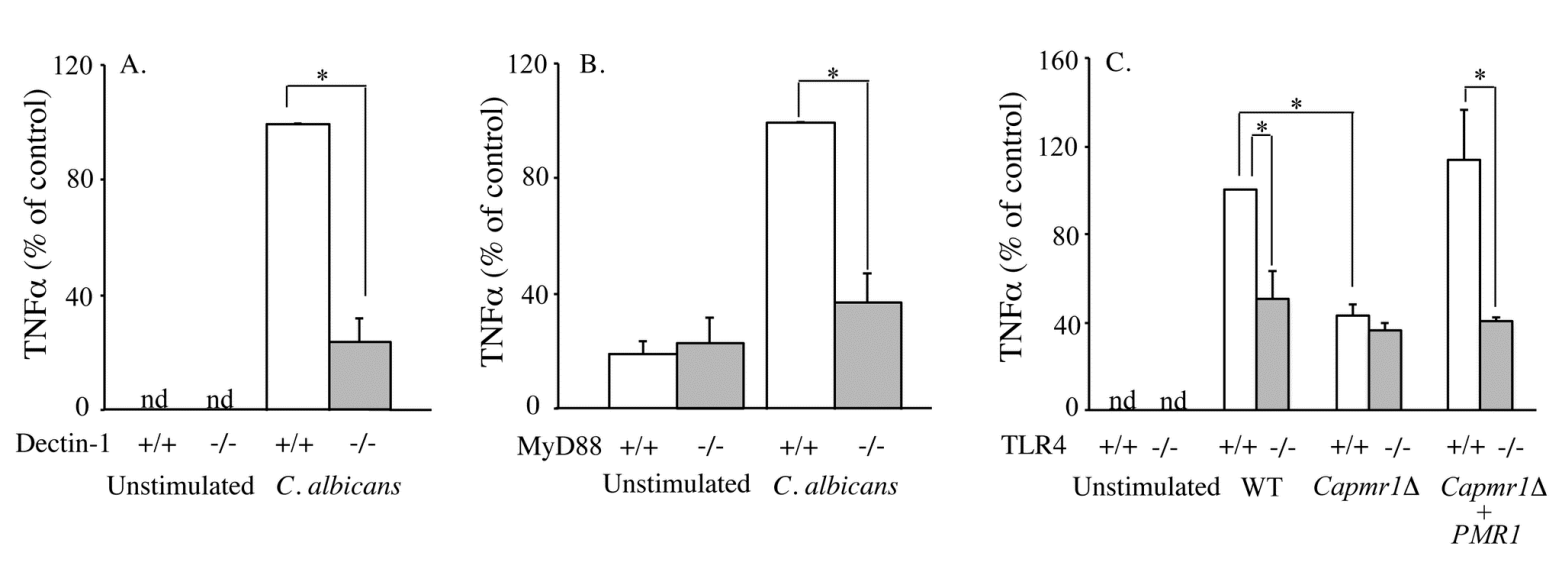
Role of PRRs in regulating *C. albicans*-stimulated TNFα production. Wild type (open bars) and Dectin-1^-/-^ (A), MyD88^-/-^ (B) and TLR4^-/-^ (C) RPM (shaded bars) were incubated with *C. albicans* for 6 h. In panel C, RPM were stimulated with the parental wild type *C. albicans* (WT), the *Capmr1∆* null mutant and the re-integrant strain (*Capmr1∆+CaPMR1*). The data are the average of 3 experiments ±S.E. (*p<0.05). Levels of TNFα in the culture medium were determined by ELISA.

The initial focus was to determine if cPLA_2_α activation regulates TNFα production in *C. albicans*-stimulated RPM since prostaglandins can suppress production of this pro-inflammatory cytokine as we reported for *L. monocytogenes*-stimulated RPM [8,33,34]. First we investigated if TNFα production was mediated by similar PRRs that promote cPLA_2_α activation in response to *C. albicans*. We reported that dectin-1 and MyD88, but not TLR2 or TLR4, play a role in the activation of cPLA_2_α in response to *C. albicans* [13,14]. We found that production of TNFα 6 h after addition of *C. albicans* was reduced in dectin-1^-/-^ and MyD88^-/-^ RPM compared to dectin-1^+/+^ and MyD88^+/+^ RPM (Figure 2A and 2B). The requirement for MyD88 suggested a role for TLRs. A comparison of RPM from TLR2^+/+^ and TLR2^-/-^ mice showed that TNFα production was not mediated by TLR2 (data not shown). However, TLR4 partially contributed to *C. albicans*-mediated TNFα production, which was reduced by approximately 50% in TLR4^-/-^ RPM (Figure 2C). Since mannans of *C. albicans* cell wall engage TLR4 we tested the ability of the *C. albicans* glycosylation mutant (*Capmr1∆* null mutant), which is devoid of phosphomannans and has defective N- and O-linked mannans, to stimulate TNFα production in TLR4^+/+^ and TLR4^-/-^ RPM [27,35]. Compared to TLR4^+/+^ RPM treated with wild type *C. albicans*, TNFα production in TLR4^+/+^ RPM treated with *Capmr1*∆ null mutant was reduced by about 50% similar to the level observed in TLR4^-/-^ RPM stimulated with wild type *C. albicans* (Figure 2C). TNFα production by TLR4^+/+^ RPM was restored when the *CaPMR1* gene was reintegrated into the mutant strain (*Capmr1*∆*+CaPMR1*). Therefore PRRs on RPM that engage cell wall mannans and β-glucans contribute to TNFα production. Since cPLA_2_α^+/+^ and cPLA_2_α^-/-^ RPM were used to determine the role of cPLA_2_α in regulating gene expression in response to *C. albicans* (as described below), we compared their levels of expression of PRRs involved in *C. albicans* recognition. Microarray data showed that cPLA_2_α^+/+^ and cPLA_2_α^-/-^ RPM express similar levels of PRRs Clec7a (dectin-1), Clec4n (dectin-2), Tlr4 and Tlr2 (Gene Expression Onmibus, www.ncbi.nlm.nih.gov.geo/, GSE46533). We also compared the ability of cPLA_2_α^+/+^ and cPLA_2_α^-/-^ RPM to bind and internalize *C. albicans*. Results of a recognition assay demonstrated no differences in the uptake of Alex Fluor-labeled *C. albicans* by cPLA_2_α^+/+^ and cPLA_2_α^-/-^ RPM (data not shown). A *C. albicans* killing assay was also carried out by incubating RPM with *C. albicans* and then measuring the recovery of CFU from RPM after further incubation for 1 and 4 h. There were no differences in CFU recovered at 1 h in WT and cPLA_2_α^-/-^ RPM. However at 4 h there was a small but significantly higher level of *C. albicans* CFU recovered from cPLA_2_α^-/-^ RPM. In three independent experiments the CFU in cPLA_2_α^-/-^ RPM was 172%±32%, p<0.002 compared to cPLA_2_α^+/+^ RPM (100%). The results suggest that the cPLA_2_α^+/+^ RPM have a slightly greater ability to kill internalized *C. albicans*.

**Figure 3:**
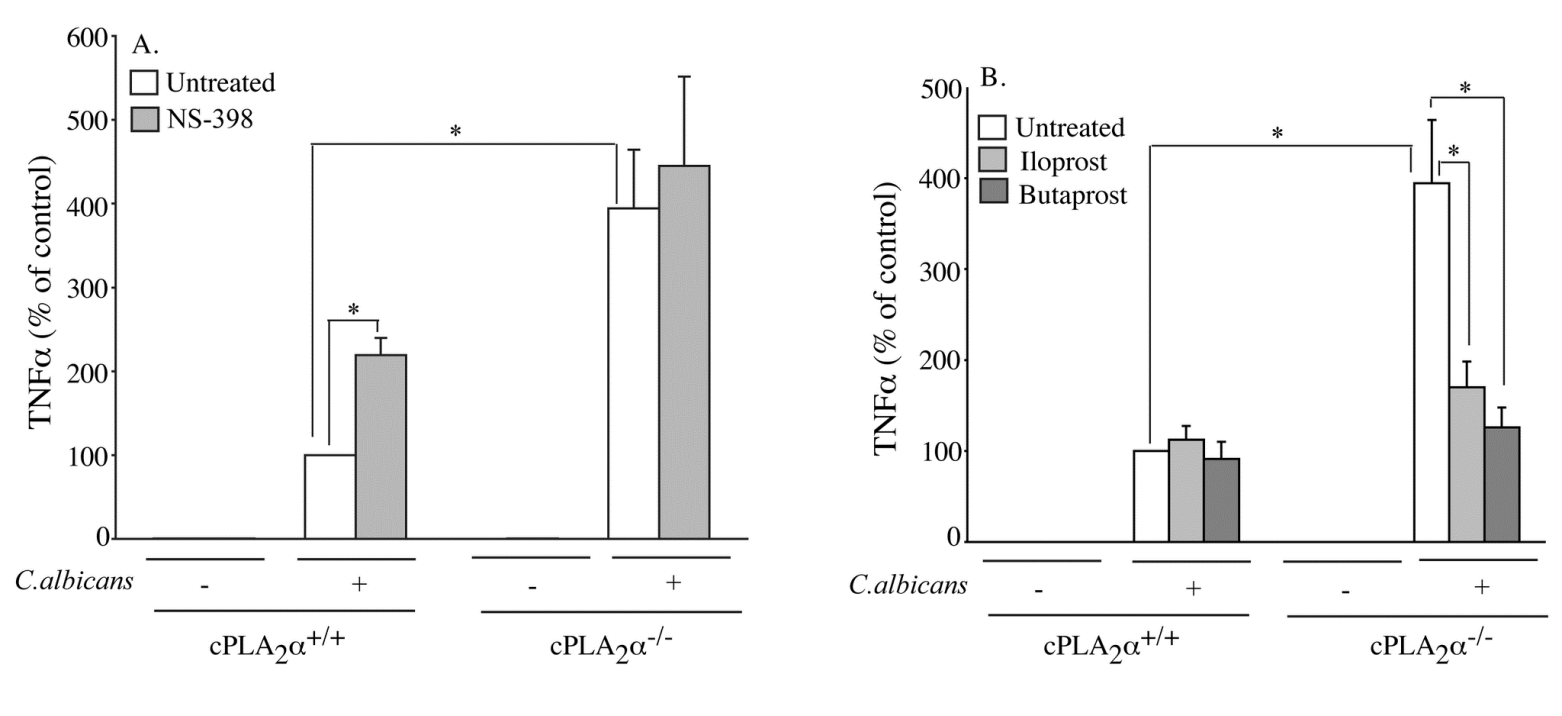
Role of prostaglandins in regulating *C. albicans*-stimulated TNFα production. cPLA_2_α^+/+^ and cPLA_2_α^-/-^ RPM were incubated with (A) NS-398 (10 µM), or (B) iloprost (1 µM) or butaprost (10 µM) for 30 min followed by incubation with *C. albicans* for 6 h. Levels of TNFα in the culture medium were determined by ELISA. The data are the average of 3 experiments ±S.E. (*p<0.05).

The role of cPLA_2_α activation and prostanoid production in regulating the production of TNFα in response to *C. albicans* was investigated by comparing RPM from cPLA_2_α^+/+^ and cPLA_2_α^-/-^ mice, and by treating the macrophages with a cyclooxygenase inhibitor NS398 (Figure 3A). The production of TNFα was lower in cPLA_2_α^+/+^ RPM compared to cPLA_2_α^-/-^ RPM measured 6 h after *C. albicans* addition. NS398 treatment enhanced TNFα production in cPLA_2_α^+/+^ but not in *C. albicans*-stimulated cPLA_2_α^-/-^ RPM suggesting that prostanoids suppress TNFα expression. NS398 completely blocked production of PGE_2_ and PGI_2_ in RPM stimulated with *C. albicans* for 6 h (data not shown), and at the concentration used (10 µM) inhibits both murine COX1 and COX2 [36]. To further investigate the role of prostanoids in the autocrine regulation of TNFα production, RPM were treated with agonists for the PGE_2_ receptor EP_2_ (butaprost) and the PGI_2_ receptor IP (iloprost) (Figure 3B). Microarray data showed that RPM express the IP receptor (*Ptgir*), the EP2 (*Ptger2*) and EP4 (*Ptger4*) receptors (Table 1). The agonists had no effect on the levels of TNFα produced by cPLA_2_α^+/+^ RPM that produce endogenous prostaglandins in response to *C. albicans* (Figure 3B). However, the higher level of TNFα produced by *C. albicans*-stimulated cPLA_2_α^-/-^ RPM, which do not produce endogenous prostaglandins, was reduced by the receptor agonists to the level produced by cPLA_2_α^+/+^ RPM. The data suggest that prostaglandins acting through the EP2 and IP receptors suppress TNFα production since it is enhanced by inhibiting prostaglandin production in *C. albicans*-stimulated cPLA_2_α^+/+^ RPM and suppressed by prostaglandin receptor agonists in cPLA_2_α^-/-^ RPM.

**Figure 4:**
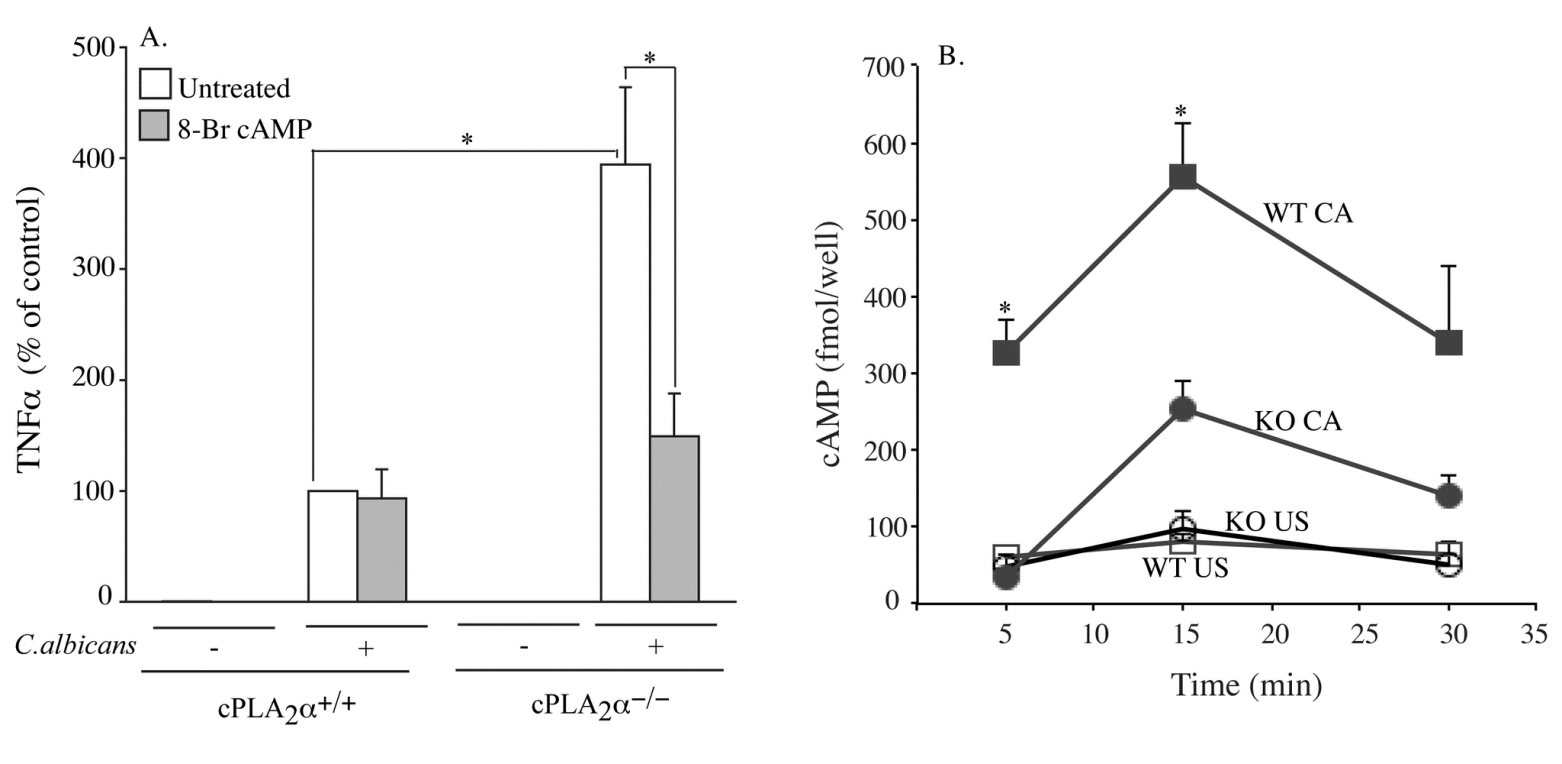
cAMP production is enhanced by cPLA_2_α activation and suppresses TNFα production. (A) cPLA_2_α^+/+^ and cPLA_2_α^-/-^ RPM were incubated with 8-Br-cAMP (1 mM) for 30 min followed by incubation with *C. albicans* for 6 h. Levels of TNFα in the culture medium were determined by ELISA. (B) cPLA_2_α^+/+^ (WT, squares) and cPLA_2_α^-/-^ RPM (KO, circles) were incubated with (closed symbols) or without (open symbols) *C. albicans* (CA) for the indicated times. Cell lysates were processed for cAMP determinations as described in Experimental Design. The data are the average of 3 experiments ±S.E. (*p<0.05). In panel B, CA treated WT vs. CA treated KO at 5 and 15 min are compared for significance.

The EP_2_ and IP receptors mediate increases in cAMP, which is implicated in regulating *Tnfα* gene expression [37,38]. As shown in Figure 4A, the stable cAMP analogue 8-Br-cAMP suppressed *C. albicans*-stimulated TNFα production in cPLA_2_α^-/-^ RPM, as observed for the prostanoid receptor agonists, but had no effect on the lower level of TNFα produced by cPLA_2_α^+/+^ RPM. The results suggest that prostaglandins produced by *C. albicans*-stimulated cPLA_2_α^+/+^ RPM act in an autocrine manner through prostaglandin receptors that increase cAMP to suppress TNFα production. This is supported by results showing that levels of cAMP were higher in cPLA_2_α^+/+^ RPM than cPLA_2_α^-/-^ RPM within 5-30 min after *C. albicans* addition (Figure 4B).

### Effect of *C. albicans* on gene expression in RPM

We next determined the effect of *C. albicans* on global gene expression in RPM by microarray and then evaluated how cPLA_2_α activation modulates the transcriptional response. *C. albicans* stimulated an increase in expression of 427 genes (≥4.0-fold, p<0.05, n=3) in cPLA_2_α^+/+^ Balb/c RPM at 3 h. Relative expression levels for these genes and the fold change in response to *C. albicans* are shown in Table S1A. Many of the genes that increase in response to *C. albicans* represent the common host-response that is induced in many cell types by a variety of infectious agents [39]. The data were analyzed using the DAVID bioinformatics resource to evaluate the functional clustering of genes that were increased in RPM in response to *C. albicans* [31]. The most highly enriched clusters contained genes in apoptosis, cytokines, wound and inflammatory responses, regulation of phosphorylation and protein kinase activity, cell motion, vascular development, regulation of cytokine production, MAP kinase phosphatase activity, regulation of transcription and growth factor activity (Table 2). *Csf3* was the most highly induced gene by *C. albicans* (>600-fold) (Table S1A). The cytokine CSF3 regulates the production and function of neutrophils and is important for host defense against *C. albicans* [40,41]. As discussed below, the expression of *Csf3* was regulated by cPLA_2_ activation. There were 110 genes down-regulated in RPM at 3 h by *C. albicans* (≥4-fold, p<0.05, n=3) (Table S1B). The clusters for the down-regulated genes had very low enrichment scores compared to the up-regulated genes when subject to DAVID analysis (data not shown).

**Table 2:** Functional annotation clusters of genes induced in *C. albicans*-stimulated RPM.

**Annotation Clusters**	**Official Symbol**
Apoptosis	*Bcl2l11, Cflar, Cd24a, Chac1, Ddit4, Egin3, Epha2, Rybp, Traf1, Traf5, Ahr, F2r, Csrnp1, Fem1b, Gzmb, Gadd45b, Gadd45g, Id1, Il6, Jmjd6, Malt1, Myc, Niacr1, Nfκb1, Nr4a2, Osm, Phlda1, Bnip3, Blcap, Ppp1r15a, Srgn, Siah2, Mcl1, Trib3, Trim69, Tnf, Tnfrsf12a, Tnfaip3, Unc5b, Zc3h12a*
Cytokines, Response to wounding, and Inflammatory response	*Areg, Cd24a, Kdm6b, Bmp2, Bmp6, Ccl2, Ccl3, Ccl4, Ccl7, Ccr1, Cxcl1, Cxcl2, Cxcl3, Csf2, Csf3, F2r, F3,Gdf15, Gja1, Hbegf, Id3, Il1a, Il1b, Il1f6, Il10, Il23a, Il6, Nfkbid, Olr1, Osm, Plaur, Plek, Procr, Proz, Slc7a2, Sphk1, Tnf, Tnfsf9*
Regulation of phosphorylation and protein kinase activity	*Cd24a, Adora2a, Bmp2, F2r, Cdkn1a, Cish, Dgkg, Dusp16, Edn1, Ereg, Gadd45b, Gadd45g, Il1b, Il6, Lrp8, Laper1, Nrg1, Osm, Reln, Spag9, Sphk, Spry2, Socs3, Trib3, Tnf*
Cell motion	*Cd24a, Epha4, Alcam, Ccr1, Cxcl2, Cxcl3, Egr2, Gja1, Hbegf, Il1b, Lrp8, Nrg1, Nr4a2, Plau, Pdpn, Pvr, Reln, Runx3, Zfand5, Tes, Tnf, Tnfrsf12a, Vegfa*
Vascular development	*Epha2, Junb, Smad7, Edn1, Efnb2, Ereg, Gja1, Hbegf, Id1, Itgav, Il1b, Jmjd6, Pdpn, Prok2, Zfand5, Sphk1, Socs3, Tgm2, Tnfrsf12a, Vegfa, Zc3h12a, Zfp36l1*
Regulation of cytokine production	*Cd24a, Cd83, Adora2a, Adora2b, F2r, Edn1, Ereg, Fst, Inhbb, Irf4, Il1a, Il1b, Il10, Il6, Nfκb1, Prok2, Rel, Srgn, Sphk1, Tnf*
MAP kinase phosphatase activity	*Dusp1, Dusp2, Dusp4, Dusp8, Dusp10, Dusp14, Dusp16*
Regulation of transcription from RNA polymerase II promoter	*Eaf1, Kdm6b, Mxi1, Pou3f1, Rybp, Skil, Atf4, Ahr, Bmp2, Csrnp1, Egr1, Egr2, Fosl1, Hes1, Inhba, Id1, Id2, Id3, Irf4, Il6, Jarid2, Med13, Myc, Nfκb1, Nufip1, Nr4a1, Nr4a2, Nr4a3, Osm, Plagl1, Pbx1, Sap30, Tnf, Vegfa*
Growth factor activity	*Areg, Bmp2, Bmp6, Cxcl1, Csf2, Csf3, Ereg, Gdf15, Hbegf, Inhba, Inhbb, Il1b, Il6, Vegfa*

Genes expressed at higher levels (427 genes, ≥4-fold, <0.05) in *C. albicans*-stimulated cPLA_2_α^+/+^ RPM were analyzed using DAVID bioinformatics resource.

### Genes expressed at lower levels in *C. albicans*-stimulated cPLA_2_α^+/+^ than cPLA_2_α^-/-^ RPM

Differential gene expression was compared in cPLA_2_α^+/+^ and cPLA_2_α^-/-^ RPM treated with *C. albicans* for 3 h. We chose to study the effect of *C. albicans* infection on gene expression at 3 h in order to evaluate the role of cPLA_2_α activation and eicosanoids in regulating early responses during the acute phase of infection. The regulation of gene expression at later times becomes more complicated due to autocrine effects of the products of early response genes that promote induction of a second wave of gene induction. In cPLA_2_α^+/+^ RPM, 86 genes were expressed at lower levels and 181 genes at higher levels than cPLA_2_α^-/-^ RPM (≥2-fold, p<0.05, n=3) (Tables S2A and S2B, respectively). When genes expressed at lower levels in cPLA_2_α^+/+^ RPM were analyzed using DAVID, they grouped into functional clusters involving GTP binding, regulation of cytokine production/cytokine receptor interaction and regulation of proliferation (Table 3). The expression of genes for GTP binding proteins included several IFNγ-inducible GTPases (guanylate binding proteins (Gbp) *1*, *2*, *3*, *5*, 6 and 7; immunity-related GTPase family M members (*Irgm*) 1 and 2; IFNγ-inducible protein (*Ifi*) *47* and IFNγ-inducible GTPase (*Iigp*) *1*). Some of these genes regulate host defense to microbial infection although their function is poorly understood [42–45]. Several genes expressed lower in *C. albicans*-stimulated cPLA_2_α^+/+^ than cPLA_2_α^-/-^ RPM in the cytokine cluster (Table 3) are pro-inflammatory such as the chemokine *Cx3cl1* (fracktalkine), *Cd40*, *Tnfα* and *Ifnγ* [46–48]. The lower expression of *Ifnγ* in cPLA_2_α^+/+^ RPM correlated with the reduced expression of the IFNγ regulated GTPases, although its level of expression in RPM was very low (Table S2A). Genes for the transcription factors, interferon regulatory factors (Irf) *1* and *Irf4* (Cytokine cluster), and the AT-hook transcription factor (*Akna*) were also expressed at lower levels in cPLA_2_α^+/+^ than cPLA_2_α^-/-^ RPM (Table S2A). AKNA promotes Cd40 expression suggesting a correlation between low expression of *Akna* and *Cd40* in cPLA_2_α^+/+^ RPM [49]. AKNA functions in inflammation and cancer [50]. There was also a correlation with the lower expression of genes for guanylate binding proteins (Gbp) and *Tnfα* in cPLA_2_α^+/+^ RPM and their transcriptional regulator *Irf1* [51]. IRF transcription factors play important roles in host defense and regulating immune responses [52].

**Table 3:** Functional annotation clusters of genes expressed at lower levels in *C. albicans*-stimulated cPLA_2_α^+/+^ than cPLA_2_α^-/-^ RPM.

**Annotation Clusters**	**Official Symbol**
GTP binding	*Rab33A, Rasd2, Gbp1, Gbp2, Gbp3, Gbp5, Gbp6, Gbp7, Irgm1, Irgm2, Ifi47, Iigp1, Ak4*
Regulation of cytokine production, Cytokine receptor interaction	*Cd40, Ccl5, Cx3cl1, Csf1, Infg, Irf1, Irf4, Il15ra, Ticam2, Il20rb, Tnfrsf14, Tnf*
Regulation of proliferation	*Cd40, Adm, Csf1, Edn1, Igf1, Infg, Il20rb, Lst1, Plau, Smo, Tnfrsf14, Tnf*

Genes expressed at lower levels (86 genes, ≥2-fold, <0.05) in cPLA_2_α^+/+^ than cPLA_2_α^-/-^ RPM stimulated for 3 h with *C. albicans* were analyzed using DAVID bioinformatics resource.

cPLA_2_α^+/+^ RPM expressed lower mRNA levels of the chemokine *Ccl5* (Cytokine cluster), which promotes the trafficking of cells to sites of inflammation [53]. PGE_2_ suppresses CCL5 production in macrophages and dendritic cells thus dampening inflammation and immune responses [54]. Colony stimulating factor 1 (Csf1, Cytokine cluster) was induced to a greater extent in cPLA_2_α^-/-^ RPM (10-fold) than cPLA_2_α^+/+^ RPM (3-fold) (Table S2A). It promotes macrophage-lineage development but also recruits myeloid cells during inflammation and infection, and promotes their survival [55]. Another pro-inflammatory gene expressed at lower levels in cPLA_2_α^+/+^ RPM was the vasoactive peptide endothelin 1 (*Edn1*, Regulation of proliferation cluster), which stimulates myeloid and mast cells at sites of inflammation [56] (Table S2A). Overall the results implicate cPLA_2_α activation and eicosanoid production in suppressing the expression of pro-inflammatory genes, and transcription factors that regulate their expression.

**Figure 5:**
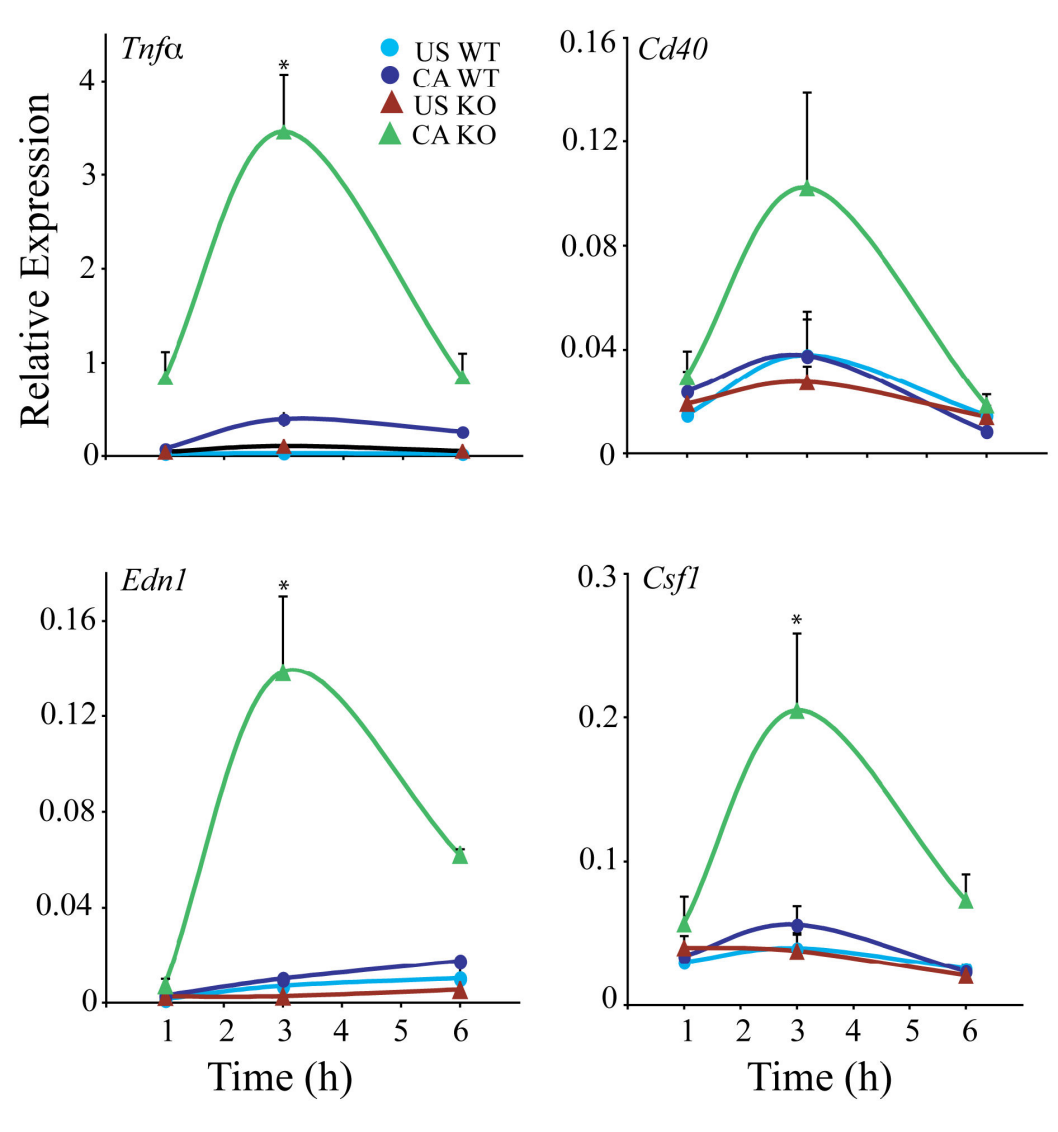
Time course of expression of genes expressed at lower levels in *C. albicans*-stimulated cPLA_2_α^+/+^ than cPLA_2_α^-/-^ RPM. cPLA_2_α^+/+^ (WT, circles) and cPLA_2_α^-/-^ (KO, triangles) RPM were incubated with (CA) or without (US) *C. albicans* for the indicated times. RNA was isolated and gene expression determined by real-time PCR using the RT^2^ Profiler PCR Array System (SA Bioscience) as described in Experimental Design. The data were normalized to the housekeeping genes *Gapdh* and *Hprt*. The results are the average of 3 experiments ±S.E. Gene expression in *C. albicans* infected WT at 3 h was compared to *C. albicans* infected KO at 3 h to determine significance (*p<0.05).

We corroborated the microarray results by real-time PCR for representative genes expressed lower in cPLA_2_α^+/+^ than cPLA_2_α^-/-^ RPM. Their expression was preferentially enhanced by *C. albicans* in cPLA_2_α^-/-^ RPM compared to cPLA_2_α^+/+^ RPM suggesting that products of cPLA_2_α activation suppress their expression (Figure 5). Results of real time PCR showed that expression of these genes was transient in cPLA_2_α^-/-^ RPM occurring maximally 3 h after stimulation with *C. albicans.*

### Genes expressed at higher levels in *C. albicans*-stimulated cPLA_2_α^+/+^ than cPLA_2_α^-/-^ RPM

A larger number of genes were expressed at higher levels in cPLA_2_α^+/+^ RPM than cPLA_2_α^-/-^ RPM (181 genes, ≥2-fold, p<0.05, n=3) (Table S2B). From DAVID analysis, genes clustered in functional groups involving vascular development, embryonic morphogenesis, sexual reproduction, response to wounding, inflammatory and defense responses, growth factors and growth factor activity, DNA binding and transcription regulation, and disulfide bond (Table 4). Several genes in these clusters are associated with cancer development consistent with a role for prostaglandins in promoting carcinogenesis [57,58]. These include the Eph receptor A2 tyrosine kinase (*EphA2*, Vascular development cluster), the epidermal growth factor receptor (EGFR) ligands epiregulin (*Ereg*) and amphiregulin (*Areg*) (Growth factor cluster), the transmembrane glycoprotein podoplanin (*Pdpn*) and its transcriptional regulator the homeobox protein *Prox1* (Vascular development cluster), the chemokine receptor 7 (*CxCr7*, Disulfide bond cluster), matrix metalloproteinase 13 (*Mmp13*, Embryonic morphogenesis cluster) and its transcriptional regulators *Runx2* and nuclear receptor subfamily 4, group A, member 2 (*Nr4a2*). These genes expressed at higher levels in cPLA_2_α^+/+^ RPM promote angiogenesis, tumor growth and invasion, and are regulated by prostaglandins and cAMP [59–66].

**Table 4:** Functional annotation clusters of genes expressed at higher levels in *C. albicans*-stimulated cPLA_2_α^+/+^ than cPLA_2_α^-/-^ RPM.

**Annotation Clusters**	**Official Symbol**
Vascular development	*Eph2, Chd7, Ereg, Foxc1, Gja1, Itgav, Lepr, Nus1, Pdpn, Prox1, S1pr1, Socs3, Zfp36l1*
Embryonic morphogenesis	*Eph2, Chd7, Chst11, Foxc1, Hes1, Il10, Mmp13, Pbx1, Prox1, Socs3, Spry2, Jag2*
Sexual reproduction	*Bcl6, Bcl2l11, Crem, Calca, Cadm1, Ereg, Fst, Foxc1, Jag2, Lepr, Pvrl3, Rgs2, Stat3*
Response to wounding, Inflammatory and Defense responses	*Bmp6, Cd14, Calca, Ddah2, Entpd1, Gja1, Hdac5, Il1f6, Il10, Saa1, Saa2, Stat3, Thbd, Thbs1*
Growth factors, GF activity	*Areg, Bmp6, Chst11, Csf3, Ereg, Foxc1, Gja1, Hgf, Inhbb, Jag2*
DNA binding, Transcription regulation	*Arid3b, Bcl6, Bach2, Gata2, Lhx8, Mxd1, Mxi1, Setbp1, Thap2, Crem, Chd7, Dedd2, Foxc1, Hes1, Hdac5, Lrrfip1, Nr4a2, Pbx1, Prox1, Runx2, Stat3, Fosl2, Sbno2, Tshz3, Tle1, Mafb, Zfp36, Zfp36l1*
Disulfide bond	*Nt53, Abca1, Cd14, Cd80, Edil3, Eph2, Gpr35, Areg, Antxr2, Bmp6, Calca, Cacna1d, Cadm1, Cbln3, CxCr7, Csf3, Entpd1, Ereg, Fst, Gja1, Havcr2, Hgf, Inhbb, Itgav, Il10, Jag2, Lepr, Lifr, Man1a, Mmp13, Mmp3, Mpzl1, Niacr1, Pla1a, Pvrl3, Ptger2, Lpar6, Ramp3, Sema6d, Tnfaip6, Thbd, Thbs1, Trem1, Tnfrsf9*

Genes expressed at higher levels (181 genes, ≥2-fold, <0.05) in cPLA_2_α^+/+^ than cPLA_2_α^-/-^ RPM stimulated for 3 h with *C. albicans* were analyzed using DAVID bioinformatics resource.

Of particular interest were the large number of genes expressed at higher levels in *C. albicans*-stimulated cPLA_2_α^+/+^ RPM that function to dampen inflammation. *C. albicans* induced high expression of suppressor of cytokine signaling 3 (*Socs3*, Vascular development and Embryonic morphogenesis clusters) in cPLA_2_α^+/+^ RPM (16-fold) and to a lesser extent in cPLA_2_α^-/-^ RPM (6-fold) (Table 4, Table S2B). SOCS proteins function as negative feedback inhibitory pathways to control immune cell activation and inflammation [67]. *Socs3* expression is also regulated by STAT3 (Table 4, Sexual reproduction and response to wounding clusters), which was induced 4-fold in *C. albicans*-stimulated cPLA_2_α^+/+^ RPM but not significantly affected in cPLA_2_α^-/-^ RPM (Table S2B). One of the most differentially expressed genes was *Il10* (Embryonic morphogenesis cluster) that was induced 78-fold by *C. albicans* cPLA_2_α^+/+^ RPM and 7-fold in cPLA_2_α^-/-^ RPM (Table 4, Table S2B). The expression of *Il10* is regulated in macrophages by the transcription factor PBX1 [68], also expressed at higher levels in *C. albicans*-stimulated cPLA_2_α^+/+^ than cPLA_2_α^-/-^ RPM. The anti-inflammatory response (AIR) in macrophages induced by IL10 is mediated by STAT3 through induction of the helicase family co-repressor, Strawberry notch homologue 2 (*Sbno2*) [69–71]. Expression of *Sbno2* (Table 4, DNA binding, Transcription regulation cluster) was increased in *C. albicans*-stimulated cPLA_2_α^+/+^ RPM but not in cPLA_2_α^-/-^ RPM (Table S2B).

Several genes implicated in suppressing *Tnfα* expression were expressed at higher levels in cPLA_2_α^+/+^ than cPLA_2_α^-/-^ RPM. One of these genes, the zinc finger protein 36, C3H type-like 1 (*Zfp36l1*, DNA binding cluster), was increased by *C. albicans* in cPLA_2_α^+/+^ but not cPLA_2_α^-/-^ RPM (Table 4, Table S2B), and inhibits TNFα production in macrophages by destabilizing its mRNA [72]. The cAMP responsive element modulator (Crem, also called *Icer*) (Table 4, Sexual reproduction and DNA binding cluster), was highly induced in cPLA_2_α^+/+^ RPM (16-fold) in response to *C. albicans* but not significantly affected in cPLA_2_α^-/-^ RPM (Table S2B). CREM suppresses expression of pro-inflammatory genes including *Tnfα* [73]. The anti-inflammatory and immunosuppressive neuropeptide calcitonin gene-related peptide (*Calca*, Sexual reproduction cluster) that is higher in cPLA_2_α^+/+^ than cPLA_2_α^-/-^ RPM suppresses *Tnfα* through induction of *Crem* [74,75]. *C. albicans* also induces expression of the cAMP-regulated nuclear receptor *Nr4a2* to a greater extent in cPLA_2_α^+/+^ RPM (81-fold) than in cPLA_2_α^-/-^ RPM (10-fold) (Table S2B). NR4A2 suppresses *Tnfα* expression in microglia and astrocytes [76].

Several other genes that are expressed at higher levels in cPLA_2_α^+/+^ RPM than cPLA_2_α^-/-^ RPM have diverse functions but also act to dampen inflammation (Table 4). Follistatin (*Fst*) (Sexual reproduction cluster) curbs inflammation by inactivating the inflammatory actions of activin [77]. Expression of the anti-inflammatory genes *Thbs1* and *Thbd* are also expressed higher in *C. albicans*-stimulated cPLA_2_α^+/+^ RPM than cPLA_2_α^-/-^ RPM (Table S2B) [78,79].

Several genes expressed higher in cPLA_2_α^+/+^ than cPLA_2_α^-/-^ RPM are involved in host defense such as the gap junction protein, alpha 1 (*Gja1*, Vascular development cluster) (Table 4, Table S2B). GJA1 promotes phagocytosis and host survival to bacterial infection [80]. *Csf3* (Growth factor cluster) is highly upregulated in response to *C. albicans* in cPLA_2_α^+/+^ RPM (640-fold) but to a lesser extent in cPLA_2_α^-/-^ PM (140-fold) (Table S2B). The orphan receptor triggering receptor expression on myeloid cells (*Trem1*, Disulfide bond cluster) is upregulated to a greater extent in *C. albicans*-stimulated cPLA_2_α^+/+^ (11-fold) than cPLA_2_α^-/-^ RPM (2.7-fold) (Table S2B). TREM1 couples with the signaling adaptor DAP12 and has complex effects to enhance or dampen responses to TLR activation [81]. Histidine decarboxylase (*Hdc*), the encodes the rate-limiting enzyme for histamine synthesis, is another highly differentially expressed gene that is 20-fold higher in cPLA_2_α^+/+^ than cPLA_2_α^-/-^ RPM (Table S2B). *Hdc* is transcriptionally induced in myeloid cells in response to cytokines and TLR agonists leading to immediate secretion of newly synthesized histamine [82]. Prostaglandins induce *Hdc* expression and also greatly potentiate the vasoactive effects of histamine [83–85].

**Figure 6:**
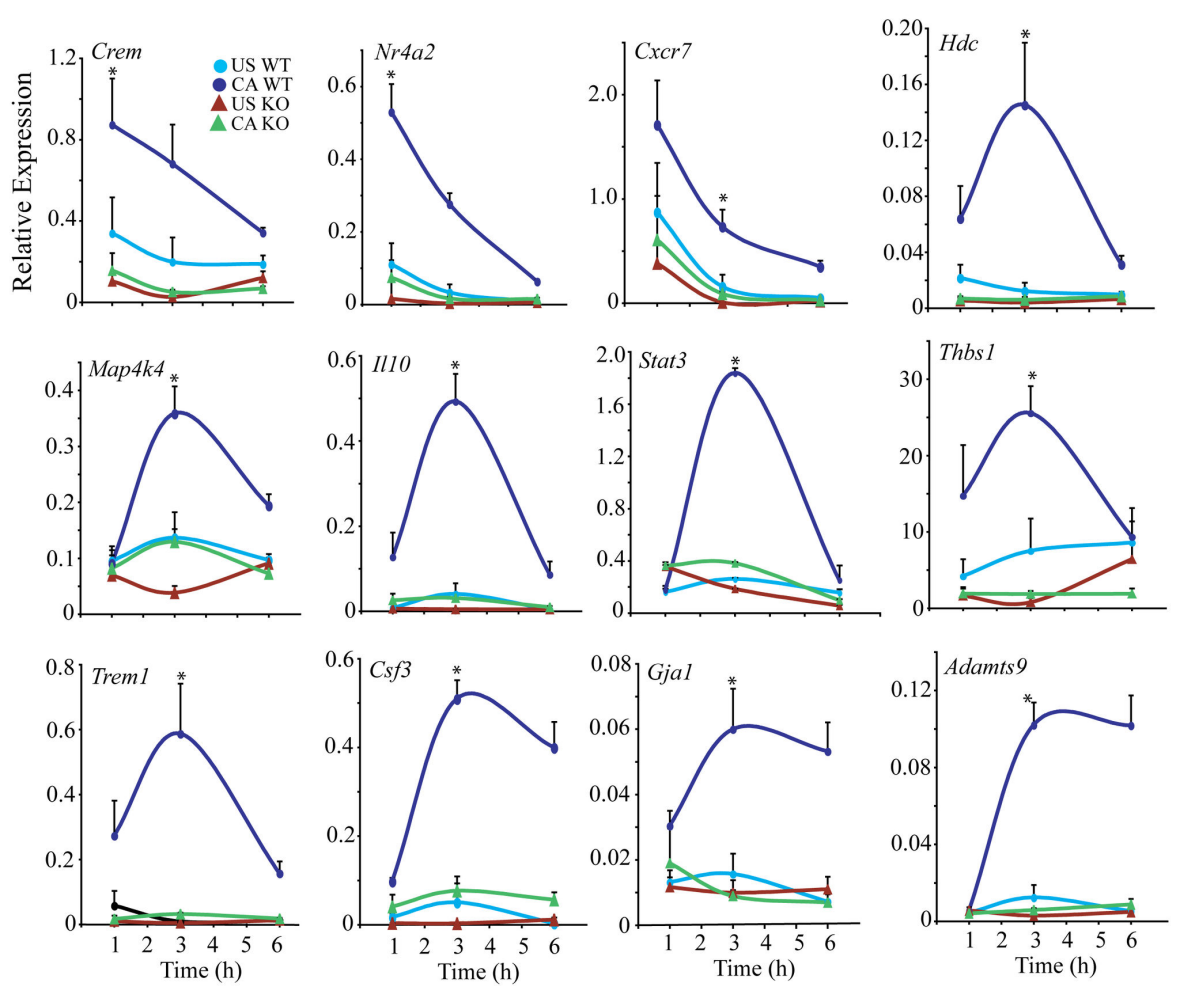
Time course of expression of genes expressed at higher levels in *C. albicans*-stimulated cPLA_2_α^+/+^ than cPLA_2_α^-/-^ RPM. cPLA_2_α^+/+^ (WT, circles) and cPLA_2_α^-/-^ (KO, triangles) RPM were incubated with (CA) or without (US) *C. albicans* for the indicated times. RNA was isolated and gene expression determined by real-time PCR using the RT^2^ Profiler PCR Array System (SA Bioscience) as described in Experimental Design. The data were normalized to the housekeeping genes *Gapdh* and *Hprt*. The results are the average of 3 experiments ±S.E. Gene expression in *C. albicans* infected WT at 3 h was compared to *C. albicans* infected KO at 3 h to determine significance (*p<0.05).

The microarray results were corroborated by real-time PCR analysis for several representative genes expressed at higher levels in cPLA_2_α^+/+^ than cPLA_2_α^-/-^ RPM (Figure 6). When analyzed 1-6 h after *C. albicans* addition, several early response genes (*Crem, Nr4a2, Cxcr7*) showed highest expression in cPLA_2_α^+/+^ RPM at 1 h. The early induction of the transcriptional regulators *Crem* and *Nr4a2* due to cPLA_2_α activation suggests that they play a role in regulating gene expression to increases in cAMP. The expression of most genes peaked 3 h after *C. albicans* addition with some decreasing to near baseline by 6 h (*Hdc*, Map*4k4*, *Il10*, *Stat3*, *Thbs1*, *Trem1*) while others remained elevated (*Csf3*, *Adamts9*, *Gja1*).

### Role of the IP receptor and PKA in regulating gene expression

**Figure 7:**
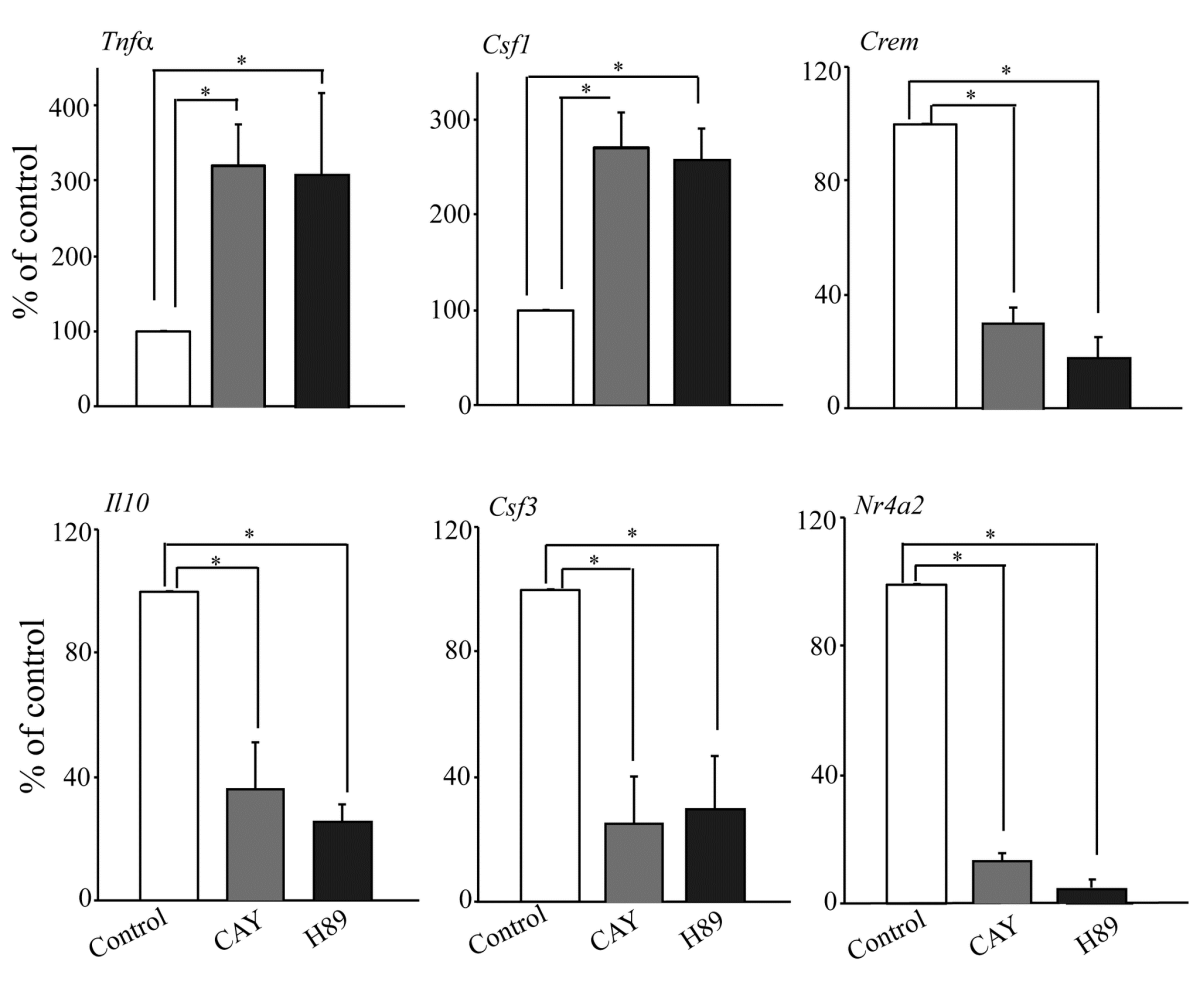
Effect of IP receptor antagonist and PKA inhibitor on gene expression. cPLA_2_α^+/+^ RPM were incubated with the IP receptor antagonist CAY10441 (1 µM) (light gray bars) and the PKA inhibitor H89 (10 µM) (black bars) for 30 min followed by stimulation with *C. albicans* for 3 h. RNA was isolated and gene expression determined by real-time PCR. Gene expression values are presented as the % of control values (set at 100%), which is *C. albicans*-stimulated RPM not treated with CAY10441 or H89. The results are the average of 3 experiments ±S.E. (*p<0.05).

We investigated the role of prostacyclin production (the prostanoid produced at the highest level in RPM) and PKA, the downstream mediator of cAMP, in regulating gene expression by treating cPLA_2_α^+/+^ RPM with the IP receptor antagonist CAY10441 and the PKA inhibitor H89 (Figure 7). Representative genes expressed at lower levels in *C. albicans*-stimulated cPLA_2_α^+/+^ than cPLA_2_α^-/-^ RPM (*Tnfα* and *Csf1*) were enhanced by blocking the action of PGI_2_ and inhibiting PKA. In contrast, genes expressed at higher levels in cPLA_2_α^+/+^ than cPLA_2_α^-/-^ RPM (*Crem*, *Il10*, *Csf3*, *Nr4a2*) were suppressed by the IP receptor antagonist and by the PKA inhibitor. The results suggest that cPLA_2_α-mediated prostaglandin production promotes an autocrine loop to increase cAMP and PKA activation for regulating expression of these genes.

## Discussion

In this study we describe the changes in gene expression that occur in RPM during infection with *C. albicans*, and how gene expression is influenced by the activation of cPLA_2_α and endogenously produced lipid mediators. Resident tissue macrophages are sentinel cells that are important in first sensing and responding to microbial invasion. Therefore our study investigates how cPLA_2_α activation modulates macrophage responses during the initial stages of infection to affect the balance of host defense and inflammation. The production of eicosanoids in RPM is dependent on cPLA_2_α activation to provide arachidonic acid [12,14]. They are released within minutes of activation by *C. albicans* to rapidly engage eicosanoid receptors for regulating transcriptional responses. Although there have been a number of studies investigating the effect of adding exogenous eicosanoids to cells, by comparing cPLA_2_α^+/+^ and cPLA_2_α^-/-^ RPM we are probing the primary mechanism for production of eicosanoids in macrophages at levels expected to occur locally in tissues in response to microbial infection. Our analysis provides global insight into the extensive changes in gene expression that are initiated by activation of cPLA_2_α and endogenously produced eicosanoids in resident tissue macrophages early in response to microbial infection.

The recognition of *C. albicans* by macrophages is complex since the fungal cell wall contains several chemical components that differentially engage a number of receptors including a variety of TLRs and lectins [86]. These receptors promote unique signaling pathways that preferentially induce distinct cellular responses. In RPM *C. albicans* triggers rapid activation of mitogen-activated protein kinases and calcium mobilization necessary for cPLA_2_α activation through dectin-1, dectin-2 and MyD88 pathways [13,14]. The results of this study suggest that the differential expression of many genes observed in cPLA_2_α^+/+^ and cPLA_2_α^-/-^ RPM is due to an autocrine loop involving cPLA_2_α, prostaglandins and increased cAMP production, which is significantly higher in *C. albicans*-stimulated cPLA_2_α^+/+^ than *C. albicans*-stimulated cPLA_2_α^-/-^ RPM. This is illustrated by results showing that TNFα production is suppressed by prostaglandins through increases in cAMP. Expression of TNFα occurs in part through dectin-1 and TLR4 in RPM that activate NF-κB and transcription [86]. In RPM the rapid production of prostanoids, particularly PGI_2_ that acts through the IP receptor, increases cAMP and PKA activation that suppresses transcription by mechanisms that are not fully understood. In addition to TNFα we observed differential expression of several genes previously reported to be regulated by prostaglandins and increases in cAMP in a variety of cell types. These include *Ccl5*, *Socs3*, *Il10*, *Gja1*, *Crem*, *Thbd*, *Abca1*, *Csf3*, *Trem1* [33,69,73,87–93]. Similar to our results in *C. albicans*-stimulated RPM, an autocrine loop pathway involving cPLA_2_α, prostacyclin and cAMP has been shown to enhance expression of *Areg*, *Ereg* and *Fst*, Cre-dependent genes involved in vascular remodeling and angiogenesis [94]. This autocrine loop involving prostaglandins and cAMP is triggered in many cell types in response to a variety of agonists indicating that it is an important, widely used pathway for regulating gene expression.

The rapid increase in cAMP that occurs in *C. albicans*-stimulated cPLA_2_α^+/+^ RPM is consistent with functional coupling of cPLA_2_α activation and metabolism of arachidonic acid to prostanoids by constitutively expressed COX1 since the response occurs before the expression of COX2. A role for COX1 in mediating prostaglandin production in LPS-stimulated RPM has previously been reported [34]. COX1 provides prostaglandins that regulate normal physiological processes and can regulate the early phases of inflammation [17]. RPM express the EP2, EP4 and IP receptors that mediate increases in cAMP, and our results show that EP2 or IP receptor agonists suppress TNFα production. It is likely that PGI_2_ and PGE_2_ both contribute to the regulation of transcription through increases in cAMP. However, PGI_2_ is produced at higher amounts than PGE_2_ during the first 15-30 min after activation by *C. albicans*. We were not successful in testing the EP2 receptor antagonist due to adverse effects on RPM. Although not addressed in this study, other eicosanoids such as LTC_4_ and arachidonic acid itself released by RPM in response to *C. albicans* could also influence macrophage activation. Arachidonic acid has been shown to suppress the expression of the complement receptor immunoglobulin (CRIg) during maturation of human monocytes to macrophages resulting in a decrease in the phagocytosis of opsonized *C. albicans* [95]. LTC4 could act through the CYSLT1 and CYSLT2 receptors expressed on RPM. For example these receptors promote calcium mobilization that may influence transcriptional responses due to cross-talk with cAMP signaling, and by potentiating cPLA_2_α activation [96]. Leukotrienes have been shown to promote uptake of *C. albicans* by alveolar macrophages and to enhance fungicidal activity [97]. It is possible that leukotrienes contribute to the enhanced *C. albicans* killing we observed in cPLA_2_α^+/+^ RPM compared to cPLA_2_α^-/-^ RPM.

Microbial pathogens engage PRRs on macrophages that induce extensive effects on gene expression as we observed in *C. albicans*-stimulated RPM [39]. A characteristic of the "common host response" is increased expression of a large number of pro-inflammatory cytokines and chemokines that is important for the recruitment and activation of myeloid cells during infection [39]. Pro-inflammatory host defense responses are balanced by the activation of negative feedback loops that are important in dampening inflammation and potential tissue damage [21]. Our data suggest that cPLA_2_α activation and lipid mediator production represents one of the negative feedback loops since cPLA_2_α^+/+^ RPM exhibit lower expression of select pro-inflammatory genes such as *Tnfα*, *Csf1*, *Ccl5*, *Cd40*, *Cx3cl1*, *Edn*, *Ifnγ* and several IFNγ regulated GTP binding proteins, and higher expression of anti-inflammatory genes such as *Il10*, *Socs3*, *Stat3*, *Fst*, *Thbd*, *Thsp1*, *Calca* and *CxCr7* than cPLA_2_α^-/-^ RPM. Historically there has been an emphasis on the role of prostaglandins in mediating the cardinal signs of inflammation that is supported by the clinical effects of non-steroidal anti-inflammatory drugs. However, prostaglandins play an important role in suppressing inflammation and immune responses by acting through prostanoid receptors that increase cAMP resulting in PKA activation as supported by our results [18]. This pathway has immunosuppressive effects by inhibiting the differentiation of antigen presenting cells, lymphocyte activation and production of Th1 cytokines.

Our results show that the activation of cPLA_2_α and coupling to COX1 is an early response to *C. albicans* infection of RPM that can regulate the amplitude and timing of inflammation and host defense mechanisms as exemplified by the decrease in expression of *Tnfα* and increase of *Il10*. ERK activation and calcium mobilization are the signaling cascades activated by PRRs that are important for promoting IL10 production [69,98]. These are the signals required for optimal cPLA_2_α activation and eicosanoid production [99]. This cytokine signature is also a characteristic of resolution phase macrophages that contribute to restoration of normal tissue function by dampening inflammatory signals and the clearance of apoptotic neutrophils [100,101]. Resolution phase macrophages are characterized by the expression of COX2, decreased TNFα and increased IL10 production controlled by cAMP production. Prostaglandins and increases in cAMP contribute to the resolution phase by enhancing the ability of macrophages to phagocytose apoptotic neutrophils [102,103]. Activated and apoptotic neutrophils produce lyso-phosphatidylserine that acts through the macrophage G2A receptor to trigger an autocrine loop involving cPLA_2_α activation, PGE_2_ production, EP2 receptor-dependent increases in cAMP and PKA activation to enhance efferocytosis [102,104]. Therefore cPLA_2_α activation and prostaglandin production play a role in balancing host defense responses and the extent of inflammation in both the initiation and resolution phases of infection.

The results also indicate that cPLA_2_α-mediated prostaglandin production enhances the expression of certain pro-inflammatory genes, such as *Csf3*, that are important for host defense against *C. albicans* infection by promoting neutrophil function [40,41]. Prostaglandins also contribute to Candidiasis protection by promoting the Th17 response [105,106]. IL17 regulates neutrophil recruitment and is important for host defense to mucocutaneous Candidiasis [107–110]. However if pro-inflammatory responses go unchecked prostaglandins contribute to chronic inflammation that is characteristic of cancer, and vascular and autoimmune diseases [111]. The ability of prostaglandins to promote the development of Th17 differentiation and production of IL17 contributes to chronic inflammation associated with autoimmune diseases [111,112]. COX2 is overexpressed in cancers and prostaglandins promote cancer development by regulating angiogenesis, cell migration, adhesion and invasiveness in part through promoting receptor specific increases in cAMP [57,58]. Several of the genes that are differentially expressed in cPLA_2_α^+/+^ RPM and cPLA_2_α^-/-^ RPM (i.e. *Gdf15*, *Eph2*, *Ereg*, *Areg*, *Lepr*, *Nr4a2*, *Runx2*, *Mmp13*, *CxCr7*, *Pdpn*, *Prox1*) are positively or negatively regulated in cancers compared to normal tissue as a result of prostaglandins [59–63,113]. Therefore, eicosanoids have complex biological effects depending on the tissue context, the specific receptors expressed on cells in the local environment and the timing of their production contributing to both anti- and pro-inflammation responses. Results from this study support an important role for cPLA_2_α activation early in response to microbial infection in resident tissue macrophages that helps to balance the expression of genes important for host defense and genes that contribute to inflammation.

## Supporting Information

Table S1 S1A and S1B Genes (A) increased and (B) decreased by *C. albicans* in wild type RPM.
(DOC)

Table S2 S2A and S2B Genes expressed at (A) lower and (B) higher levels in *C. albicans*-stimulated cPLA_2_α^+/+^ than cPLA_2_α^-/-^ RPM.
(DOC)
