# Supplementary material for: Cytosolic Phospholipase A2α and Eicosanoids Regulate Expression of Genes in Macrophages Involved in Host Defense and Inflammation
Source: PLoS One. 2013 Jul 25;8(7):e69002. doi: 10.1371/journal.pone.0069002 (PMC3742295; doi:10.1371/journal.pone.0069002)
Supplement: Table S1 — S1A and S1B Genes (A) increased and (B) decreased by C. albicans in wild type RPM. (DOC) [file pone.0069002.s001.doc]

**Table S1A**. **Genes increased by *C. albicans* in wild type RPM**

The expression values (Exp.Val) of genes increased (427 genes, ≥4.0-fold, p<0.05, n=3) in wild type (WT) RPM

stimulated with *C. albicans* (CA) for 3 h compared to unstimulated (US) RPM are shown.

|  |  | **WT+CA/WT+US** | | **WT+CA** | | **WT+US** | |
| --- | --- | --- | --- | --- | --- | --- | --- |
| **GeneName** | **ID** | **Fold↑** | **p-value** | **Exp.Val** | **±SD** | **Exp.Val** | **±SD** |
| Trib3 | 228775 | 4.64 | 0.000040 | 796.83 | 280.85 | 227.50 | 105.26 |
| Lfng | 16848 | 4.04 | 0.000054 | 683.67 | 113.58 | 221.67 | 57.57 |
| Gm15247 | 100379606 | 92.16 | 0.000154 | 7515.50 | 2937.11 | 116.50 | 79.53 |
| Rai14 | 75646 | 5.20 | 0.000200 | 137.67 | 51.61 | 40.00 | 30.05 |
| Hspa4l | 18415 | 5.83 | 0.000209 | 1859.00 | 384.31 | 442.50 | 162.35 |
| Sema6d | 214968 | 4.24 | 0.000215 | 93.67 | 17.67 | 33.83 | 14.27 |
| Clic4 | 29876 | 6.24 | 0.000245 | 10413.50 | 933.14 | 2421.17 | 910.19 |
| St6galnac4 | 20448 | 5.38 | 0.000324 | 320.33 | 42.62 | 97.67 | 35.70 |
| Gzmb | 14939 | 7.23 | 0.000338 | 65.67 | 26.80 | 13.33 | 7.77 |
| Phlda1 | 21664 | 27.21 | 0.000350 | 7770.00 | 4842.30 | 356.33 | 216.10 |
| Mthfd1l | 270685 | 4.97 | 0.000364 | 1472.33 | 326.75 | 469.83 | 268.09 |
| Mid1 | 17318 | 17.61 | 0.000382 | 260.67 | 40.85 | 17.00 | 18.87 |
| Pabpc4 | 230721 | 4.87 | 0.000387 | 60.17 | 8.13 | 19.00 | 8.23 |
| Nr4a3 | 18124 | 18.22 | 0.000396 | 44.00 | 13.08 | 1.17 | 2.02 |
| Ddit4 | 74747 | 21.53 | 0.000400 | 13613.17 | 2734.69 | 827.17 | 48.47 |
| Gm15247 | 100379606 | 71.14 | 0.000421 | 8599.50 | 3385.62 | 164.83 | 115.58 |
| Hk1 | 15275 | 7.05 | 0.000428 | 21481.97 | 1748.70 | 4181.33 | 1394.06 |
| Mt2 | 17750 | 20.89 | 0.000435 | 47592.80 | 7528.85 | 3277.17 | 796.77 |
| Jmjd6 | 107817 | 6.39 | 0.000572 | 5016.67 | 1051.42 | 1060.83 | 420.96 |
| Gpr35 | 64095 | 17.02 | 0.000579 | 965.17 | 114.00 | 76.67 | 30.92 |
| Id1 | 15901 | 17.65 | 0.000586 | 237.50 | 112.30 | 16.00 | 16.04 |
| Nfkb1 | 18033 | 6.09 | 0.000653 | 7097.83 | 470.71 | 1638.00 | 475.36 |
| Akap2 | 11641 | 4.74 | 0.000720 | 591.67 | 207.78 | 157.00 | 41.68 |
| Rapgef2 | 76089 | 7.90 | 0.000727 | 3450.33 | 171.65 | 605.83 | 198.06 |
| Ankrd37 | 654824 | 16.69 | 0.000733 | 3569.67 | 2022.39 | 264.67 | 134.65 |
| Rab11fip1 | 75767 | 16.74 | 0.000760 | 3420.33 | 466.22 | 289.50 | 93.90 |
| Pfkfb3 | 170768 | 4.58 | 0.000764 | 101.00 | 13.08 | 37.17 | 18.50 |
| Gfpt1 | 14583 | 21.15 | 0.000778 | 254.50 | 16.93 | 17.50 | 12.22 |
| C87414 | 381654 | 16.65 | 0.000795 | 295.67 | 59.78 | 21.83 | 11.03 |
| Hspa4l | 18415 | 5.70 | 0.000805 | 656.00 | 132.60 | 186.50 | 81.43 |
| Higd1a | 56295 | 4.49 | 0.000869 | 4238.33 | 303.44 | 1279.83 | 348.55 |
| Cxcl2 | 20310 | 22.74 | 0.000882 | 40915.47 | 9946.60 | 2424.83 | 113.35 |
| Ero1l | 50527 | 9.35 | 0.000966 | 569.00 | 166.62 | 81.33 | 30.59 |
| Mxd1 | 17119 | 4.04 | 0.000995 | 3184.67 | 412.43 | 1149.67 | 463.74 |
| Rsbn1 | 229675 | 6.33 | 0.001013 | 51.33 | 5.51 | 10.50 | 6.73 |
| Spty2d1 | 101685 | 4.16 | 0.001072 | 700.00 | 65.97 | 232.50 | 74.04 |
| Ak4 | 11639 | 8.77 | 0.001099 | 274.17 | 176.53 | 43.83 | 43.43 |
| Kdelr3 | 105785 | 4.25 | 0.001120 | 82.50 | 46.30 | 25.00 | 18.43 |
| Traf5 | 22033 | 4.03 | 0.001124 | 1638.33 | 389.50 | 550.33 | 103.82 |
| Efnb2 | 13642 | 10.08 | 0.001128 | 6908.17 | 437.34 | 1131.83 | 401.53 |
| F3 | 14066 | 191.39 | 0.001129 | 22426.90 | 7158.96 | 150.50 | 57.73 |
| F3 | 14066 | 182.42 | 0.001141 | 24826.33 | 9384.67 | 177.17 | 78.11 |
| Psat1 | 107272 | 6.34 | 0.001143 | 655.67 | 80.60 | 162.83 | 68.57 |
| Cxcl1 | 14825 | 25.29 | 0.001152 | 64350.57 | 17568.39 | 3268.33 | 815.51 |
| Slc2a1 | 20525 | 22.17 | 0.001159 | 44019.00 | 2054.50 | 2922.17 | 1027.10 |
| Rlf | 109263 | 4.08 | 0.001201 | 2181.67 | 66.81 | 746.67 | 194.48 |
| Rcan1 | 54720 | 19.53 | 0.001248 | 5851.67 | 1505.61 | 492.67 | 235.11 |
| Uck2 | 80914 | 13.65 | 0.001267 | 670.50 | 318.73 | 78.33 | 43.43 |
| Alcam | 11658 | 5.30 | 0.001271 | 1559.83 | 377.78 | 410.83 | 218.19 |
| Ptgs2 | 19225 | 216.95 | 0.001283 | 11243.00 | 2938.27 | 67.33 | 13.80 |
| Chac1 | 69065 | 13.64 | 0.001450 | 5291.67 | 1008.59 | 689.33 | 310.88 |
| Serpina3f | 238393 | 4.71 | 0.001491 | 11.00 | 7.05 | 1.50 | 3.77 |
| Ptgs2 | 19225 | 193.54 | 0.001512 | 8668.17 | 2840.67 | 64.33 | 11.03 |
| Adora2b | 11541 | 15.43 | 0.001628 | 1697.00 | 460.39 | 140.83 | 48.54 |
| Fzd8 | 14370 | 5.84 | 0.001642 | 523.83 | 190.63 | 133.00 | 100.37 |
| Marcksl1 | 17357 | 7.25 | 0.001645 | 13731.17 | 1616.66 | 2475.50 | 403.83 |
| Basp1 | 70350 | 10.68 | 0.001650 | 1355.00 | 343.98 | 179.83 | 80.80 |
| Itgb3 | 16416 | 5.41 | 0.001671 | 201.50 | 50.94 | 45.17 | 9.39 |
| Il17ra | 16172 | 5.69 | 0.001707 | 2785.17 | 293.21 | 708.33 | 158.67 |
| Plek | 56193 | 5.42 | 0.001722 | 26923.60 | 4306.22 | 7232.33 | 2855.72 |
| Pbx1 | 18514 | 4.14 | 0.001749 | 70.00 | 29.00 | 23.00 | 16.18 |
| Ccno | 218630 | 10.77 | 0.001782 | 2432.50 | 1060.73 | 291.33 | 102.21 |
| Zfand5 | 22682 | 4.33 | 0.001816 | 3053.00 | 395.03 | 1001.83 | 291.75 |
| Il1rn | 16181 | 55.35 | 0.001821 | 1543.67 | 88.26 | 40.83 | 22.05 |
| Irf4 | 16364 | 6.63 | 0.001840 | 189.33 | 28.44 | 38.83 | 21.80 |
| Slc3a2 | 17254 | 4.63 | 0.001857 | 40670.60 | 1600.80 | 12069.67 | 2638.81 |
| Hk1 | 15275 | 4.85 | 0.001955 | 1136.67 | 241.59 | 323.67 | 144.79 |
| Piga | 18700 | 5.03 | 0.001976 | 836.17 | 109.25 | 235.50 | 65.84 |
| Slc20a1 | 20515 | 9.76 | 0.001982 | 9300.17 | 1070.39 | 1696.33 | 825.09 |
| St6galnac4 | 20448 | 6.01 | 0.001995 | 573.00 | 37.81 | 124.50 | 46.29 |
| Trib3 | 228775 | 7.19 | 0.002028 | 2291.50 | 482.55 | 478.33 | 239.65 |
| Hspa4l | 18415 | 5.78 | 0.002103 | 3546.50 | 304.59 | 809.67 | 225.54 |
| Pla2g4a | 18783 | 5.64 | 0.002187 | 1203.33 | 178.69 | 298.83 | 39.07 |
| Egr1 | 13653 | 59.93 | 0.002221 | 5759.17 | 2149.58 | 141.00 | 48.99 |
| Ppp1r3g | 76487 | 370.35 | 0.002230 | 584.17 | 112.29 | -1.17 | 3.75 |
| Slc7a1 | 11987 | 5.06 | 0.002245 | 608.17 | 77.66 | 162.50 | 64.17 |
| Gfpt1 | 14583 | 23.69 | 0.002281 | 579.17 | 25.26 | 35.83 | 13.18 |
| Plek | 56193 | 5.23 | 0.002290 | 15735.00 | 1940.21 | 4388.50 | 1839.33 |
| Marcksl1 | 17357 | 7.04 | 0.002400 | 21220.57 | 1453.44 | 3941.17 | 422.10 |
| Sphk1 | 20698 | 65.30 | 0.002408 | 4013.17 | 354.25 | 96.00 | 74.38 |
| Il17ra | 16172 | 6.09 | 0.002417 | 2871.67 | 253.17 | 644.33 | 72.85 |
| Osm | 18413 | 13.63 | 0.002451 | 841.50 | 322.89 | 81.50 | 3.91 |
| Fam107b | 66540 | 6.17 | 0.002517 | 51.67 | 1.61 | 8.00 | 5.41 |
| Flrt3 | 71436 | 33.20 | 0.002524 | 5553.83 | 906.89 | 284.83 | 126.29 |
| Kctd12 | 239217 | 6.11 | 0.002537 | 2616.00 | 633.06 | 560.50 | 203.91 |
| Scx | 20289 | 4.61 | 0.002541 | 351.50 | 92.69 | 100.33 | 57.49 |
| Fosl1 | 14283 | 23.44 | 0.002544 | 1162.67 | 129.07 | 70.50 | 37.95 |
| Asns | 27053 | 6.23 | 0.002567 | 11771.50 | 576.29 | 3159.33 | 972.86 |
| Rlf | 109263 | 4.21 | 0.002587 | 653.67 | 143.49 | 222.67 | 103.88 |
| Slco2b1 | 101488 | 16.92 | 0.002590 | 34.33 | 6.29 | 0.33 | 5.13 |
| Odc1 | 18263 | 7.92 | 0.002614 | 411.00 | 38.97 | 90.33 | 41.79 |
| Isg15 | 100038882 | 13.55 | 0.002616 | 2006.17 | 866.89 | 174.50 | 67.40 |
| Mcm10 | 70024 | 5.30 | 0.002689 | 204.17 | 72.04 | 49.67 | 34.12 |
| Slc7a5 | 20539 | 5.05 | 0.002717 | 1221.00 | 248.61 | 323.00 | 144.90 |
| Cd244 | 18106 | 4.37 | 0.002776 | 1369.00 | 358.95 | 463.00 | 195.19 |
| Fam78b | 226610 | 4.86 | 0.002779 | 234.17 | 53.75 | 60.50 | 27.99 |
| Il23a | 83430 | 18.75 | 0.002805 | 59.00 | 20.93 | 2.83 | 1.61 |
| Rai14 | 75646 | 8.71 | 0.002811 | 968.67 | 36.68 | 153.33 | 64.74 |
| Proz | 66901 | 6.71 | 0.002823 | 285.67 | 127.15 | 57.83 | 30.75 |
| Loxl2 | 94352 | 9.92 | 0.002827 | 1268.17 | 218.84 | 166.00 | 50.48 |
| Eaf1 | 74427 | 4.42 | 0.002868 | 2064.83 | 326.63 | 832.67 | 508.04 |
| Vegfa | 22339 | 45.03 | 0.002912 | 6619.17 | 2050.28 | 206.67 | 82.98 |
| Vegfa | 22339 | 45.03 | 0.002912 | 5722.33 | 1617.15 | 184.67 | 61.85 |
| Vegfa | 22339 | 45.03 | 0.002912 | 6586.67 | 2024.96 | 175.83 | 75.76 |
| Nr4a2 | 18227 | 80.95 | 0.002955 | 8312.67 | 4057.49 | 123.17 | 15.28 |
| Pbx1 | 18514 | 4.47 | 0.003016 | 165.17 | 17.78 | 44.17 | 19.09 |
| Bmp6 | 12161 | 4.49 | 0.003097 | 104.33 | 79.39 | 30.67 | 31.66 |
| Serpine1 | 18787 | 68.91 | 0.003139 | 6292.00 | 867.80 | 118.50 | 13.43 |
| Il1rn | 16181 | 51.46 | 0.003155 | 34223.13 | 4022.02 | 849.00 | 280.32 |
| Rai14 | 75646 | 6.69 | 0.003178 | 218.67 | 22.90 | 43.83 | 22.51 |
| Phldb1 | 102693 | 9.65 | 0.003201 | 4250.00 | 475.82 | 583.00 | 107.72 |
| Pabpc4 | 230721 | 5.14 | 0.003253 | 3395.17 | 125.12 | 930.83 | 336.74 |
| Mthfd2 | 17768 | 4.33 | 0.003264 | 1172.17 | 335.17 | 332.50 | 139.19 |
| Smox | 228608 | 6.75 | 0.003294 | 7416.50 | 1378.58 | 1536.83 | 572.15 |
| Csrnp1 | 215418 | 4.90 | 0.003350 | 93.67 | 25.11 | 22.50 | 3.97 |
| Nr4a1 | 15370 | 17.92 | 0.003380 | 43841.73 | 21077.82 | 3285.67 | 1477.06 |
| Pfkp | 56421 | 6.28 | 0.003385 | 561.83 | 88.21 | 119.33 | 23.53 |
| Loxl2 | 94352 | 7.23 | 0.003403 | 272.00 | 16.37 | 50.17 | 19.02 |
| Edn1 | 13614 | 53.33 | 0.003408 | 141.33 | 55.72 | 2.33 | 7.64 |
| Socs3 | 12702 | 16.51 | 0.003433 | 71520.60 | 19416.89 | 6126.33 | 1915.93 |
| Ccl7 | 20306 | 95.05 | 0.003462 | 1306.33 | 961.62 | 15.17 | 8.75 |
| Zfp36 | 22695 | 7.96 | 0.003523 | 7050.83 | 2515.57 | 1160.83 | 70.49 |
| Hk2 | 15277 | 18.22 | 0.003523 | 24470.60 | 2326.19 | 1933.50 | 903.88 |
| Thap2 | 66816 | 4.91 | 0.003546 | 262.33 | 33.65 | 96.83 | 48.76 |
| Sdc4 | 20971 | 4.31 | 0.003564 | 1011.50 | 109.78 | 309.83 | 90.62 |
| Cish | 12700 | 32.65 | 0.003571 | 4294.33 | 1090.34 | 213.67 | 92.52 |
| Csf3 | 12985 | 643.38 | 0.003589 | 10887.83 | 5124.02 | 21.00 | 8.35 |
| Tes | 21753 | 4.02 | 0.003634 | 174.33 | 23.18 | 59.83 | 26.60 |
| Sesn2 | 230784 | 5.75 | 0.003640 | 8948.33 | 742.34 | 2040.67 | 327.88 |
| Olr1 | 108078 | 8.48 | 0.003650 | 4911.00 | 1690.10 | 774.67 | 312.52 |
| Acp5 | 11433 | 4.95 | 0.003688 | 3744.17 | 1342.87 | 982.50 | 133.06 |
| Slc16a3 | 80879 | 13.12 | 0.003705 | 40221.87 | 4160.79 | 4738.67 | 1224.35 |
| Osm | 18413 | 13.54 | 0.003708 | 3729.33 | 1354.63 | 340.33 | 83.06 |
| Rab11fip1 | 75767 | 28.36 | 0.003724 | 4304.33 | 1453.25 | 214.17 | 51.25 |
| Unc13a | 382018 | 6.77 | 0.003725 | 192.00 | 32.36 | 35.67 | 19.37 |
| Gja1 | 14609 | 50.69 | 0.003726 | 104.83 | 19.28 | -0.50 | 5.89 |
| Gja1 | 14609 | 50.69 | 0.003726 | 106.83 | 33.13 | 0.50 | 6.87 |
| Tmtc2 | 278279 | 14.55 | 0.003742 | 185.67 | 34.32 | 16.83 | 13.73 |
| Gfpt1 | 14583 | 9.68 | 0.003826 | 3899.67 | 734.29 | 562.50 | 212.59 |
| Mmp13 | 17386 | 6.99 | 0.003863 | 3499.67 | 1047.79 | 714.67 | 398.01 |
| Nfkbid | 243910 | 20.70 | 0.003887 | 2392.50 | 629.83 | 151.17 | 30.73 |
| Piga | 18700 | 15.78 | 0.003896 | 735.67 | 127.21 | 63.33 | 31.13 |
| Oaf | 102644 | 10.36 | 0.003912 | 357.50 | 50.60 | 50.33 | 16.93 |
| Rsbn1 | 229675 | 4.21 | 0.003923 | 944.83 | 115.10 | 294.50 | 108.68 |
| Ppp1r3b | 244416 | 4.60 | 0.003959 | 101.83 | 31.37 | 32.17 | 15.28 |
| Ifrd1 | 15982 | 6.19 | 0.003964 | 48.17 | 17.96 | 9.33 | 7.65 |
| Ndrg1 | 17988 | 11.70 | 0.003989 | 9833.33 | 4475.17 | 1444.00 | 1277.35 |
| Cxcl3 | 330122 | 24.79 | 0.003990 | 9811.17 | 5188.43 | 560.67 | 409.96 |
| Fam110c | 104943 | 8.27 | 0.004014 | 164.17 | 34.62 | 26.83 | 14.37 |
| Gpr35 | 64095 | 36.41 | 0.004018 | 685.33 | 75.66 | 23.17 | 7.85 |
| Fam107b | 66540 | 7.39 | 0.004022 | 205.50 | 21.40 | 30.83 | 12.19 |
| Ptges | 64292 | 5.45 | 0.004041 | 8763.33 | 1147.34 | 2307.33 | 915.69 |
| Nktr | 18087 | 6.30 | 0.004050 | 2471.50 | 215.28 | 577.83 | 268.47 |
| Dnahc2 | 327954 | 13.61 | 0.004088 | 483.17 | 257.53 | 49.67 | 23.03 |
| Ccl4 | 20303 | 58.45 | 0.004162 | 126787.87 | 29071.80 | 3343.67 | 1764.13 |
| Plaur | 18793 | 8.48 | 0.004207 | 23759.57 | 5665.23 | 4390.33 | 1233.02 |
| Sphk1 | 20698 | 91.87 | 0.004208 | 31479.03 | 3943.98 | 517.00 | 299.81 |
| Adm | 11535 | 9.75 | 0.004249 | 750.67 | 526.44 | 93.50 | 48.62 |
| Rab8b | 235442 | 6.45 | 0.004273 | 1131.17 | 144.75 | 250.33 | 88.82 |
| Hk1 | 15275 | 6.78 | 0.004286 | 364.83 | 137.22 | 70.67 | 24.54 |
| Ralgds | 19730 | 6.08 | 0.004317 | 27979.90 | 3261.98 | 6552.33 | 3093.78 |
| Fem1c | 240263 | 8.10 | 0.004318 | 4528.50 | 949.62 | 775.50 | 218.67 |
| Id2 | 15902 | 11.88 | 0.004350 | 14381.67 | 5789.13 | 1646.00 | 871.46 |
| Maff | 17133 | 84.88 | 0.004371 | 28479.83 | 1462.90 | 501.00 | 324.66 |
| Fabp3 | 14077 | 4.78 | 0.004434 | 8446.00 | 882.80 | 2529.17 | 883.28 |
| Cd83 | 12522 | 22.00 | 0.004469 | 8905.83 | 2248.18 | 520.17 | 113.50 |
| Emp1 | 13730 | 6.93 | 0.004471 | 9870.33 | 1121.61 | 2556.17 | 1586.82 |
| Asb4 | 65255 | 8.53 | 0.004511 | 2212.17 | 475.56 | 335.67 | 41.71 |
| Rab8b | 235442 | 7.51 | 0.004560 | 1734.00 | 265.00 | 317.50 | 110.06 |
| Pcf11 | 74737 | 5.34 | 0.004590 | 416.83 | 69.32 | 113.67 | 55.43 |
| Apol8 | 239552 | 18.70 | 0.004602 | 41.67 | 24.11 | 0.67 | 5.62 |
| Bhlhe40 | 20893 | 16.20 | 0.004614 | 6519.50 | 965.54 | 642.67 | 421.71 |
| Itgav | 16410 | 4.04 | 0.004669 | 2399.17 | 356.34 | 837.00 | 313.14 |
| Ccl3 | 20302 | 17.95 | 0.004724 | 63270.77 | 3003.64 | 4697.17 | 2251.71 |
| Ttc39b | 69863 | 4.03 | 0.004780 | 114.50 | 11.69 | 40.00 | 20.80 |
| Plcl1 | 227120 | 5.62 | 0.004787 | 58.17 | 13.00 | 11.50 | 4.09 |
| Uck2 | 80914 | 9.80 | 0.004796 | 534.33 | 189.15 | 70.67 | 25.32 |
| Csrnp1 | 215418 | 47.93 | 0.004798 | 12526.83 | 4403.81 | 343.83 | 80.10 |
| BC031781 | 208768 | 4.45 | 0.004811 | 2610.33 | 181.27 | 798.00 | 243.58 |
| Mbd1 | 17190 | 7.35 | 0.004862 | 342.67 | 73.89 | 65.17 | 23.12 |
| Rgs1 | 50778 | 7.54 | 0.004980 | 10041.67 | 3313.93 | 1878.33 | 582.77 |
| Eif1a | 13664 | 4.35 | 0.004991 | 3583.50 | 539.12 | 1250.17 | 710.68 |
| Bcl2a1b | 12045 | 5.73 | 0.005030 | 30890.47 | 1155.95 | 8024.50 | 3858.25 |
| Malt1 | 240354 | 13.06 | 0.005035 | 4622.00 | 356.29 | 509.00 | 229.87 |
| Gch1 | 14528 | 5.10 | 0.005068 | 4527.17 | 378.15 | 1283.83 | 408.56 |
| Car13 | 71934 | 5.58 | 0.005144 | 2121.17 | 233.30 | 514.33 | 209.21 |
| Flrt3 | 71436 | 32.42 | 0.005176 | 1301.50 | 173.59 | 52.50 | 27.62 |
| Il1b | 16176 | 7.91 | 0.005189 | 89649.53 | 28434.53 | 15825.33 | 5624.57 |
| Id2 | 15902 | 9.18 | 0.005196 | 5588.33 | 2555.01 | 746.00 | 229.07 |
| Plaur | 18793 | 8.38 | 0.005343 | 12290.83 | 2968.33 | 2269.00 | 549.13 |
| Smcr8 | 237782 | 6.20 | 0.005361 | 269.33 | 50.81 | 59.50 | 35.93 |
| Trem1 | 58217 | 20.85 | 0.005394 | 1284.33 | 642.10 | 84.00 | 19.83 |
| Hdc | 15186 | 26.82 | 0.005444 | 4800.67 | 1679.87 | 223.00 | 22.52 |
| Zfp558-ps | 72230 | 10.38 | 0.005446 | 32.33 | 8.96 | 1.67 | 6.66 |
| Hspa4l | 18415 | 6.75 | 0.005479 | 223.17 | 26.83 | 41.33 | 30.29 |
| Kctd6 | 71393 | 4.32 | 0.005481 | 1251.83 | 41.33 | 414.50 | 133.64 |
| Srgn | 19073 | 4.11 | 0.005491 | 77445.27 | 5468.98 | 25308.13 | 6629.04 |
| Gabpb1 | 14391 | 5.08 | 0.005534 | 101.83 | 7.59 | 27.83 | 16.75 |
| Abca1 | 11303 | 4.17 | 0.005589 | 6842.67 | 732.83 | 2160.00 | 555.53 |
| Inhba | 16323 | 152.95 | 0.005656 | 11495.67 | 4476.63 | 114.17 | 70.26 |
| Malt1 | 240354 | 11.97 | 0.005704 | 1333.83 | 313.54 | 156.50 | 62.32 |
| Sla | 20491 | 6.04 | 0.005807 | 3008.67 | 219.89 | 724.17 | 202.90 |
| Areg | 11839 | 381.19 | 0.005957 | 1061.83 | 889.33 | 2.33 | 3.55 |
| Rcan1 | 54720 | 8.99 | 0.005961 | 386.83 | 98.57 | 62.33 | 40.81 |
| Vegfa | 22339 | 35.82 | 0.006080 | 4601.33 | 1533.55 | 154.50 | 49.27 |
| Htra4 | 330723 | 23.91 | 0.006093 | 244.67 | 53.18 | 10.33 | 7.52 |
| Rabgef1 | 56715 | 6.86 | 0.006111 | 2522.17 | 325.95 | 519.50 | 210.54 |
| Zfp36 | 22695 | 10.11 | 0.006154 | 1127.83 | 275.07 | 144.50 | 44.27 |
| Gm6742 | 627245 | 7.73 | 0.006165 | 2440.33 | 163.93 | 456.67 | 207.69 |
| Arhgap26 | 71302 | 4.34 | 0.006296 | 36.50 | 20.62 | 7.83 | 6.29 |
| Spty2d1 | 101685 | 5.37 | 0.006515 | 160.67 | 19.76 | 41.17 | 18.43 |
| Itgav | 16410 | 6.63 | 0.006553 | 166.83 | 42.26 | 35.67 | 26.00 |
| Ccrl2 | 54199 | 18.49 | 0.006577 | 26014.63 | 2329.15 | 1987.50 | 1003.73 |
| Zbtb7c | 207259 | 5.51 | 0.006666 | 54.33 | 31.66 | 13.17 | 13.70 |
| Blcap | 53619 | 14.66 | 0.006825 | 192.67 | 6.51 | 22.00 | 9.99 |
| Homer1 | 26556 | 7.76 | 0.006882 | 194.33 | 24.01 | 41.50 | 27.78 |
| Pgk1 | 18655 | 4.37 | 0.006935 | 29076.67 | 6039.75 | 11342.17 | 5490.31 |
| Csf2 | 12981 | 322.62 | 0.006952 | 1640.33 | 937.07 | 3.67 | 5.20 |
| Efnb2 | 13642 | 7.87 | 0.006993 | 349.83 | 29.76 | 63.33 | 27.95 |
| Bnip3 | 12176 | 10.98 | 0.006999 | 5821.67 | 2464.99 | 711.33 | 196.14 |
| Agpat9 | 231510 | 7.86 | 0.006999 | 2582.33 | 669.00 | 453.50 | 158.23 |
| Ptp4a1 | 19243 | 5.81 | 0.007010 | 4473.50 | 287.48 | 1188.33 | 617.54 |
| Pla2g4a | 18783 | 5.63 | 0.007182 | 2417.00 | 589.44 | 630.67 | 34.64 |
| Tspan33 | 232670 | 6.09 | 0.007185 | 160.83 | 63.62 | 38.50 | 26.02 |
| Il6 | 16193 | 108.98 | 0.007205 | 4682.67 | 3722.44 | 43.50 | 24.11 |
| Ccnf | 12449 | 8.83 | 0.007245 | 364.67 | 91.88 | 53.50 | 35.86 |
| St3gal1 | 20442 | 7.48 | 0.007250 | 5490.83 | 1681.95 | 945.17 | 128.83 |
| Vcam1 | 22329 | 14.12 | 0.007251 | 120.33 | 15.31 | 13.00 | 3.12 |
| Jarid2 | 16468 | 5.58 | 0.007253 | 182.67 | 28.86 | 48.67 | 20.40 |
| Uqcrq | 22272 | 4.12 | 0.007259 | 76.17 | 17.61 | 30.67 | 20.23 |
| Eml1 | 68519 | 13.62 | 0.007276 | 5144.50 | 764.32 | 513.17 | 116.24 |
| Fst | 14313 | 28.01 | 0.007296 | 338.17 | 140.65 | 10.67 | 5.62 |
| Fem1b | 14155 | 7.35 | 0.007303 | 7161.17 | 886.60 | 1422.83 | 789.44 |
| Gls2 | 216456 | 7.42 | 0.007308 | 44.83 | 19.90 | 8.50 | 7.86 |
| Dusp14 | 56405 | 138.02 | 0.007317 | 263.67 | 116.90 | -1.00 | 2.00 |
| Rcor2 | 104383 | 7.75 | 0.007339 | 269.33 | 96.77 | 42.33 | 21.89 |
| Procr | 19124 | 8.24 | 0.007344 | 7726.00 | 2218.84 | 1329.67 | 498.93 |
| Eml1 | 68519 | 25.35 | 0.007374 | 312.17 | 69.72 | 16.67 | 9.39 |
| Prok2 | 50501 | 8.48 | 0.007397 | 2224.50 | 2288.74 | 487.17 | 653.73 |
| Tgm2 | 21817 | 5.15 | 0.007398 | 2790.00 | 795.24 | 807.00 | 543.63 |
| AI504432 | 229694 | 4.08 | 0.007427 | 486.33 | 20.00 | 199.17 | 51.91 |
| Il1a | 16175 | 55.83 | 0.007471 | 15750.10 | 3635.59 | 383.17 | 193.17 |
| Ero1l | 50527 | 7.48 | 0.007685 | 1892.67 | 78.36 | 312.83 | 95.66 |
| Ptp4a1 | 19243 | 5.86 | 0.007698 | 1052.33 | 163.14 | 256.83 | 97.08 |
| Vasn | 246154 | 27.33 | 0.007788 | 2774.83 | 189.43 | 162.33 | 105.27 |
| Kdm6b | 216850 | 22.32 | 0.007920 | 1419.67 | 302.65 | 75.33 | 10.41 |
| Arl5b | 75869 | 16.20 | 0.007921 | 101.50 | 15.79 | 5.00 | 12.77 |
| Gm15645 | 626055 | 6.12 | 0.007989 | 4398.17 | 570.01 | 1014.00 | 339.47 |
| Ccl7 | 20306 | 110.22 | 0.008117 | 1643.33 | 1021.13 | 17.83 | 17.51 |
| Jarid2 | 16468 | 4.57 | 0.008141 | 1036.17 | 343.59 | 359.67 | 79.12 |
| Runx3 | 12399 | 4.05 | 0.008273 | 181.33 | 38.45 | 56.33 | 17.39 |
| Txnrd1 | 50493 | 4.96 | 0.008275 | 32655.43 | 3423.67 | 8928.67 | 2345.90 |
| Unc5b | 107449 | 4.16 | 0.008384 | 197.33 | 37.98 | 70.33 | 38.89 |
| Clk4 | 12750 | 4.53 | 0.008430 | 407.00 | 44.68 | 157.67 | 104.98 |
| Zc3h12a | 230738 | 6.93 | 0.008564 | 520.00 | 73.02 | 97.50 | 36.05 |
| Ccrl2 | 54199 | 20.01 | 0.008606 | 52509.57 | 12571.35 | 3583.17 | 1517.96 |
| Spry2 | 24064 | 5.54 | 0.008629 | 544.00 | 23.64 | 146.83 | 83.31 |
| Arrdc4 | 66412 | 8.47 | 0.008694 | 1368.50 | 236.90 | 257.50 | 165.82 |
| Npy | 109648 | 6.84 | 0.008712 | 279.67 | 46.69 | 70.00 | 57.45 |
| Ccng2 | 12452 | 4.14 | 0.008721 | 6365.17 | 1289.24 | 2082.67 | 700.26 |
| Myc | 17869 | 5.88 | 0.008772 | 159.00 | 42.58 | 36.33 | 19.59 |
| Slc16a1 | 20501 | 5.98 | 0.008802 | 158.00 | 48.00 | 29.83 | 11.37 |
| Siah2 | 20439 | 4.58 | 0.009014 | 1066.83 | 137.77 | 336.17 | 163.54 |
| Atf4 | 11911 | 4.51 | 0.009056 | 6776.83 | 1154.80 | 2305.67 | 1335.64 |
| Apbb3 | 225372 | 4.13 | 0.009080 | 2207.33 | 458.94 | 737.00 | 168.88 |
| Vegfa | 22339 | 30.77 | 0.009228 | 2036.67 | 683.62 | 96.67 | 54.22 |
| Egr3 | 13655 | 12.38 | 0.009384 | 87.50 | 41.63 | 11.33 | 9.88 |
| Kctd12 | 239217 | 6.55 | 0.009433 | 825.83 | 214.66 | 179.33 | 95.70 |
| Sdc4 | 20971 | 5.09 | 0.009469 | 1481.17 | 78.91 | 452.50 | 125.30 |
| Id1 | 15901 | 16.29 | 0.009507 | 205.00 | 118.53 | 16.67 | 9.02 |
| Epha4 | 13838 | 17.41 | 0.009514 | 1396.00 | 216.26 | 111.50 | 46.35 |
| Plk3 | 12795 | 9.06 | 0.009595 | 4249.50 | 1508.19 | 729.17 | 195.69 |
| Dusp8 | 18218 | 4.59 | 0.009633 | 115.33 | 25.17 | 35.17 | 17.72 |
| Hbegf | 15200 | 227.64 | 0.009633 | 10594.33 | 4649.71 | 73.17 | 55.50 |
| Skil | 20482 | 4.46 | 0.009635 | 576.67 | 93.22 | 178.83 | 65.62 |
| Itgav | 16410 | 11.01 | 0.009791 | 166.17 | 8.69 | 22.17 | 9.22 |
| Adamts9 | 101401 | 63.27 | 0.009805 | 615.50 | 79.07 | 11.50 | 13.45 |
| Ifrd1 | 15982 | 16.31 | 0.009839 | 51796.17 | 15217.02 | 4536.17 | 2003.60 |
| Atf3 | 11910 | 6.05 | 0.009961 | 32537.03 | 1644.52 | 8075.67 | 4602.89 |
| Mmp3 | 17392 | 23.62 | 0.010007 | 365.33 | 191.06 | 17.50 | 7.86 |
| Prosc | 114863 | 4.36 | 0.010009 | 18340.80 | 2923.14 | 5843.83 | 2049.34 |
| Nufip1 | 27275 | 4.14 | 0.010013 | 5092.17 | 1419.77 | 1626.67 | 352.87 |
| Tgm2 | 21817 | 5.09 | 0.010027 | 1870.67 | 590.21 | 563.33 | 396.36 |
| Gm6377 | 622976 | 20.80 | 0.010067 | 163.83 | 49.20 | 10.83 | 12.83 |
| Cth | 107869 | 6.15 | 0.010080 | 515.33 | 111.89 | 153.83 | 103.28 |
| Cflar | 12633 | 4.13 | 0.010084 | 38962.67 | 1083.28 | 12708.33 | 4913.80 |
| Gem | 14579 | 64.76 | 0.010142 | 2738.17 | 572.39 | 68.50 | 55.86 |
| Tnf | 21926 | 29.39 | 0.010181 | 24948.97 | 12295.73 | 1139.83 | 453.29 |
| Thbs1 | 21825 | 7.88 | 0.010283 | 15924.67 | 2939.43 | 2849.00 | 503.71 |
| Mrpl52 | 68836 | 7.50 | 0.010284 | 1482.67 | 441.28 | 261.67 | 70.73 |
| Asb4 | 65255 | 6.48 | 0.010475 | 86.83 | 19.91 | 12.17 | 9.70 |
| Odc1 | 18263 | 8.67 | 0.010561 | 10995.00 | 3516.71 | 1696.17 | 508.25 |
| Thbs1 | 21825 | 7.47 | 0.010690 | 1779.33 | 231.39 | 350.17 | 86.81 |
| Arl5b | 75869 | 27.21 | 0.010742 | 417.50 | 168.91 | 21.67 | 11.50 |
| Arc | 11838 | 20.26 | 0.010790 | 939.33 | 339.70 | 55.00 | 21.02 |
| Qpct | 70536 | 4.56 | 0.010828 | 354.50 | 50.21 | 112.00 | 33.18 |
| Nfkbib | 18036 | 4.43 | 0.010860 | 8234.17 | 378.33 | 2734.83 | 911.55 |
| Zfp36l1 | 12192 | 4.20 | 0.010869 | 3851.67 | 243.43 | 1211.83 | 385.56 |
| Tnfaip3 | 21929 | 11.21 | 0.011017 | 18134.13 | 4364.15 | 2460.00 | 1369.54 |
| Cbln3 | 56410 | 4.11 | 0.011071 | 87.50 | 31.32 | 33.50 | 25.46 |
| P4ha2 | 18452 | 7.56 | 0.011123 | 2287.17 | 412.49 | 487.33 | 145.20 |
| Chst11 | 58250 | 9.02 | 0.011128 | 107.00 | 59.47 | 16.33 | 4.62 |
| Bcl2l11 | 12125 | 8.73 | 0.011147 | 367.33 | 114.81 | 58.67 | 7.57 |
| Plk2 | 20620 | 20.38 | 0.011237 | 11673.50 | 1867.68 | 843.33 | 490.53 |
| Ttc39b | 69863 | 6.71 | 0.011285 | 627.83 | 44.81 | 128.33 | 42.12 |
| Trem1 | 58217 | 11.03 | 0.011294 | 676.50 | 296.25 | 84.83 | 44.38 |
| Pcf11 | 74737 | 4.76 | 0.011313 | 119.33 | 20.51 | 33.83 | 16.00 |
| Ext1 | 14042 | 5.08 | 0.011422 | 1181.33 | 420.69 | 374.67 | 262.00 |
| Arl4a | 11861 | 7.17 | 0.011446 | 1269.33 | 178.61 | 253.00 | 116.57 |
| Mxi1 | 17859 | 4.69 | 0.011617 | 4299.50 | 510.79 | 1235.83 | 150.32 |
| Fem1b | 14155 | 11.28 | 0.011675 | 1264.83 | 432.59 | 142.83 | 43.76 |
| Slc16a1 | 20501 | 10.32 | 0.011709 | 3005.00 | 759.98 | 442.00 | 254.77 |
| Eno2 | 13807 | 6.72 | 0.011725 | 1544.00 | 1017.97 | 401.50 | 430.20 |
| Vegfa | 22339 | 8.01 | 0.011738 | 190.33 | 26.16 | 33.67 | 21.22 |
| Clic4 | 29876 | 7.96 | 0.011741 | 160.50 | 21.02 | 24.17 | 23.27 |
| Zfp131 | 72465 | 5.28 | 0.011936 | 6430.33 | 1203.81 | 1611.67 | 378.92 |
| Zfp655 | 72611 | 4.33 | 0.011953 | 8398.67 | 648.20 | 2784.67 | 1442.66 |
| Slc7a11 | 26570 | 11.18 | 0.011962 | 519.00 | 109.00 | 81.83 | 49.10 |
| Mtmr7 | 54384 | 7.72 | 0.011968 | 26.17 | 10.26 | 3.17 | 4.93 |
| Crem | 12916 | 13.23 | 0.012100 | 7485.50 | 2380.71 | 753.67 | 203.71 |
| Fam110c | 104943 | 13.87 | 0.012214 | 97.17 | 54.36 | 9.00 | 12.62 |
| Ccrn4l | 12457 | 17.09 | 0.012231 | 1822.00 | 549.57 | 140.00 | 57.66 |
| Nfkb1 | 18033 | 4.71 | 0.012269 | 1565.17 | 99.51 | 479.33 | 187.13 |
| Cdkn1a | 12575 | 5.30 | 0.012294 | 4931.17 | 883.18 | 1240.33 | 133.02 |
| Smcr8 | 237782 | 4.23 | 0.012492 | 199.00 | 57.23 | 73.00 | 66.14 |
| Il1a | 16175 | 52.57 | 0.012496 | 8252.17 | 3214.38 | 196.17 | 67.27 |
| Arl5b | 75869 | 7.29 | 0.012528 | 4734.00 | 668.76 | 953.33 | 468.69 |
| Fhad1 | 329977 | 5.83 | 0.012574 | 75.00 | 14.40 | 15.17 | 9.45 |
| Acap2 | 78618 | 5.99 | 0.012636 | 653.17 | 161.63 | 142.17 | 46.86 |
| Pitpnc1 | 71795 | 5.53 | 0.012640 | 245.17 | 31.27 | 76.67 | 50.09 |
| Srxn1 | 76650 | 5.19 | 0.012771 | 17195.03 | 5001.27 | 4586.33 | 972.99 |
| Tubb6 | 67951 | 6.23 | 0.012777 | 40239.90 | 7386.94 | 8677.67 | 3049.28 |
| Tnfrsf9 | 21942 | 8.21 | 0.012810 | 108.33 | 34.02 | 16.67 | 18.50 |
| Arid5a | 214855 | 6.12 | 0.012919 | 565.83 | 39.17 | 129.83 | 65.68 |
| Arl4a | 11861 | 6.36 | 0.013013 | 4848.83 | 832.98 | 1032.00 | 403.05 |
| Agpat9 | 231510 | 9.30 | 0.013167 | 1647.83 | 163.78 | 304.67 | 43.66 |
| Errfi1 | 74155 | 8.34 | 0.013207 | 1946.00 | 616.29 | 304.33 | 64.71 |
| P2ry2 | 18442 | 4.53 | 0.013207 | 1784.50 | 380.41 | 588.50 | 365.96 |
| Osgin2 | 209212 | 5.33 | 0.013211 | 1037.50 | 303.25 | 265.33 | 70.95 |
| Inhbb | 16324 | 7.87 | 0.013250 | 75.00 | 23.97 | 10.00 | 4.82 |
| Tmem144 | 70652 | 5.78 | 0.013267 | 122.83 | 14.37 | 36.00 | 20.02 |
| Junb | 16477 | 5.67 | 0.013315 | 75089.70 | 14957.10 | 16828.70 | 4222.05 |
| Mcl1 | 17210 | 4.96 | 0.013324 | 23630.10 | 1957.21 | 6550.33 | 2756.16 |
| Eea1 | 216238 | 5.35 | 0.013372 | 301.83 | 35.90 | 98.50 | 73.23 |
| Sipa1l1 | 217692 | 4.13 | 0.013574 | 5611.33 | 1828.93 | 1867.50 | 78.73 |
| Cd24a | 12484 | 4.55 | 0.013635 | 3036.67 | 568.68 | 1011.67 | 660.01 |
| Slc7a2 | 11988 | 8.68 | 0.013643 | 733.50 | 50.65 | 123.67 | 65.16 |
| Bcl2a1c | 12046 | 5.54 | 0.013662 | 2897.33 | 324.98 | 785.17 | 444.64 |
| Epha4 | 13838 | 10.63 | 0.013789 | 239.67 | 38.09 | 34.67 | 33.50 |
| Stk38l | 232533 | 4.58 | 0.013827 | 2468.00 | 391.21 | 722.33 | 286.71 |
| Rab44 | 442827 | 5.06 | 0.013891 | 125.17 | 46.78 | 27.67 | 5.75 |
| Zfp36l1 | 12192 | 4.28 | 0.013892 | 4101.50 | 471.25 | 1300.00 | 412.58 |
| Dusp1 | 19252 | 7.02 | 0.013952 | 6511.67 | 3047.19 | 1275.00 | 261.90 |
| Cuedc1 | 103841 | 7.31 | 0.014057 | 1179.33 | 170.81 | 199.50 | 110.94 |
| Hk1 | 15275 | 5.09 | 0.014130 | 676.00 | 128.84 | 205.17 | 88.58 |
| Crem | 12916 | 12.21 | 0.014188 | 6441.33 | 2100.86 | 682.17 | 126.48 |
| Crem | 12916 | 13.87 | 0.014198 | 6383.67 | 2558.90 | 646.83 | 148.64 |
| Smad7 | 17131 | 5.62 | 0.014254 | 226.50 | 77.10 | 57.67 | 32.15 |
| Ier3 | 15937 | 4.70 | 0.014272 | 31188.43 | 7307.75 | 8050.00 | 840.53 |
| Srxn1 | 76650 | 4.98 | 0.014316 | 1700.50 | 345.84 | 470.00 | 151.98 |
| Srxn1 | 76650 | 4.90 | 0.014357 | 1624.00 | 330.98 | 461.67 | 158.71 |
| Rab20 | 19332 | 6.11 | 0.014432 | 8794.17 | 994.65 | 1979.17 | 788.19 |
| Mxd1 | 17119 | 5.07 | 0.014455 | 403.17 | 28.57 | 125.33 | 72.02 |
| Cflar | 12633 | 5.08 | 0.014663 | 657.83 | 52.44 | 195.33 | 124.78 |
| Med13 | 327987 | 5.09 | 0.014733 | 313.00 | 45.19 | 88.83 | 40.97 |
| Cytip | 227929 | 4.56 | 0.014814 | 2511.83 | 1234.43 | 682.33 | 139.74 |
| Zfp655 | 72611 | 4.14 | 0.014817 | 430.00 | 41.87 | 164.17 | 109.46 |
| Cxcr7 | 12778 | 31.64 | 0.014825 | 1546.83 | 164.56 | 73.83 | 38.00 |
| Gm129 | 229599 | 55.82 | 0.014864 | 592.33 | 67.85 | 19.17 | 16.25 |
| Slc25a25 | 227731 | 9.31 | 0.014941 | 2061.33 | 594.36 | 340.33 | 166.81 |
| Fam107b | 66540 | 4.90 | 0.015093 | 2238.67 | 293.82 | 628.50 | 119.31 |
| Dusp10 | 63953 | 8.49 | 0.015102 | 144.50 | 44.45 | 23.50 | 4.09 |
| Mafk | 17135 | 5.76 | 0.015122 | 2006.33 | 192.03 | 467.00 | 186.50 |
| Hivep3 | 16656 | 7.70 | 0.015134 | 453.33 | 120.30 | 87.83 | 46.61 |
| Chaf1b | 110749 | 14.07 | 0.015145 | 1644.83 | 321.47 | 153.17 | 72.40 |
| Coq10b | 67876 | 6.96 | 0.015198 | 726.00 | 139.37 | 159.83 | 102.97 |
| Prok2 | 50501 | 7.98 | 0.015318 | 2632.00 | 2664.99 | 662.17 | 923.05 |
| Map3k2 | 26405 | 4.08 | 0.015448 | 54.33 | 2.93 | 17.83 | 11.62 |
| Gm5662 | 435337 | 4.28 | 0.015750 | 1495.00 | 173.69 | 511.67 | 307.96 |
| Fam162a | 70186 | 5.26 | 0.015767 | 4768.50 | 1498.54 | 1169.33 | 348.01 |
| Tmem144 | 70652 | 4.08 | 0.015865 | 88.83 | 9.46 | 36.83 | 22.22 |
| Lonrf3 | 74365 | 4.31 | 0.015957 | 6833.50 | 1209.32 | 2344.33 | 1026.59 |
| Spag9 | 70834 | 5.62 | 0.016238 | 1375.50 | 359.60 | 365.17 | 266.29 |
| Ralgapa2 | 241694 | 5.87 | 0.016316 | 64.17 | 20.64 | 16.83 | 17.97 |
| F2r | 14062 | 4.78 | 0.016477 | 40.17 | 16.27 | 9.50 | 6.76 |
| Pvr | 52118 | 6.57 | 0.016478 | 135.00 | 12.53 | 31.33 | 18.33 |
| Ccr1 | 12768 | 6.98 | 0.016484 | 52.00 | 11.14 | 15.50 | 9.26 |
| Tnfrsf9 | 21942 | 4.06 | 0.016502 | 31.33 | 22.78 | 8.83 | 11.85 |
| Prosc | 114863 | 6.56 | 0.016583 | 1220.33 | 400.51 | 245.83 | 83.02 |
| Impact | 16210 | 4.27 | 0.016636 | 6020.83 | 133.59 | 1955.17 | 968.86 |
| Smox | 228608 | 4.98 | 0.016663 | 356.00 | 99.16 | 86.50 | 31.78 |
| Slc25a25 | 227731 | 7.60 | 0.016906 | 3601.50 | 610.54 | 684.50 | 367.64 |
| Gadd45b | 17873 | 15.15 | 0.016920 | 7025.67 | 2244.12 | 654.33 | 320.67 |
| Aff4 | 93736 | 5.09 | 0.016931 | 92.00 | 17.84 | 23.33 | 20.40 |
| Macrod2 | 72899 | 4.86 | 0.016968 | 46.50 | 8.05 | 12.50 | 13.26 |
| Id3 | 15903 | 8.96 | 0.017009 | 2245.17 | 865.11 | 325.17 | 148.49 |
| Snx18 | 170625 | 4.87 | 0.017042 | 2265.17 | 591.41 | 591.83 | 127.23 |
| Tpi1 | 21991 | 4.67 | 0.017144 | 26528.83 | 4357.93 | 7912.67 | 2194.33 |
| Hecw2 | 329152 | 5.51 | 0.017189 | 51.00 | 14.40 | 11.00 | 7.94 |
| Arl5b | 75869 | 21.91 | 0.017426 | 304.50 | 92.36 | 17.83 | 13.05 |
| Slc7a11 | 26570 | 9.78 | 0.017490 | 1572.50 | 114.59 | 272.50 | 208.96 |
| Cflar | 12633 | 4.68 | 0.017632 | 365.00 | 48.57 | 108.00 | 36.75 |
| Ell2 | 192657 | 4.73 | 0.017639 | 3160.00 | 410.76 | 882.00 | 268.71 |
| Lin54 | 231506 | 4.04 | 0.017667 | 798.33 | 289.63 | 263.50 | 95.11 |
| Fbxo30 | 71865 | 4.07 | 0.017711 | 993.83 | 152.99 | 342.17 | 156.50 |
| Ahr | 11622 | 4.37 | 0.017864 | 561.17 | 164.27 | 164.17 | 32.45 |
| Myo1b | 17912 | 4.28 | 0.017874 | 526.83 | 38.39 | 186.17 | 109.75 |
| Slc39a14 | 213053 | 9.46 | 0.017983 | 489.33 | 59.13 | 78.00 | 55.16 |
| Pvr | 52118 | 8.80 | 0.017990 | 3827.17 | 964.16 | 565.50 | 178.99 |
| Lpar1 | 14745 | 4.63 | 0.018057 | 171.00 | 9.26 | 50.17 | 7.65 |
| Cd80 | 12519 | 5.32 | 0.018063 | 532.50 | 143.32 | 138.33 | 36.59 |
| Gcnt2 | 14538 | 7.07 | 0.018080 | 43.67 | 14.01 | 11.50 | 11.30 |
| Eif2c2 | 239528 | 4.38 | 0.018112 | 614.17 | 47.78 | 201.83 | 86.27 |
| Rybp | 56353 | 7.14 | 0.018128 | 953.17 | 106.05 | 184.17 | 86.27 |
| Pfkfb3 | 170768 | 4.82 | 0.018144 | 10103.17 | 1269.74 | 2774.67 | 464.66 |
| Sap30 | 60406 | 4.72 | 0.018156 | 2589.83 | 461.55 | 816.33 | 209.29 |
| Hivep2 | 15273 | 8.15 | 0.018286 | 205.83 | 24.27 | 46.50 | 40.61 |
| Pfkfb3 | 170768 | 4.16 | 0.018413 | 157.33 | 17.00 | 51.17 | 6.01 |
| Ppp1r15a | 17872 | 9.54 | 0.018449 | 8190.83 | 1322.52 | 1176.33 | 582.32 |
| Sec24a | 77371 | 6.75 | 0.018747 | 185.83 | 21.65 | 48.67 | 43.94 |
| Nfkb1 | 18033 | 4.55 | 0.019046 | 1491.83 | 79.12 | 430.00 | 191.83 |
| Coq10b | 67876 | 6.36 | 0.019107 | 1191.67 | 217.95 | 244.50 | 96.55 |
| Plau | 18792 | 18.69 | 0.019179 | 3074.67 | 149.38 | 286.33 | 257.60 |
| Myc | 17869 | 4.91 | 0.019332 | 301.17 | 90.64 | 78.50 | 28.32 |
| Fem1c | 240263 | 4.77 | 0.019334 | 1128.67 | 128.00 | 350.50 | 206.55 |
| Stx11 | 74732 | 6.70 | 0.019358 | 2290.83 | 97.45 | 585.83 | 452.28 |
| Cry1 | 12952 | 4.45 | 0.019417 | 64.67 | 13.48 | 20.17 | 8.22 |
| Spag9 | 70834 | 6.08 | 0.019461 | 1209.17 | 248.46 | 295.50 | 209.67 |
| Egln3 | 112407 | 14.12 | 0.019495 | 1968.17 | 1367.96 | 159.50 | 21.96 |
| Cflar | 12633 | 4.72 | 0.019573 | 1992.83 | 71.28 | 576.67 | 260.62 |
| Eno2 | 13807 | 6.47 | 0.019588 | 1910.33 | 1281.58 | 522.67 | 579.00 |
| Eid3 | 66341 | 17.01 | 0.019788 | 289.50 | 24.56 | 26.67 | 27.39 |
| Slc7a11 | 26570 | 9.90 | 0.020188 | 583.00 | 47.27 | 84.67 | 68.73 |
| Lmnb1 | 16906 | 19.98 | 0.020235 | 1846.17 | 1311.95 | 99.17 | 19.21 |
| Denr | 68184 | 4.13 | 0.020363 | 3266.17 | 533.51 | 1075.83 | 442.77 |
| Ramp3 | 56089 | 12.53 | 0.020417 | 3473.00 | 589.00 | 450.83 | 322.93 |
| Ptp4a1 | 19243 | 5.42 | 0.020438 | 3991.33 | 870.03 | 975.17 | 358.87 |
| Cd86 | 12524 | 5.22 | 0.020526 | 2829.17 | 445.82 | 723.17 | 296.61 |
| Fam162a | 70186 | 6.29 | 0.020590 | 51.50 | 20.75 | 8.50 | 13.33 |
| Thbs1 | 21825 | 7.28 | 0.020614 | 2271.83 | 329.51 | 454.67 | 154.13 |
| Ldha | 16828 | 4.14 | 0.020639 | 64582.80 | 11368.71 | 22229.90 | 11537.81 |
| Pim3 | 223775 | 6.49 | 0.020676 | 5491.17 | 867.13 | 1141.83 | 475.69 |
| Usp53 | 99526 | 6.46 | 0.020691 | 521.00 | 84.18 | 122.50 | 72.60 |
| Ccl2 | 20296 | 31.37 | 0.020723 | 8276.50 | 5843.51 | 360.83 | 332.36 |
| Gdf15 | 23886 | 45.16 | 0.020813 | 2116.17 | 840.27 | 63.50 | 52.02 |
| Dusp16 | 70686 | 5.45 | 0.020913 | 130.00 | 21.28 | 36.50 | 32.50 |
| Tnfrsf12a | 27279 | 7.63 | 0.020975 | 3483.83 | 949.58 | 642.83 | 302.91 |
| Ankrd33b | 67434 | 4.30 | 0.021134 | 1461.17 | 201.18 | 504.00 | 277.43 |
| Junb | 16477 | 5.59 | 0.021153 | 90882.13 | 26344.79 | 18431.33 | 3039.72 |
| Zc3hav1l | 209032 | 5.56 | 0.021186 | 88.33 | 32.30 | 23.50 | 23.52 |
| P2ry2 | 18442 | 4.90 | 0.021219 | 9920.00 | 341.35 | 2881.33 | 1596.51 |
| Dpys | 64705 | 7.85 | 0.021431 | 237.83 | 3.33 | 48.33 | 32.75 |
| Stfa3 | 20863 | 6.07 | 0.021537 | 274.33 | 51.92 | 89.83 | 73.59 |
| Crem | 12916 | 10.15 | 0.021592 | 2477.17 | 1070.80 | 332.50 | 134.27 |
| Chst11 | 58250 | 10.44 | 0.021789 | 183.67 | 64.84 | 32.83 | 23.84 |
| Fbxo30 | 71865 | 6.41 | 0.021898 | 677.00 | 66.19 | 163.17 | 97.35 |
| Hsd17b12 | 56348 | 4.61 | 0.022089 | 47.00 | 1.73 | 17.17 | 13.66 |
| Pou3f1 | 18991 | 7.98 | 0.022433 | 426.17 | 155.39 | 69.00 | 23.64 |
| Eif2c2 | 239528 | 4.22 | 0.022679 | 1372.67 | 347.76 | 474.67 | 144.98 |
| Tmem2 | 83921 | 4.54 | 0.022714 | 876.00 | 288.26 | 295.83 | 204.58 |
| Ankrd33b | 67434 | 13.34 | 0.022748 | 2838.17 | 1029.92 | 313.50 | 180.87 |
| Pdpn | 14726 | 12.05 | 0.022784 | 174.17 | 44.92 | 32.50 | 38.66 |
| Csrnp1 | 215418 | 7.56 | 0.022812 | 1303.33 | 267.50 | 250.50 | 127.56 |
| Coq10b | 67876 | 5.32 | 0.022845 | 2882.17 | 515.25 | 778.50 | 398.56 |
| Pvr | 52118 | 13.67 | 0.023418 | 1730.67 | 320.43 | 188.50 | 121.16 |
| Niacr1 | 80885 | 10.92 | 0.023467 | 25883.00 | 2490.77 | 3564.83 | 2457.89 |
| Rnd1 | 223881 | 5.90 | 0.023518 | 806.67 | 338.10 | 192.33 | 67.34 |
| Mapk15 | 332110 | 4.53 | 0.023611 | 174.17 | 46.93 | 61.50 | 50.10 |
| Cgref1 | 68567 | 18.55 | 0.023760 | 839.83 | 545.60 | 63.33 | 34.49 |
| Il1f6 | 54448 | 47.20 | 0.023779 | 229.17 | 53.58 | 7.67 | 12.87 |
| Zc3h12c | 244871 | 4.05 | 0.024048 | 995.67 | 98.40 | 366.67 | 247.84 |
| Mtmr7 | 54384 | 10.61 | 0.024075 | 37.33 | 6.33 | 1.83 | 9.25 |
| Tnfsf9 | 21950 | 32.11 | 0.024249 | 716.50 | 157.62 | 41.00 | 45.97 |
| Akap2 | 11641 | 6.75 | 0.024440 | 32.17 | 5.30 | 5.50 | 7.76 |
| Slc15a3 | 65221 | 4.91 | 0.024583 | 9247.83 | 980.46 | 3099.00 | 1534.19 |
| Ereg | 13874 | 33.16 | 0.024631 | 63.33 | 17.01 | 3.33 | 4.04 |
| Trim69 | 70928 | 6.31 | 0.024969 | 125.50 | 33.01 | 26.67 | 27.21 |
| Dgkg | 110197 | 5.34 | 0.025057 | 273.67 | 101.99 | 76.17 | 62.34 |
| Dot1l | 208266 | 4.67 | 0.025230 | 926.00 | 154.21 | 335.67 | 226.49 |
| Ucp1 | 22227 | 4.34 | 0.025394 | 66.33 | 20.13 | 22.67 | 7.11 |
| Traf1 | 22029 | 5.99 | 0.025542 | 2811.33 | 1390.46 | 633.50 | 136.37 |
| Plcxd1 | 403178 | 4.97 | 0.025661 | 59.00 | 7.26 | 19.67 | 23.71 |
| Abl2 | 11352 | 4.75 | 0.025706 | 82.17 | 11.51 | 24.67 | 18.54 |
| Fam198b | 68659 | 4.43 | 0.025944 | 61.00 | 9.12 | 19.33 | 13.77 |
| Plagl1 | 22634 | 7.50 | 0.026297 | 315.33 | 30.92 | 65.17 | 57.08 |
| Kctd4 | 67516 | 8.93 | 0.026580 | 100.00 | 11.53 | 16.33 | 20.79 |
| Dusp16 | 70686 | 4.58 | 0.027008 | 2583.83 | 168.59 | 993.67 | 590.68 |
| Tpbg | 21983 | 4.36 | 0.027069 | 83.67 | 19.55 | 23.83 | 11.37 |
| Pla2g5 | 18784 | 4.49 | 0.027323 | 86.83 | 2.57 | 30.67 | 21.10 |
| Serpina3f | 238393 | 7.94 | 0.027570 | 19.50 | 12.03 | 4.17 | 8.46 |
| Prr15 | 78004 | 4.73 | 0.027837 | 70.33 | 29.67 | 29.50 | 18.86 |
| Dusp14 | 56405 | 8.60 | 0.028073 | 146.83 | 75.10 | 18.00 | 8.23 |
| Egr2 | 13654 | 6.38 | 0.028286 | 22284.17 | 5935.46 | 4970.67 | 2385.66 |
| Bmp2 | 12156 | 5.07 | 0.028355 | 193.00 | 86.49 | 48.00 | 5.57 |
| Il10 | 16153 | 78.03 | 0.028671 | 3391.50 | 2248.21 | 47.50 | 37.45 |
| Havcr2 | 171285 | 4.48 | 0.028762 | 70.67 | 28.87 | 27.67 | 22.01 |
| Dusp2 | 13537 | 6.99 | 0.028783 | 1861.17 | 1022.40 | 312.17 | 36.53 |
| Rlim | 19820 | 5.33 | 0.028801 | 130.00 | 4.58 | 31.83 | 22.81 |
| Crem | 12916 | 10.29 | 0.029552 | 2919.50 | 1229.25 | 345.67 | 118.59 |
| Zbtb10 | 229055 | 6.07 | 0.030093 | 66.67 | 11.59 | 19.83 | 15.75 |
| Reln | 19699 | 12.36 | 0.030200 | 251.67 | 58.87 | 28.33 | 27.95 |
| Slc38a1 | 105727 | 5.29 | 0.031778 | 191.00 | 29.82 | 66.83 | 63.52 |
| Pde10a | 23984 | 6.31 | 0.031882 | 338.67 | 89.98 | 65.00 | 17.02 |
| Slfn2 | 20556 | 4.33 | 0.031889 | 7223.00 | 837.52 | 2223.33 | 1076.38 |
| Hes1 | 15205 | 8.67 | 0.032095 | 153.83 | 36.53 | 23.00 | 24.39 |
| Pcdhb4 | 93875 | 12.11 | 0.032449 | 95.83 | 52.82 | 9.50 | 7.86 |
| Arl5b | 75869 | 6.94 | 0.032484 | 139.17 | 7.91 | 26.00 | 23.30 |
| Pdpn | 14726 | 5.68 | 0.032661 | 133.33 | 27.42 | 42.33 | 52.21 |
| Adora2a | 11540 | 5.48 | 0.032909 | 3080.33 | 424.51 | 801.33 | 382.31 |
| Rai14 | 75646 | 8.74 | 0.033041 | 118.83 | 12.27 | 25.33 | 12.01 |
| Nrg1 | 211323 | 5.93 | 0.033318 | 123.83 | 35.79 | 26.00 | 17.26 |
| Grasp | 56149 | 4.80 | 0.033357 | 60.67 | 22.94 | 14.50 | 8.23 |
| Pvr | 52118 | 12.67 | 0.033689 | 10962.00 | 4035.31 | 1041.50 | 498.59 |
| Epha2 | 13836 | 14.46 | 0.034259 | 668.67 | 233.91 | 63.00 | 39.89 |
| Gadd45b | 17873 | 16.30 | 0.034436 | 12723.33 | 3748.06 | 1045.50 | 630.41 |
| Ifi205 | 226695 | 4.89 | 0.034583 | 149.00 | 17.35 | 46.00 | 8.23 |
| Olfr291 | 258410 | 4.12 | 0.034607 | 54.67 | 42.73 | 12.00 | 7.00 |
| Gprc5a | 232431 | 6.05 | 0.034823 | 247.33 | 45.61 | 64.33 | 43.08 |
| Slc16a1 | 20501 | 8.50 | 0.034926 | 1389.83 | 322.27 | 217.33 | 136.43 |
| Rel | 19696 | 10.15 | 0.035197 | 54.00 | 2.00 | 6.50 | 14.08 |
| Igf2bp1 | 140486 | 19.50 | 0.035312 | 85.67 | 25.37 | 5.83 | 18.55 |
| Lrp8 | 16975 | 5.93 | 0.035840 | 24.00 | 4.77 | 3.83 | 9.80 |
| Kctd4 | 67516 | 9.84 | 0.036482 | 187.67 | 33.06 | 29.33 | 25.42 |
| Rasgef1b | 320292 | 4.53 | 0.036824 | 2593.50 | 629.14 | 828.00 | 383.85 |
| Usp53 | 99526 | 5.76 | 0.037375 | 50.00 | 10.15 | 13.17 | 9.88 |
| Eif4e | 13684 | 4.06 | 0.038303 | 2616.00 | 670.38 | 819.33 | 279.93 |
| Fermt2 | 218952 | 4.84 | 0.038597 | 46.50 | 37.57 | 10.83 | 3.69 |
| Tnfaip6 | 21930 | 14.19 | 0.039430 | 64.67 | 1.89 | 9.67 | 13.36 |
| Jsrp1 | 71912 | 5.11 | 0.040006 | 10.33 | 5.03 | 1.00 | 5.29 |
| Slc25a25 | 227731 | 7.22 | 0.041402 | 95.83 | 18.01 | 18.33 | 18.71 |
| Mpzl1 | 68481 | 4.14 | 0.041492 | 315.33 | 124.94 | 86.00 | 20.07 |
| Fam46c | 74645 | 4.27 | 0.041982 | 1937.67 | 639.30 | 702.83 | 436.14 |
| Trim25 | 217069 | 6.32 | 0.042073 | 422.83 | 96.38 | 98.00 | 65.21 |
| Tmem88 | 67020 | 13.57 | 0.042255 | 171.17 | 40.44 | 24.50 | 27.99 |
| Cpeb4 | 67579 | 4.11 | 0.043847 | 453.00 | 39.69 | 181.00 | 133.90 |
| Gadd45g | 23882 | 7.95 | 0.044063 | 7503.00 | 5554.47 | 1034.17 | 594.61 |
| Dusp4 | 319520 | 18.43 | 0.046009 | 1264.00 | 279.36 | 124.50 | 124.08 |
| Edil3 | 13612 | 13.72 | 0.046032 | 67.00 | 39.47 | 7.83 | 1.44 |
| Penk | 18619 | 6.14 | 0.047190 | 210.17 | 70.44 | 62.17 | 56.65 |
| Erdr1 | 170942 | 4.56 | 0.047500 | 20355.93 | 6104.08 | 5938.00 | 3986.90 |
| Cryba4 | 12959 | 5.33 | 0.047767 | 108.17 | 12.77 | 26.33 | 22.21 |
| Plau | 18792 | 25.05 | 0.048006 | 221.67 | 15.18 | 22.17 | 34.98 |
| Sparcl1 | 13602 | 4.62 | 0.048426 | 303.33 | 86.52 | 107.33 | 98.49 |
| Mdm2 | 17246 | 4.05 | 0.048507 | 1738.00 | 288.43 | 596.33 | 330.74 |
| Nfkb1 | 18033 | 5.62 | 0.049499 | 52.67 | 5.69 | 12.67 | 12.77 |

**Table S1B**. **Genes decreased by *C. albicans* in wild type RPM**

The expression values (Exp. Val) of genes decreased (110 genes, ≥4.0-fold, p<0.05, n=3) in wild type (WT) RPM

stimulated with *C. albicans* (CA) for 3 h compared to unstimulated (US) RPM are shown.

|  |  | **WT+CA/WT+US** | | | **WT+CA** | | **WT+US** | | |
| --- | --- | --- | --- | --- | --- | --- | --- | --- | --- |
| **GeneName** | **ID** | **Fold↓** | **p-value** | | **Exp.Val** | **±SD** | **Exp.Val** | | **±SD** |
| Rin2 | 74030 | 8.17 | | 0.00018 | 230.17 | 48.29 | 2219.50 | 698.31 | |
| Sephs2 | 20768 | 5.51 | | 0.00026 | 167.17 | 22.74 | 1114.67 | 93.93 | |
| Ppargc1b | 170826 | 6.56 | | 0.00028 | 47.17 | 5.48 | 389.33 | 125.19 | |
| Abcd2 | 26874 | 12.02 | | 0.00036 | 3.50 | 2.18 | 68.00 | 9.64 | |
| Igf1 | 16000 | 21.67 | | 0.00045 | 3.67 | 3.21 | 181.50 | 53.25 | |
| Slc43a2 | 215113 | 5.23 | | 0.00050 | 101.67 | 20.01 | 675.67 | 128.85 | |
| Gab3 | 210710 | 5.99 | | 0.00051 | 18.83 | 7.75 | 161.83 | 59.28 | |
| Ppargc1b | 170826 | 9.76 | | 0.00059 | 9.83 | 3.62 | 169.50 | 68.59 | |
| Gatc | 384281 | 4.99 | | 0.00076 | 10.00 | 4.58 | 55.00 | 16.64 | |
| Ldlrad3 | 241576 | 5.54 | | 0.00102 | 17.33 | 6.03 | 134.50 | 45.77 | |
| Nfic | 18029 | 4.08 | | 0.00109 | 148.17 | 35.91 | 821.67 | 289.77 | |
| Usp18 | 24110 | 6.06 | | 0.00131 | 127.33 | 48.42 | 1053.33 | 491.96 | |
| Wrb | 71446 | 4.84 | | 0.00147 | 81.67 | 46.78 | 490.83 | 193.46 | |
| Ms4a7 | 109225 | 4.23 | | 0.00150 | 612.50 | 161.96 | 3460.33 | 872.84 | |
| Dfna5 | 54722 | 6.82 | | 0.00170 | 179.50 | 66.41 | 1728.00 | 892.21 | |
| Igf1 | 16000 | 5.69 | | 0.00194 | 72.17 | 11.56 | 488.00 | 134.56 | |
| Ms4a7 | 109225 | 4.29 | | 0.00226 | 381.83 | 98.04 | 2279.33 | 647.77 | |
| Mzf1 | 109889 | 4.37 | | 0.00240 | 4.33 | 6.29 | 45.17 | 18.54 | |
| Fut10 | 171167 | 4.07 | | 0.00244 | 8.33 | 5.97 | 51.67 | 16.59 | |
| Zfp710 | 209225 | 4.70 | | 0.00257 | 1760.67 | 466.44 | 11161.33 | 2453.97 | |
| Cebpa | 12606 | 4.55 | | 0.00259 | 776.67 | 103.45 | 4681.17 | 1149.79 | |
| Xrcc6bp1 | 68876 | 4.78 | | 0.00261 | 60.00 | 8.89 | 389.50 | 120.46 | |
| Engase | 217364 | 6.96 | | 0.00268 | 228.00 | 106.85 | 2088.33 | 398.92 | |
| Arhgap11a | 228482 | 4.50 | | 0.00320 | 22.50 | 9.04 | 123.33 | 40.31 | |
| Itga4 | 16401 | 6.78 | | 0.00430 | 3.67 | 1.76 | 51.17 | 21.23 | |
| Engase | 217364 | 16.27 | | 0.00454 | 217.67 | 112.45 | 4334.67 | 1227.89 | |
| Mblac2 | 72852 | 6.81 | | 0.00468 | 31.50 | 8.85 | 266.00 | 63.39 | |
| Pgm2l1 | 70974 | 4.32 | | 0.00481 | 85.00 | 32.01 | 477.50 | 98.75 | |
| Ttc30b | 72421 | 10.21 | | 0.00483 | 3.00 | 7.55 | 42.50 | 5.77 | |
| BC013712 | 230787 | 5.68 | | 0.00494 | 624.17 | 290.58 | 4556.33 | 2268.78 | |
| Ppargc1b | 170826 | 4.74 | | 0.00558 | 83.67 | 47.85 | 448.67 | 129.75 | |
| Tmem86a | 67893 | 4.60 | | 0.00574 | 346.83 | 57.84 | 2131.17 | 779.88 | |
| Nfam1 | 74039 | 5.70 | | 0.00579 | 258.83 | 9.57 | 1930.33 | 330.34 | |
| Calhm2 | 72691 | 5.29 | | 0.00580 | 95.67 | 21.08 | 678.67 | 149.50 | |
| Zfp61 | 22719 | 4.55 | | 0.00583 | 90.33 | 3.33 | 523.00 | 135.56 | |
| Txnip | 56338 | 5.00 | | 0.00596 | 508.33 | 219.48 | 2992.50 | 784.21 | |
| Snx27 | 76742 | 4.98 | | 0.00610 | 148.33 | 39.83 | 934.33 | 371.15 | |
| Myo1f | 17916 | 4.49 | | 0.00612 | 1511.17 | 334.07 | 8525.67 | 239.33 | |
| Tsen2 | 381802 | 4.73 | | 0.00621 | 84.00 | 15.72 | 530.33 | 129.78 | |
| Idh1 | 15926 | 4.61 | | 0.00631 | 155.83 | 34.63 | 919.00 | 130.51 | |
| Exoc3 | 211446 | 4.84 | | 0.00643 | 248.67 | 49.10 | 1430.50 | 297.69 | |
| Pygb | 110078 | 4.32 | | 0.00654 | 29.17 | 6.53 | 141.17 | 14.89 | |
| Dennd2a | 209773 | 4.62 | | 0.00672 | 85.33 | 22.67 | 542.33 | 189.73 | |
| Dock8 | 76088 | 7.17 | | 0.00696 | 87.17 | 35.83 | 812.00 | 169.39 | |
| Rab3a | 19339 | 5.66 | | 0.00712 | 13.67 | 0.58 | 123.33 | 27.34 | |
| Mlh3 | 217716 | 4.54 | | 0.00714 | 8.67 | 4.04 | 59.17 | 14.37 | |
| Fam105a | 223433 | 5.29 | | 0.00714 | 63.83 | 9.31 | 456.83 | 218.46 | |
| Arhgap22 | 239027 | 6.93 | | 0.00717 | 164.67 | 37.58 | 1536.00 | 646.05 | |
| Efcab4a | 213573 | 5.28 | | 0.00726 | 156.00 | 48.81 | 1207.83 | 399.45 | |
| Fam78a | 241303 | 6.52 | | 0.00734 | 35.33 | 18.61 | 366.00 | 103.81 | |
| Snx27 | 76742 | 4.59 | | 0.00735 | 185.83 | 40.97 | 1418.83 | 744.81 | |
| Nfam1 | 74039 | 6.18 | | 0.00747 | 44.67 | 14.19 | 372.83 | 72.07 | |
| Trp53inp1 | 60599 | 4.27 | | 0.00783 | 78.50 | 2.29 | 487.67 | 72.18 | |
| Phlda3 | 27280 | 7.56 | | 0.00792 | 42.83 | 11.47 | 431.67 | 120.60 | |
| Mxd4 | 17122 | 5.66 | | 0.00805 | 395.83 | 26.28 | 3045.33 | 646.41 | |
| Tmem18 | 211986 | 4.46 | | 0.00806 | 56.50 | 19.75 | 334.83 | 85.63 | |
| Slc43a2 | 215113 | 8.64 | | 0.00860 | 76.67 | 33.13 | 864.83 | 228.27 | |
| Cbx6 | 494448 | 4.45 | | 0.00867 | 182.00 | 12.29 | 1042.17 | 414.63 | |
| Pgap1 | 241062 | 4.35 | | 0.00885 | 10.33 | 11.15 | 74.17 | 19.05 | |
| Mblac2 | 72852 | 5.09 | | 0.00941 | 13.83 | 7.52 | 89.50 | 21.29 | |
| Snx27 | 76742 | 4.92 | | 0.00958 | 91.67 | 38.94 | 616.33 | 368.05 | |
| Kctd12b | 207474 | 4.87 | | 0.00964 | 7.00 | 5.07 | 37.83 | 15.59 | |
| Cd180 | 17079 | 4.29 | | 0.01005 | 5.17 | 3.79 | 38.00 | 25.59 | |
| Dnajc28 | 246738 | 6.82 | | 0.01033 | 27.17 | 15.14 | 226.17 | 77.89 | |
| Lipt2 | 67164 | 4.56 | | 0.01052 | 21.50 | 11.46 | 115.50 | 32.74 | |
| Pstk | 214580 | 4.84 | | 0.01061 | 80.33 | 14.84 | 522.00 | 140.16 | |
| Zkscan4 | 544922 | 16.03 | | 0.01066 | 0.17 | 5.75 | 58.33 | 32.47 | |
| Rnf144b | 218215 | 6.41 | | 0.01070 | 164.67 | 10.61 | 1340.17 | 458.80 | |
| Dkk2 | 56811 | 5.06 | | 0.01192 | 116.33 | 14.29 | 790.17 | 104.89 | |
| Chst14 | 72136 | 4.18 | | 0.01203 | 125.67 | 30.89 | 764.67 | 255.74 | |
| Bahcc1 | 268515 | 4.93 | | 0.01221 | 8.50 | 1.80 | 64.00 | 24.58 | |
| Abcg1 | 11307 | 4.64 | | 0.01323 | 469.83 | 39.14 | 3244.33 | 1712.50 | |
| Serpinb8 | 20725 | 4.90 | | 0.01337 | 115.33 | 54.60 | 737.67 | 231.02 | |
| Apobec1 | 11810 | 6.27 | | 0.01343 | 995.50 | 116.71 | 8702.00 | 1608.63 | |
| Man2a2 | 140481 | 9.97 | | 0.01353 | 7.17 | 5.39 | 106.67 | 7.82 | |
| Pctp | 18559 | 4.55 | | 0.01422 | 12.50 | 2.78 | 90.50 | 16.39 | |
| C85492 | 215494 | 5.00 | | 0.01466 | 107.67 | 9.07 | 682.33 | 171.75 | |
| Ahrr | 11624 | 8.79 | | 0.01469 | 185.50 | 99.19 | 2215.17 | 974.53 | |
| Mrpl15 | 27395 | 4.16 | | 0.01471 | 39.67 | 7.29 | 226.33 | 81.83 | |
| Kcnj10 | 16513 | 25.57 | | 0.01481 | 1.00 | 6.87 | 144.33 | 101.53 | |
| Akr1b10 | 67861 | 4.37 | | 0.01491 | 29.17 | 13.90 | 175.33 | 21.73 | |
| Trim21 | 20821 | 4.19 | | 0.01688 | 33.67 | 10.73 | 185.83 | 55.78 | |
| Ypel2 | 77864 | 7.36 | | 0.01708 | 1.33 | 5.51 | 45.50 | 24.56 | |
| Dbt | 13171 | 4.83 | | 0.01743 | 6.33 | 7.77 | 37.17 | 29.52 | |
| Abcd2 | 26874 | 8.63 | | 0.01748 | 5.50 | 1.32 | 101.00 | 19.67 | |
| Casp2 | 12366 | 4.08 | | 0.01761 | 749.17 | 113.66 | 4152.00 | 1312.41 | |
| Apobec1 | 11810 | 5.22 | | 0.01762 | 72.50 | 27.69 | 433.83 | 54.22 | |
| Zfp94 | 22756 | 9.86 | | 0.01763 | 4.50 | 2.50 | 39.17 | 10.80 | |
| Hmha1 | 70719 | 4.99 | | 0.01780 | 296.50 | 86.97 | 1841.33 | 330.37 | |
| Zbtb8a | 73680 | 4.82 | | 0.01869 | 9.83 | 5.48 | 74.17 | 29.86 | |
| Iigp1 | 60440 | 4.13 | | 0.01874 | 31.83 | 32.27 | 226.67 | 263.63 | |
| Cd200r1 | 57781 | 4.22 | | 0.01887 | 90.83 | 20.03 | 495.17 | 245.76 | |
| Stx17 | 67727 | 4.08 | | 0.01903 | 26.17 | 11.24 | 168.83 | 42.42 | |
| Bmf | 171543 | 10.28 | | 0.01922 | 56.67 | 15.89 | 790.67 | 544.55 | |
| Wbscr27 | 79565 | 5.70 | | 0.01942 | 18.33 | 14.63 | 135.00 | 33.75 | |
| Trub1 | 72133 | 6.61 | | 0.01965 | 6.83 | 5.58 | 65.67 | 24.01 | |
| Cep78 | 208518 | 6.20 | | 0.02038 | 3.83 | 2.93 | 35.50 | 12.32 | |
| Sesn1 | 140742 | 6.93 | | 0.02465 | 386.83 | 65.68 | 4077.50 | 2498.31 | |
| Espl1 | 105988 | 5.20 | | 0.02473 | 36.67 | 13.05 | 222.67 | 76.97 | |
| Zfyve28 | 231125 | 8.05 | | 0.02533 | 10.00 | 2.60 | 116.83 | 45.42 | |
| B3gnt8 | 232984 | 4.54 | | 0.02612 | 66.67 | 27.74 | 461.67 | 138.55 | |
| Mblac1 | 330216 | 4.71 | | 0.02632 | 98.50 | 23.88 | 655.33 | 122.23 | |
| Trmt12 | 68260 | 4.35 | | 0.02691 | 6.33 | 5.62 | 41.50 | 4.77 | |
| Sesn1 | 140742 | 7.36 | | 0.02818 | 44.33 | 11.09 | 488.50 | 223.26 | |
| Lpcat1 | 210992 | 5.07 | | 0.02825 | 28.50 | 12.13 | 168.67 | 38.00 | |
| Oxa1l | 69089 | 4.18 | | 0.03011 | 8.00 | 11.36 | 44.83 | 17.32 | |
| Fgd4 | 224014 | 4.38 | | 0.03077 | 12.17 | 13.29 | 60.00 | 30.87 | |
| Gpr160 | 71862 | 7.43 | | 0.03084 | 26.00 | 16.09 | 270.50 | 141.78 | |
| Plekhg3 | 263406 | 4.15 | | 0.03329 | 3.83 | 6.93 | 35.67 | 13.32 | |
| Ccdc125 | 76041 | 7.30 | | 0.03386 | 3.83 | 4.25 | 59.67 | 21.05 | |
| Gpr155 | 68526 | 4.75 | | 0.03405 | 13.50 | 2.29 | 73.33 | 32.83 | |
| Nuak1 | 77976 | 4.75 | | 0.03446 | 253.67 | 27.18 | 1725.83 | 944.09 | |
| Ifit3 | 15959 | 6.38 | | 0.03688 | 7.67 | 8.81 | 64.33 | 55.28 | |
| Angptl4 | 57875 | 5.25 | | 0.03890 | 259.17 | 72.67 | 1620.83 | 521.59 | |
| Ttc30a1 | 78802 | 6.08 | | 0.03978 | 7.00 | 13.43 | 46.67 | 25.58 | |
| Etaa1 | 68145 | 5.47 | | 0.04395 | 14.83 | 9.71 | 87.67 | 29.94 | |
| Fam110b | 242297 | 4.99 | | 0.04589 | 4.67 | 4.51 | 37.00 | 18.19 | |
| Cd28 | 12487 | 4.46 | | 0.04682 | 15.83 | 19.28 | 94.00 | 33.72 | |
| Pilrb2 | 545812 | 4.03 | | 0.04719 | 58.83 | 17.62 | 311.17 | 60.01 | |
