# Supplementary material for: Cytosolic Phospholipase A2α and Eicosanoids Regulate Expression of Genes in Macrophages Involved in Host Defense and Inflammation
Source: PLoS One. 2013 Jul 25;8(7):e69002. doi: 10.1371/journal.pone.0069002 (PMC3742295; doi:10.1371/journal.pone.0069002)
Supplement: Table S2 — S2A and S2B Genes expressed at (A) lower and (B) higher levels in C. albicans-stimulated cPLA2α+/+ than cPLA2α-/- RPM. (DOC) [file pone.0069002.s002.doc]

**Table S2A**. **Genes expressed at lower levels in *C. albicans*-stimulated cPLA_2_α^+/+^ than cPLA_2_α^-/-^ RPM**

The fold change and p-value of genes expressed at lower levels (86 genes, ≥2.0-fold, p<0.05, n=3) in *C. albicans* (CA)-stimulated cPLA_2_α^+/+^ compared to CA-stimulated cPLA_2_α^-/-^ RPM (WT+CA/KO+CA) are shown. The expression values (Exp.Val) of these genes in CA-stimulated cPLA_2_α^+/+^ RPM (WT+CA) and the fold change (up or down) in CA-stimulated cPLA_2_α^+/+^ RPM compared to unstimulated (US) cPLA_2_α^+/+^ RPM (WT+CA/US) are shown. The expression values (Exp.Val) of the genes in CA-stimulated cPLA_2_α^-/-^ RPM (KO+CA) and fold change (up or down) in CA-stimulated cPLA_2_α^-/-^ RPM compared to unstimulated cPLA_2_α^-/-^ RPM (KO+CA/US) are shown.

|  |  | **WT+CA/KO+CA** | | **WT+CA** | | **WT+CA/US** | | | **KO+CA** | | **KO+CA/US** | | |
| --- | --- | --- | --- | --- | --- | --- | --- | --- | --- | --- | --- | --- | --- |
| **GeneName** | **ID** | **Fold↓** | **p-value** | **Exp.Val** | **±SD** | **Fold** | **regulation** | **p-value** | **Exp.Val** | **±SD** | **Fold** | **regulation** | **p-value** |
| Bhlhe41 | 79362 | 2.67 | 0.00011 | 106.50 | 7.86 | 1.38 | up | 0.20435 | 332.83 | 123.15 | 3.57 | up | 0.02974 |
| Irgm1 | 15944 | 2.52 | 0.00050 | 309.50 | 74.38 | 1.68 | down | 0.01837 | 835.50 | 425.74 | 1.37 | up | 0.16151 |
| Spic | 20728 | 3.55 | 0.00053 | 15.17 | 6.93 | 2.78 | down | 0.02402 | 86.00 | 39.41 | 1.21 | down | 0.40544 |
| Fam26f | 215900 | 4.59 | 0.00058 | 17.00 | 5.50 | 2.71 | down | 0.02943 | 95.50 | 67.15 | 1.20 | down | 0.63435 |
| Slc31a2 | 20530 | 2.23 | 0.00062 | 881.83 | 162.80 | 1.77 | down | 0.01210 | 2010.17 | 589.82 | 1.16 | up | 0.29906 |
| Bid | 12122 | 2.09 | 0.00071 | 215.67 | 67.33 | 1.53 | down | 0.03995 | 397.67 | 64.29 | 1.63 | up | 0.03344 |
| Rasd2 | 75141 | 2.27 | 0.00071 | 75.17 | 36.07 | 1.45 | up | 0.15192 | 146.83 | 29.43 | 3.38 | up | 0.01311 |
| Il15ra | 16169 | 2.10 | 0.00075 | 47.67 | 10.02 | 1.05 | down | 0.81782 | 106.17 | 25.01 | 2.09 | up | 0.04796 |
| Spic | 20728 | 3.33 | 0.00077 | 118.50 | 28.58 | 2.68 | down | 0.02170 | 408.83 | 104.12 | 1.21 | down | 0.52814 |
| Batf2 | 74481 | 2.28 | 0.00079 | 282.33 | 25.30 | 1.82 | down | 0.01802 | 615.17 | 178.34 | 1.04 | down | 0.78399 |
| Gbp3 | 55932 | 3.54 | 0.00103 | 627.00 | 137.58 | 1.65 | down | 0.04558 | 2277.83 | 655.19 | 1.75 | up | 0.01868 |
| Rps6ka2 | 20112 | 2.03 | 0.00111 | 45.17 | 15.28 | 1.98 | up | 0.01098 | 92.17 | 33.79 | 4.35 | up | 0.06244 |
| Naa25 | 231713 | 2.00 | 0.00116 | 69.83 | 18.48 | 1.74 | down | 0.00230 | 164.00 | 53.01 | 1.02 | up | 0.76335 |
| Slc30a1 | 22782 | 2.52 | 0.00117 | 460.83 | 93.25 | 1.71 | up | 0.00280 | 1146.33 | 313.70 | 3.39 | up | 0.03144 |
| Rab33a | 19337 | 2.31 | 0.00124 | 148.67 | 55.75 | 1.38 | up | 0.02344 | 358.67 | 123.51 | 3.20 | up | 0.02715 |
| Grwd1 | 101612 | 2.31 | 0.00125 | 144.67 | 29.26 | 1.70 | down | 0.00339 | 307.83 | 63.02 | 1.37 | up | 0.04860 |
| Irf1 | 16362 | 2.00 | 0.00128 | 13854.50 | 2344.52 | 1.69 | up | 0.11020 | 29789.30 | 14510.26 | 2.88 | up | 0.00917 |
| Gbp6 | 229900 | 3.77 | 0.00135 | 24.33 | 13.33 | 2.63 | down | 0.04316 | 97.83 | 45.64 | 1.28 | up | 0.17317 |
| Pex12 | 103737 | 2.05 | 0.00142 | 50.33 | 22.70 | 1.68 | down | 0.00736 | 102.33 | 48.23 | 1.43 | up | 0.13296 |
| Ddhd1 | 114874 | 2.36 | 0.00145 | 46.50 | 18.05 | 1.93 | down | 0.00299 | 102.00 | 41.80 | 1.11 | down | 0.56474 |
| Irgm1 | 15944 | 2.66 | 0.00149 | 198.33 | 75.08 | 1.84 | down | 0.02378 | 507.67 | 211.64 | 1.50 | up | 0.17399 |
| Glt25d1 | 234407 | 2.04 | 0.00156 | 568.83 | 80.55 | 1.42 | down | 0.04199 | 1349.83 | 467.39 | 1.21 | up | 0.03597 |
| Ly9 | 17085 | 2.54 | 0.00166 | 122.83 | 16.75 | 1.84 | down | 0.00569 | 323.33 | 138.19 | 1.24 | up | 0.67950 |
| Tsr1 | 104662 | 2.64 | 0.00166 | 83.33 | 33.00 | 1.08 | down | 0.13571 | 201.83 | 54.26 | 2.10 | up | 0.00611 |
| Rcn1 | 19672 | 2.58 | 0.00169 | 231.83 | 60.00 | 1.53 | down | 0.00992 | 582.50 | 70.52 | 1.27 | up | 0.05546 |
| Rassf4 | 213391 | 4.67 | 0.00169 | 310.50 | 61.21 | 2.51 | down | 0.02703 | 1454.50 | 444.31 | 1.41 | up | 0.17540 |
| Tnfrsf14 | 230979 | 2.02 | 0.00194 | 21.33 | 6.45 | 2.53 | down | 0.03394 | 45.17 | 16.85 | 2.18 | down | 0.13226 |
| Aen | 68048 | 2.00 | 0.00204 | 318.33 | 74.15 | 1.90 | down | 0.03926 | 606.83 | 188.35 | 1.34 | up | 0.10810 |
| Gpr68 | 238377 | 2.54 | 0.00219 | 131.33 | 20.68 | 4.11 | up | 0.09017 | 301.33 | 60.77 | 6.97 | up | 0.01065 |
| Mgat4b | 103534 | 2.00 | 0.00236 | 512.00 | 113.96 | 1.56 | down | 0.00495 | 1016.17 | 258.55 | 1.07 | down | 0.61422 |
| Ticam2 | 225471 | 4.29 | 0.00239 | 17.83 | 16.75 | 2.23 | down | 0.00754 | 84.83 | 51.25 | 2.07 | up | 0.00144 |
| Slc31a2 | 20530 | 2.13 | 0.00252 | 2057.17 | 568.38 | 2.09 | down | 0.00223 | 4387.67 | 1126.66 | 1.13 | down | 0.43881 |
| Slamf7 | 75345 | 4.07 | 0.00264 | 32.17 | 4.80 | 3.42 | up | 0.06532 | 141.17 | 38.78 | 14.00 | up | 0.02144 |
| Ptpro | 19277 | 2.57 | 0.00303 | 385.67 | 47.91 | 2.32 | down | 0.01340 | 912.83 | 318.42 | 1.25 | down | 0.62836 |
| Pgap1 | 241062 | 2.12 | 0.00304 | 10.33 | 11.15 | 4.35 | down | 0.00885 | 28.83 | 18.82 | 2.60 | down | 0.07945 |
| Fam53b | 77938 | 2.44 | 0.00305 | 35.67 | 7.77 | 3.65 | down | 0.00353 | 84.83 | 44.82 | 1.69 | down | 0.04089 |
| Srm | 20810 | 2.50 | 0.00310 | 312.33 | 56.23 | 1.80 | down | 0.01459 | 922.83 | 186.99 | 1.23 | up | 0.32898 |
| Shmt2 | 108037 | 2.11 | 0.00315 | 592.67 | 159.63 | 1.32 | up | 0.04142 | 1101.17 | 287.32 | 2.27 | up | 0.00546 |
| Cd40 | 21939 | 2.40 | 0.00337 | 334.33 | 31.07 | 1.90 | up | 0.02161 | 837.50 | 212.30 | 3.08 | up | 0.00156 |
| Trmt61a | 328162 | 2.68 | 0.00340 | 68.83 | 21.58 | 2.14 | down | 0.00330 | 205.83 | 86.30 | 1.38 | up | 0.10592 |
| Phgdh | 236539 | 3.11 | 0.00364 | 257.00 | 77.66 | 1.92 | up | 0.01455 | 877.83 | 226.12 | 3.48 | up | 0.01409 |
| Gar1 | 68147 | 2.02 | 0.00377 | 149.83 | 30.86 | 1.31 | down | 0.00169 | 300.67 | 51.70 | 2.27 | up | 0.28213 |
| Timd4 | 276891 | 4.74 | 0.00381 | 261.67 | 25.32 | 1.70 | down | 0.17271 | 1334.33 | 542.53 | 1.36 | down | 0.00985 |
| Grwd1 | 101612 | 2.25 | 0.00415 | 142.67 | 32.15 | 1.71 | down | 0.00094 | 304.33 | 50.24 | 1.25 | up | 0.17603 |
| Slc7a2 | 11988 | 2.00 | 0.00487 | 733.50 | 50.65 | 8.68 | up | 0.01364 | 1473.00 | 385.30 | 17.58 | up | 0.00389 |
| Ppfibp2 | 19024 | 2.70 | 0.00491 | 775.33 | 47.78 | 1.07 | down | 0.69993 | 2091.17 | 290.27 | 2.20 | up | 0.00489 |
| Csf1 | 12977 | 3.43 | 0.00503 | 298.00 | 71.46 | 3.58 | up | 0.04181 | 1070.67 | 253.43 | 9.96 | up | 0.00020 |
| Slc19a1 | 20509 | 2.00 | 0.00539 | 260.67 | 30.01 | 1.02 | up | 0.81058 | 516.00 | 110.48 | 1.70 | up | 0.00029 |
| Kdm4a | 230674 | 2.36 | 0.00570 | 99.83 | 21.77 | 1.21 | down | 0.01615 | 215.17 | 53.49 | 1.85 | up | 0.00168 |
| Rassf4 | 213391 | 3.72 | 0.00572 | 117.17 | 29.89 | 3.62 | down | 0.04084 | 526.83 | 226.14 | 1.46 | down | 0.17541 |
| Mfsd7a | 243197 | 2.93 | 0.00583 | 45.17 | 2.02 | 1.70 | down | 0.04533 | 142.33 | 48.01 | 1.48 | up | 0.23029 |
| Irg1 | 16365 | 2.85 | 0.00591 | 14843.17 | 2241.93 | 2.43 | up | 0.02445 | 42570.20 | 7139.03 | 4.47 | up | 0.02386 |
| Gbp1 | 14468 | 5.69 | 0.00609 | 191.67 | 136.54 | 1.75 | down | 0.01575 | 971.00 | 550.80 | 2.41 | up | 0.02461 |
| Jsrp1 | 71912 | 8.97 | 0.00620 | 10.33 | 5.03 | 5.11 | up | 0.04001 | 104.83 | 34.81 | 17.17 | up | 0.06367 |
| Plau | 18792 | 2.00 | 0.00648 | 221.67 | 15.18 | 25.05 | up | 0.04801 | 474.83 | 203.29 | 37.91 | up | 0.01186 |
| Nolc1 | 70769 | 2.40 | 0.00661 | 19.00 | 6.56 | 2.39 | down | 0.02289 | 46.00 | 19.47 | 1.01 | down | 0.83206 |
| Ifng | 15978 | 3.73 | 0.00666 | 15.50 | 5.22 | 1.45 | up | 0.57245 | 47.17 | 27.61 | 3.30 | up | 0.03791 |
| Afap1l1 | 106877 | 2.30 | 0.00674 | 67.00 | 9.99 | 2.86 | down | 0.00164 | 149.00 | 39.28 | 1.57 | down | 0.07042 |
| Dennd2d | 72121 | 2.58 | 0.00678 | 265.83 | 101.10 | 2.31 | up | 0.03116 | 603.17 | 209.86 | 3.81 | up | 0.01635 |
| Rsad2 | 58185 | 3.28 | 0.00681 | 26.33 | 9.83 | 1.35 | up | 0.22021 | 86.83 | 26.99 | 2.88 | up | 0.02056 |
| Gbp5 | 229898 | 2.63 | 0.00685 | 391.67 | 196.26 | 1.00 | down | 0.92595 | 1042.67 | 496.27 | 2.25 | up | 0.02232 |
| Il20rb | 213208 | 3.34 | 0.00693 | 384.50 | 33.28 | 3.58 | up | 0.02328 | 1671.67 | 602.57 | 5.83 | up | 0.00524 |
| Irf4 | 16364 | 2.77 | 0.00706 | 189.33 | 28.44 | 6.63 | up | 0.00184 | 607.33 | 424.14 | 15.26 | up | 0.00129 |
| Slc48a1 | 67739 | 2.03 | 0.00707 | 2222.00 | 343.79 | 1.46 | down | 0.04272 | 4590.00 | 1026.25 | 1.18 | down | 0.30232 |
| Cdk18 | 18557 | 2.43 | 0.00708 | 472.33 | 46.48 | 1.35 | up | 0.39352 | 1042.67 | 38.02 | 2.71 | up | 0.01790 |
| Rassf4 | 213391 | 4.86 | 0.00724 | 42.33 | 17.56 | 2.52 | down | 0.04908 | 204.50 | 64.50 | 1.47 | up | 0.04351 |
| Fyb | 23880 | 2.04 | 0.00730 | 177.67 | 30.57 | 1.61 | down | 0.01889 | 342.83 | 72.51 | 1.05 | up | 0.84281 |
| Ldlrad3 | 241576 | 2.07 | 0.00822 | 17.33 | 6.03 | 5.54 | down | 0.00102 | 35.17 | 12.00 | 4.16 | down | 0.05121 |
| Fzd7 | 14369 | 2.48 | 0.00830 | 114.67 | 25.54 | 1.47 | up | 0.07247 | 332.33 | 124.11 | 3.89 | up | 0.00205 |
| Ifi47 | 15953 | 3.25 | 0.00843 | 39.33 | 22.19 | 2.58 | down | 0.00909 | 113.83 | 63.62 | 1.15 | down | 0.70657 |
| Ccdc86 | 108673 | 2.23 | 0.00862 | 691.50 | 21.27 | 1.29 | down | 0.11267 | 1635.17 | 223.79 | 1.65 | up | 0.02136 |
| Phgdh | 236539 | 2.70 | 0.00897 | 1871.50 | 513.08 | 2.72 | up | 0.00977 | 4982.17 | 648.29 | 3.88 | up | 0.06150 |
| Nhlrc1 | 105193 | 2.42 | 0.00913 | 48.17 | 25.98 | 2.26 | down | 0.01376 | 123.50 | 35.33 | 1.01 | up | 0.96595 |
| Gbp2 | 14469 | 4.23 | 0.00948 | 448.33 | 257.53 | 1.42 | down | 0.08589 | 1927.17 | 1230.80 | 1.90 | up | 0.02584 |
| Mpa2l | 100702 | 4.40 | 0.00949 | 12.17 | 14.58 | 2.97 | down | 0.00136 | 64.33 | 37.31 | 1.26 | down | 0.18962 |
| Smo | 319757 | 2.21 | 0.00960 | 35.17 | 16.62 | 1.25 | down | 0.39970 | 77.00 | 39.23 | 1.35 | down | 0.03911 |
| Phgdh | 236539 | 2.56 | 0.00971 | 1092.17 | 141.49 | 3.03 | up | 0.00973 | 3410.33 | 940.18 | 4.36 | up | 0.01492 |
| Ifng | 15978 | 3.72 | 0.00986 | 1.67 | 5.58 | 1.14 | down | 0.66142 | 24.33 | 24.95 | 3.44 | up | 0.00317 |
| Ppfibp2 | 19024 | 2.82 | 0.01003 | 2293.83 | 426.87 | 1.04 | up | 0.82563 | 6387.50 | 871.87 | 2.27 | up | 0.01783 |
| Anpep | 16790 | 2.00 | 0.01007 | 1261.00 | 381.22 | 1.09 | down | 0.78469 | 2410.67 | 971.18 | 1.42 | up | 0.04376 |
| Pilra | 231805 | 2.27 | 0.01039 | 243.83 | 35.04 | 1.39 | down | 0.04966 | 492.00 | 106.93 | 1.30 | up | 0.11288 |
| Rsad2 | 58185 | 2.63 | 0.01044 | 29.17 | 10.30 | 1.29 | up | 0.03717 | 75.83 | 25.77 | 2.58 | up | 0.01127 |
| Rsad2 | 58185 | 5.17 | 0.01069 | 128.50 | 44.51 | 1.59 | up | 0.13271 | 660.00 | 214.70 | 3.45 | up | 0.07261 |
| Irg1 | 16365 | 2.53 | 0.01099 | 11911.83 | 1432.20 | 2.58 | up | 0.04882 | 32182.83 | 2895.99 | 3.81 | up | 0.06086 |
| Rrp12 | 107094 | 2.43 | 0.01294 | 24.67 | 16.54 | 1.71 | down | 0.01892 | 61.50 | 35.22 | 1.80 | up | 0.12334 |
| Akna | 100182 | 2.35 | 0.01340 | 474.67 | 116.00 | 1.01 | down | 0.78494 | 1209.17 | 290.68 | 1.79 | up | 0.03991 |
| Cmpk2 | 22169 | 3.36 | 0.01344 | 13.50 | 2.29 | 1.28 | down | 0.03486 | 64.83 | 34.48 | 1.46 | up | 0.44542 |
| Arhgef4 | 226970 | 2.68 | 0.01356 | 15.17 | 2.47 | 1.92 | down | 0.00684 | 48.00 | 17.69 | 1.38 | down | 0.18200 |
| Ccl5 | 20304 | 4.71 | 0.01382 | 404.17 | 20.55 | 1.78 | up | 0.03871 | 1893.00 | 932.49 | 1.51 | up | 0.09782 |
| Slc16a10 | 72472 | 2.09 | 0.01412 | 55.33 | 30.29 | 1.27 | up | 0.27395 | 116.33 | 25.77 | 2.58 | up | 0.02052 |
| Plau | 18792 | 2.00 | 0.01446 | 3074.67 | 149.38 | 18.69 | up | 0.01918 | 5804.67 | 1581.00 | 35.29 | up | 0.00616 |
| Gss | 14854 | 2.00 | 0.01454 | 219.50 | 15.61 | 1.27 | down | 0.05891 | 439.83 | 96.42 | 1.33 | up | 0.00823 |
| Dennd2d | 72121 | 2.56 | 0.01506 | 107.67 | 49.28 | 2.03 | up | 0.07399 | 277.83 | 150.54 | 3.88 | up | 0.00618 |
| Slc30a1 | 22782 | 2.80 | 0.01531 | 383.67 | 230.99 | 1.15 | up | 0.09034 | 967.00 | 425.54 | 2.50 | up | 0.02021 |
| Acsl1 | 14081 | 3.54 | 0.01551 | 107.00 | 42.44 | 1.63 | down | 0.03861 | 394.50 | 179.38 | 1.22 | up | 0.34117 |
| Hivep3 | 16656 | 2.66 | 0.01556 | 453.33 | 120.30 | 7.70 | up | 0.01513 | 1349.33 | 332.41 | 12.14 | up | 0.00075 |
| Akap2 | 11641 | 2.12 | 0.01669 | 32.17 | 5.30 | 6.75 | up | 0.02444 | 68.33 | 27.32 | 11.85 | up | 0.03981 |
| Timm9 | 30056 | 2.09 | 0.01730 | 129.17 | 45.00 | 1.87 | down | 0.01820 | 273.00 | 58.90 | 1.04 | up | 0.83938 |
| Gclm | 14630 | 2.06 | 0.01769 | 93.83 | 7.59 | 1.60 | up | 0.06623 | 196.17 | 31.34 | 2.14 | up | 0.01844 |
| Fam65c | 69553 | 2.03 | 0.01780 | 57.17 | 11.36 | 1.84 | up | 0.27946 | 109.00 | 54.37 | 4.52 | up | 0.00131 |
| Slc6a9 | 14664 | 2.73 | 0.01808 | 1220.00 | 318.21 | 1.46 | down | 0.05534 | 2969.50 | 965.28 | 1.37 | up | 0.02251 |
| Timm9 | 30056 | 2.00 | 0.01985 | 30.00 | 15.80 | 1.97 | down | 0.04822 | 70.67 | 34.24 | 1.13 | up | 0.30534 |
| Cx3cl1 | 20312 | 2.19 | 0.02072 | 18.00 | 4.58 | 2.25 | up | 0.02296 | 40.00 | 32.97 | 2.82 | up | 0.03612 |
| Tnfrsf14 | 230979 | 2.00 | 0.02247 | 57.50 | 1.32 | 2.33 | down | 0.02774 | 117.33 | 20.88 | 2.17 | down | 0.10842 |
| Rapgef5 | 217944 | 2.34 | 0.02257 | 52.50 | 25.11 | 1.96 | down | 0.01119 | 119.83 | 54.91 | 1.06 | down | 0.64029 |
| Irf1 | 16362 | 2.41 | 0.02366 | 408.50 | 139.19 | 1.37 | up | 0.14978 | 1156.83 | 876.72 | 3.18 | up | 0.01429 |
| Irgm2 | 54396 | 2.29 | 0.02415 | 87.17 | 34.40 | 3.29 | down | 0.00050 | 190.33 | 107.93 | 1.72 | down | 0.28361 |
| Slamf7 | 75345 | 2.58 | 0.02437 | 33.50 | 12.01 | 2.60 | up | 0.15472 | 106.00 | 40.36 | 5.67 | up | 0.00279 |
| Slfn5 | 327978 | 2.21 | 0.02470 | 22.00 | 9.76 | 1.10 | down | 0.61549 | 65.50 | 29.25 | 2.03 | up | 0.04227 |
| Tmem140 | 68487 | 2.27 | 0.02518 | 3789.33 | 1962.71 | 3.04 | down | 0.01133 | 8565.00 | 3949.06 | 1.30 | down | 0.11999 |
| Artn | 11876 | 6.12 | 0.02598 | 17.17 | 13.75 | 1.26 | down | 0.49558 | 137.67 | 78.18 | 1.94 | up | 0.00732 |
| Edn1 | 13614 | 4.53 | 0.02654 | 141.33 | 55.72 | 53.33 | up | 0.00341 | 698.50 | 164.28 | 110.61 | up | 0.02452 |
| Acp5 | 11433 | 2.65 | 0.02757 | 3744.17 | 1342.87 | 4.95 | up | 0.00369 | 9575.33 | 461.79 | 8.29 | up | 0.02110 |
| Igf1 | 16000 | 2.46 | 0.02883 | 72.17 | 11.56 | 5.69 | down | 0.00194 | 159.00 | 54.53 | 3.22 | down | 0.07197 |
| Lpl | 16956 | 3.94 | 0.02914 | 1713.17 | 501.69 | 1.47 | down | 0.01515 | 6580.17 | 1717.41 | 1.47 | down | 0.00742 |
| Scarf1 | 380713 | 2.00 | 0.03085 | 732.00 | 28.62 | 1.40 | up | 0.13072 | 1470.83 | 460.69 | 1.93 | up | 0.02814 |
| Lst1 | 16988 | 2.05 | 0.03086 | 42.50 | 6.06 | 1.65 | down | 0.00126 | 82.50 | 10.64 | 1.66 | down | 0.13028 |
| Iigp1 | 60440 | 5.11 | 0.03114 | 31.83 | 32.27 | 4.13 | down | 0.01874 | 157.67 | 119.13 | 1.32 | down | 0.55504 |
| Clec5a | 23845 | 3.28 | 0.03220 | 463.67 | 120.89 | 1.72 | down | 0.03367 | 1411.17 | 374.29 | 1.25 | up | 0.14283 |
| Ccl5 | 20304 | 3.86 | 0.03283 | 1364.50 | 363.09 | 1.78 | up | 0.01831 | 5997.50 | 2941.86 | 1.48 | up | 0.19655 |
| Psme2 | 19188 | 2.00 | 0.03443 | 7503.83 | 831.69 | 1.33 | down | 0.00036 | 14806.33 | 2419.98 | 1.08 | down | 0.69392 |
| Gpatch4 | 66614 | 2.07 | 0.03468 | 496.00 | 45.50 | 1.72 | down | 0.03144 | 1118.17 | 167.75 | 1.08 | up | 0.63645 |
| Ifi205 | 226695 | 2.50 | 0.03475 | 149.00 | 17.35 | 4.89 | up | 0.03458 | 368.50 | 118.61 | 12.32 | up | 0.02514 |
| Tnf | 21926 | 3.62 | 0.03481 | 16589.33 | 8384.98 | 29.39 | up | 0.01018 | 62567.63 | 18143.03 | 76.09 | up | 0.00327 |
| Nans | 94181 | 2.22 | 0.03731 | 498.67 | 101.79 | 1.36 | down | 0.14226 | 976.33 | 112.14 | 1.45 | up | 0.00909 |
| Rassf4 | 213391 | 2.50 | 0.03926 | 407.50 | 88.51 | 2.67 | down | 0.03094 | 1054.17 | 74.97 | 1.34 | down | 0.03871 |
| Trip13 | 69716 | 2.73 | 0.03960 | 49.83 | 8.25 | 1.89 | down | 0.01314 | 186.00 | 89.60 | 1.07 | down | 0.76949 |
| Psme2 | 19188 | 2.05 | 0.04018 | 4697.00 | 476.62 | 1.37 | down | 0.01912 | 9776.33 | 1883.88 | 1.08 | down | 0.67120 |
| Dnmt3a | 13435 | 2.34 | 0.04177 | 91.50 | 25.29 | 1.13 | down | 0.58225 | 220.17 | 9.70 | 1.38 | up | 0.03364 |
| Ipo13 | 230673 | 2.08 | 0.04317 | 916.33 | 139.17 | 1.35 | down | 0.01604 | 1996.50 | 677.16 | 1.16 | up | 0.49542 |
| Ch25h | 12642 | 6.17 | 0.04335 | 80.17 | 31.32 | 2.06 | up | 0.00120 | 573.33 | 265.10 | 7.83 | up | 0.05134 |
| Ak4 | 11639 | 2.33 | 0.04404 | 274.17 | 176.53 | 8.77 | up | 0.00110 | 615.83 | 329.16 | 18.66 | up | 0.00505 |
| Adm | 11535 | 2.99 | 0.04651 | 750.67 | 526.44 | 9.75 | up | 0.00425 | 1917.83 | 1203.47 | 26.81 | up | 0.00056 |
| Rnf213 | 629974 | 2.04 | 0.04747 | 1763.00 | 459.57 | 1.15 | up | 0.49081 | 3330.50 | 199.81 | 1.86 | up | 0.01278 |
| Galnt9 | 231605 | 2.00 | 0.04882 | 22.17 | 6.33 | 2.88 | down | 0.04798 | 51.33 | 5.39 | 2.28 | down | 0.03731 |
| Gdf15 | 23886 | 3.57 | 0.04986 | 2116.17 | 840.27 | 45.16 | up | 0.02081 | 7174.83 | 952.06 | 105.74 | up | 0.01063 |

**Table S2B**. **Genes expressed at higher levels in *C. albicans*-stimulated cPLA_2_α^+/+^ than cPLA_2_α^-/-^ RPM**

The fold change and p-value of genes expressed at higher levels (181 genes, ≥2.0-fold, p<0.05, n=3) in *C. albicans* (CA)-stimulated cPLA_2_α^+/+^ compared to CA-stimulated cPLA_2_α^-/-^ RPM (WT+CA/KO+CA) are shown. The expression values (Exp.Val) of these genes in CA-stimulated cPLA_2_α^+/+^ RPM (WT+CA) and the fold change (up or down) in CA-stimulated cPLA_2_α^+/+^ RPM compared to unstimulated (US) cPLA_2_α^+/+^ RPM (WT+CA/US) are shown. The expression values (Exp.Val) of the genes in CA-stimulated cPLA_2_α^-/-^ RPM (KO+CA) and fold change (up or down) in CA-stimulated cPLA_2_α^-/-^ RPM compared to unstimulated cPLA_2_α^-/-^ RPM (KO+CA/US) are shown.

|  |  | **WT+CA/KO+CA** | | **WT+CA** | | **WT+CA/US** | | | **KO+CA** | | **KO+CA/US** | | |
| --- | --- | --- | --- | --- | --- | --- | --- | --- | --- | --- | --- | --- | --- |
| **GeneName** | **ID** | **Fold↑** | **p-value** | **Exp. Val** | **±SD** | **Fold** | **regulation** | **p-value** | **Exp. Val** | **±SD** | **Fold** | **regulation** | **p-value** |
| Map4k4 | 26921 | 2.23 | 0.00003 | 30592.40 | 4145.20 | 3.96 | up | 0.03369 | 14545.17 | 5385.45 | 2.81 | up | 0.01219 |
| Smox | 228608 | 2.92 | 0.00004 | 7416.50 | 1378.58 | 6.75 | up | 0.00329 | 2315.17 | 566.08 | 3.50 | up | 0.00432 |
| Thbs1 | 21825 | 7.87 | 0.00006 | 1779.33 | 231.39 | 7.47 | up | 0.01069 | 243.50 | 88.83 | 2.18 | up | 0.33527 |
| Map4k4 | 26921 | 2.28 | 0.00013 | 5503.50 | 1398.67 | 3.87 | up | 0.01394 | 2374.67 | 814.73 | 2.45 | up | 0.03895 |
| Thbs1 | 21825 | 8.33 | 0.00015 | 2271.83 | 329.51 | 7.28 | up | 0.02061 | 308.17 | 114.44 | 2.17 | up | 0.33383 |
| Frat2 | 212398 | 3.65 | 0.00017 | 699.00 | 116.20 | 2.21 | up | 0.00371 | 173.33 | 49.35 | 1.53 | down | 0.02858 |
| Pla1a | 85031 | 4.99 | 0.00020 | 2088.17 | 613.91 | 3.64 | up | 0.00791 | 468.33 | 244.56 | 1.69 | up | 0.00215 |
| Qpct | 70536 | 5.89 | 0.00032 | 354.50 | 50.21 | 4.56 | up | 0.01083 | 61.33 | 33.45 | 1.07 | up | 0.61548 |
| Pdpn | 14726 | 4.99 | 0.00033 | 174.17 | 44.92 | 12.05 | up | 0.02278 | 38.83 | 19.09 | 6.43 | up | 0.06646 |
| Cxcr7 | 12778 | 7.09 | 0.00033 | 1546.83 | 164.56 | 31.64 | up | 0.01482 | 221.17 | 75.47 | 6.01 | up | 0.08760 |
| Thbs1 | 21825 | 8.22 | 0.00034 | 15924.67 | 2939.43 | 7.88 | up | 0.01028 | 2200.83 | 676.65 | 2.54 | up | 0.29200 |
| Spry2 | 24064 | 2.68 | 0.00035 | 544.00 | 23.64 | 5.54 | up | 0.00863 | 218.83 | 66.19 | 2.53 | up | 0.04790 |
| Hgf | 15234 | 3.71 | 0.00036 | 70.67 | 16.75 | 1.92 | up | 0.08062 | 21.83 | 14.02 | 1.53 | down | 0.17841 |
| Zfp52 | 22710 | 2.37 | 0.00041 | 844.83 | 118.20 | 1.87 | up | 0.02871 | 368.83 | 100.97 | 1.04 | up | 0.84967 |
| Depdc7 | 211896 | 2.54 | 0.00041 | 342.00 | 9.26 | 2.69 | up | 0.01374 | 151.67 | 58.06 | 1.02 | down | 0.92769 |
| Lhx8 | 16875 | 3.29 | 0.00042 | 1176.83 | 30.71 | 1.76 | up | 0.04130 | 391.50 | 194.78 | 1.05 | down | 0.51890 |
| Hdc | 15186 | 19.81 | 0.00045 | 4800.67 | 1679.87 | 26.82 | up | 0.00544 | 263.33 | 81.13 | 5.11 | up | 0.00010 |
| Adamts9 | 101401 | 7.11 | 0.00048 | 615.50 | 79.07 | 63.27 | up | 0.00981 | 94.83 | 55.44 | 7.27 | up | 0.01071 |
| Bach2 | 12014 | 4.14 | 0.00051 | 341.83 | 43.69 | 3.42 | up | 0.02016 | 80.33 | 36.50 | 1.07 | down | 0.55491 |
| Osbpl9 | 100273 | 2.52 | 0.00054 | 11203.83 | 295.49 | 2.31 | up | 0.00317 | 4524.17 | 1475.45 | 1.02 | up | 0.85446 |
| Pbx1 | 18514 | 2.14 | 0.00055 | 466.17 | 133.08 | 2.40 | up | 0.01545 | 218.50 | 111.47 | 1.34 | up | 0.07408 |
| Itgb8 | 320910 | 3.75 | 0.00062 | 51.33 | 16.48 | 32.19 | up | 0.00067 | 15.83 | 4.54 | 7.08 | up | 0.02630 |
| Abca1 | 11303 | 2.40 | 0.00063 | 6842.67 | 732.83 | 4.17 | up | 0.00559 | 2692.33 | 888.65 | 1.59 | up | 0.15140 |
| Pvrl3 | 58998 | 2.63 | 0.00064 | 285.33 | 34.89 | 1.62 | up | 0.01520 | 125.33 | 33.71 | 1.37 | up | 0.30473 |
| Fam110c | 104943 | 5.19 | 0.00071 | 164.17 | 34.62 | 8.27 | up | 0.00401 | 35.50 | 22.95 | 3.04 | up | 0.04610 |
| Stfa3 | 20863 | 2.85 | 0.00078 | 274.33 | 51.92 | 6.07 | up | 0.02154 | 124.67 | 67.44 | 2.92 | up | 0.09083 |
| Mxd1 | 17119 | 2.45 | 0.00084 | 3184.67 | 412.43 | 4.04 | up | 0.00100 | 1292.50 | 394.68 | 2.43 | up | 0.02985 |
| Asb4 | 65255 | 5.61 | 0.00088 | 2212.17 | 475.56 | 8.53 | up | 0.00451 | 414.67 | 161.98 | 1.66 | up | 0.02469 |
| Sbno2 | 216161 | 2.45 | 0.00089 | 3867.67 | 260.49 | 2.42 | up | 0.01484 | 1829.83 | 958.30 | 1.15 | down | 0.49018 |
| Tmtc2 | 278279 | 4.46 | 0.00090 | 185.67 | 34.32 | 14.55 | up | 0.00374 | 41.17 | 16.00 | 2.81 | up | 0.04219 |
| Npy | 109648 | 6.74 | 0.00091 | 279.67 | 46.69 | 6.84 | up | 0.00871 | 47.33 | 33.08 | 2.24 | up | 0.03128 |
| Osbpl9 | 100273 | 2.27 | 0.00095 | 1086.00 | 43.35 | 2.13 | up | 0.00753 | 545.83 | 198.28 | 1.15 | up | 0.38849 |
| Edil3 | 13612 | 3.13 | 0.00104 | 109.33 | 15.04 | 2.17 | up | 0.00280 | 35.67 | 11.85 | 1.20 | up | 0.53844 |
| Mxi1 | 17859 | 3.09 | 0.00113 | 4299.50 | 510.79 | 4.69 | up | 0.01162 | 1463.67 | 444.57 | 1.86 | up | 0.08046 |
| Mospd1 | 70380 | 2.34 | 0.00113 | 962.50 | 50.01 | 1.66 | up | 0.02821 | 415.83 | 114.83 | 1.62 | down | 0.13794 |
| Hspa4l | 18415 | 2.16 | 0.00114 | 656.00 | 132.60 | 5.70 | up | 0.00081 | 361.83 | 138.52 | 3.09 | up | 0.00129 |
| Mex3b | 108797 | 2.26 | 0.00131 | 30.33 | 14.22 | 1.07 | up | 0.24072 | 10.17 | 6.37 | 2.16 | down | 0.02589 |
| Gpr35 | 64095 | 2.41 | 0.00132 | 685.33 | 75.66 | 36.41 | up | 0.00402 | 346.33 | 140.57 | 18.81 | up | 0.00123 |
| Crem | 12916 | 8.54 | 0.00145 | 7485.50 | 2380.71 | 13.23 | up | 0.01210 | 893.50 | 140.28 | 1.64 | up | 0.28833 |
| Fam107b | 66540 | 6.97 | 0.00149 | 51.67 | 1.61 | 6.17 | up | 0.00252 | 4.00 | 6.61 | 1.39 | up | 0.41827 |
| Pbx1 | 18514 | 2.42 | 0.00151 | 165.17 | 17.78 | 4.47 | up | 0.00302 | 70.50 | 42.67 | 2.66 | up | 0.00066 |
| Bend4 | 399584 | 3.01 | 0.00152 | 1273.83 | 33.34 | 2.79 | up | 0.01780 | 420.50 | 96.83 | 1.20 | down | 0.45089 |
| Atp10d | 231287 | 2.08 | 0.00158 | 707.50 | 35.57 | 2.37 | up | 0.01740 | 391.67 | 104.29 | 1.09 | up | 0.67754 |
| Mxd1 | 17119 | 2.43 | 0.00166 | 403.17 | 28.57 | 5.07 | up | 0.01446 | 186.17 | 79.27 | 2.57 | up | 0.00805 |
| Eml1 | 68519 | 4.49 | 0.00210 | 5144.50 | 764.32 | 13.62 | up | 0.00728 | 1120.67 | 270.10 | 5.77 | up | 0.00887 |
| Pdpn | 14726 | 3.33 | 0.00210 | 133.33 | 27.42 | 5.68 | up | 0.03266 | 45.83 | 42.25 | 3.99 | up | 0.04176 |
| Crem | 12916 | 9.34 | 0.00217 | 6383.67 | 2558.90 | 13.87 | up | 0.01420 | 737.67 | 41.05 | 1.53 | up | 0.34360 |
| Cacna1d | 12289 | 2.12 | 0.00219 | 64.67 | 24.17 | 2.06 | up | 0.01545 | 27.50 | 8.67 | 1.61 | up | 0.17051 |
| Hspa4l | 18415 | 2.17 | 0.00222 | 223.17 | 26.83 | 6.75 | up | 0.00548 | 107.83 | 50.10 | 3.79 | up | 0.00135 |
| Cpne8 | 66871 | 2.98 | 0.00229 | 504.17 | 96.05 | 3.14 | up | 0.00820 | 197.50 | 58.41 | 1.23 | up | 0.00251 |
| Ftsj2 | 68017 | 2.61 | 0.00234 | 105.83 | 9.52 | 1.59 | up | 0.01990 | 39.00 | 20.07 | 1.84 | down | 0.07009 |
| Gcc1 | 74375 | 2.75 | 0.00258 | 477.33 | 42.79 | 1.80 | up | 0.02694 | 183.00 | 70.93 | 1.69 | down | 0.15890 |
| Pitpnc1 | 71795 | 2.30 | 0.00295 | 245.17 | 31.27 | 5.53 | up | 0.01264 | 129.50 | 66.18 | 2.71 | up | 0.00978 |
| Thap2 | 66816 | 3.15 | 0.00300 | 262.33 | 33.65 | 4.91 | up | 0.00355 | 105.83 | 43.88 | 1.46 | up | 0.07644 |
| Setbp1 | 240427 | 3.42 | 0.00310 | 525.33 | 136.68 | 2.86 | up | 0.03255 | 168.00 | 99.52 | 1.45 | up | 0.26976 |
| Crem | 12916 | 8.12 | 0.00311 | 6441.33 | 2100.86 | 12.21 | up | 0.01419 | 694.83 | 86.68 | 1.66 | up | 0.21733 |
| Mafb | 16658 | 2.50 | 0.00318 | 2995.67 | 340.51 | 2.11 | down | 0.05465 | 1283.00 | 586.68 | 6.01 | down | 0.01769 |
| Pla2g4a | 18783 | 2.96 | 0.00325 | 1203.33 | 178.69 | 5.64 | up | 0.00219 | 462.67 | 148.42 | 12.24 | up | 0.00152 |
| Rab44 | 442827 | 9.01 | 0.00326 | 125.17 | 46.78 | 5.06 | up | 0.01389 | 15.33 | 6.43 | 1.33 | up | 0.54512 |
| Crem | 12916 | 7.67 | 0.00342 | 2919.50 | 1229.25 | 10.29 | up | 0.02955 | 390.83 | 100.00 | 1.61 | up | 0.24806 |
| Asb4 | 65255 | 5.36 | 0.00344 | 86.83 | 19.91 | 6.48 | up | 0.01047 | 15.17 | 16.65 | 1.90 | up | 0.05453 |
| Odc1 | 18263 | 2.01 | 0.00355 | 411.00 | 38.97 | 7.92 | up | 0.00261 | 254.00 | 72.27 | 3.59 | up | 0.01521 |
| Fam107b | 66540 | 5.71 | 0.00362 | 2238.67 | 293.82 | 4.90 | up | 0.01509 | 402.83 | 58.21 | 1.25 | up | 0.25197 |
| Kctd12b | 207474 | 2.50 | 0.00363 | 116.00 | 17.02 | 3.64 | down | 0.01764 | 46.00 | 16.48 | 4.40 | down | 0.07332 |
| Ddah2 | 51793 | 2.41 | 0.00394 | 763.00 | 92.22 | 3.45 | up | 0.00006 | 333.00 | 76.99 | 1.32 | up | 0.28368 |
| Kctd4 | 67516 | 4.41 | 0.00413 | 100.00 | 11.53 | 8.93 | up | 0.02658 | 20.17 | 4.31 | 2.41 | up | 0.18509 |
| Ereg | 13874 | 3.67 | 0.00418 | 63.33 | 17.01 | 33.16 | up | 0.02463 | 23.50 | 5.27 | 9.11 | up | 0.01251 |
| Bcl6 | 12053 | 2.57 | 0.00419 | 3128.00 | 282.16 | 1.86 | down | 0.02540 | 1350.50 | 188.17 | 4.92 | down | 0.01826 |
| Sipa1l2 | 244668 | 2.02 | 0.00424 | 799.67 | 98.32 | 3.03 | up | 0.03989 | 450.50 | 161.98 | 2.04 | up | 0.02416 |
| Fam107b | 66540 | 4.95 | 0.00427 | 205.50 | 21.40 | 7.39 | up | 0.00402 | 39.33 | 6.53 | 1.89 | up | 0.08332 |
| Crem | 12916 | 7.08 | 0.00431 | 2477.17 | 1070.80 | 10.15 | up | 0.02159 | 344.83 | 62.50 | 1.50 | up | 0.25215 |
| Edil3 | 13612 | 2.87 | 0.00442 | 455.33 | 47.28 | 1.55 | up | 0.02659 | 200.17 | 74.88 | 1.03 | down | 0.82724 |
| Fosl2 | 14284 | 2.38 | 0.00445 | 1500.33 | 55.96 | 3.50 | up | 0.01399 | 799.67 | 268.01 | 1.32 | up | 0.49660 |
| Itga9 | 104099 | 2.95 | 0.00452 | 4514.67 | 574.52 | 3.77 | up | 0.00408 | 1418.83 | 383.84 | 1.66 | up | 0.00593 |
| Itgav | 16410 | 2.28 | 0.00454 | 166.83 | 42.26 | 6.63 | up | 0.00655 | 81.67 | 35.64 | 5.86 | up | 0.00498 |
| Cnnm2 | 94219 | 3.02 | 0.00461 | 234.83 | 23.68 | 2.06 | up | 0.00616 | 78.00 | 28.58 | 1.20 | down | 0.21359 |
| Apbb3 | 225372 | 2.76 | 0.00467 | 2207.33 | 458.94 | 4.13 | up | 0.00908 | 864.33 | 327.09 | 1.53 | up | 0.03350 |
| Zc3hav1l | 320578 | 3.31 | 0.00471 | 88.33 | 32.30 | 5.56 | up | 0.02119 | 28.00 | 22.15 | 2.37 | up | 0.01431 |
| Pex5 | 19305 | 2.02 | 0.00486 | 1082.33 | 134.31 | 2.79 | up | 0.01896 | 510.50 | 151.57 | 1.42 | up | 0.07795 |
| Stat3 | 20848 | 2.57 | 0.00501 | 48.83 | 5.39 | 4.02 | up | 0.00247 | 15.33 | 15.01 | 1.47 | up | 0.05033 |
| Nt5e | 23959 | 5.46 | 0.00532 | 1020.67 | 169.91 | 1.64 | up | 0.04542 | 206.67 | 53.52 | 1.28 | down | 0.33273 |
| Itga9 | 102527 | 2.72 | 0.00537 | 4102.33 | 1011.65 | 3.60 | up | 0.00843 | 1603.67 | 628.94 | 1.56 | up | 0.03562 |
| Hecw2 | 329152 | 3.36 | 0.00540 | 51.00 | 14.40 | 5.51 | up | 0.01719 | 12.83 | 7.65 | 1.58 | up | 0.55290 |
| Edil3 | 13612 | 13.44 | 0.00592 | 67.00 | 39.47 | 13.72 | up | 0.04603 | 5.17 | 3.62 | 1.38 | up | 0.57269 |
| Trem1 | 58217 | 6.69 | 0.00619 | 676.50 | 296.25 | 11.03 | up | 0.01129 | 107.00 | 48.16 | 2.74 | up | 0.02547 |
| Ramp3 | 56089 | 3.39 | 0.00620 | 3473.00 | 589.00 | 12.53 | up | 0.02042 | 1036.00 | 270.86 | 5.84 | up | 0.02476 |
| Mat2b | 108645 | 4.96 | 0.00635 | 1697.50 | 503.46 | 2.80 | up | 0.03923 | 345.83 | 42.62 | 1.70 | down | 0.10171 |
| Gja1 | 14609 | 5.19 | 0.00638 | 90.33 | 23.74 | 50.69 | up | 0.00373 | 12.17 | 18.95 | 3.89 | up | 0.15451 |
| Lepr | 16847 | 10.82 | 0.00648 | 119.67 | 37.10 | 2.19 | up | 0.03311 | 12.67 | 9.70 | 1.21 | up | 0.42990 |
| Ccng1 | 12450 | 2.62 | 0.00658 | 833.67 | 61.37 | 1.19 | up | 0.20065 | 316.17 | 51.38 | 2.40 | down | 0.01169 |
| Oscp1 | 230751 | 5.13 | 0.00660 | 216.00 | 72.92 | 1.31 | up | 0.16836 | 38.33 | 13.16 | 2.29 | down | 0.04109 |
| Kctd4 | 67516 | 3.10 | 0.00667 | 187.67 | 33.06 | 9.84 | up | 0.03648 | 61.33 | 29.54 | 4.89 | up | 0.06553 |
| Rnf150 | 77483 | 5.94 | 0.00687 | 205.67 | 42.50 | 1.77 | up | 0.11695 | 36.33 | 15.83 | 2.25 | down | 0.00597 |
| Nus1 | 52014 | 2.23 | 0.00696 | 716.17 | 57.00 | 2.00 | up | 0.02163 | 366.83 | 76.00 | 1.20 | down | 0.45892 |
| Lhx8 | 16875 | 2.97 | 0.00727 | 438.00 | 60.36 | 1.75 | up | 0.02377 | 157.83 | 115.14 | 1.11 | up | 0.23569 |
| Rnf125 | 67664 | 5.56 | 0.00785 | 129.33 | 9.67 | 3.40 | up | 0.00768 | 35.67 | 39.06 | 1.29 | down | 0.30965 |
| Tnfrsf9 | 21942 | 2.14 | 0.00791 | 108.33 | 34.02 | 8.21 | up | 0.01281 | 56.67 | 38.94 | 5.70 | up | 0.07722 |
| Aph1c | 68318 | 2.07 | 0.00809 | 108.00 | 21.93 | 1.25 | up | 0.28371 | 58.33 | 30.24 | 1.81 | down | 0.00121 |
| Nr4a2 | 18227 | 7.15 | 0.00826 | 8312.67 | 4057.49 | 80.95 | up | 0.00295 | 1056.67 | 350.95 | 9.97 | up | 0.01498 |
| Lrrc16a | 68732 | 2.06 | 0.00841 | 136.17 | 22.89 | 3.41 | up | 0.00650 | 63.83 | 16.31 | 2.48 | up | 0.00921 |
| Kcna3 | 16491 | 2.50 | 0.00849 | 45.00 | 10.04 | 3.11 | up | 0.01794 | 20.83 | 14.02 | 1.49 | up | 0.06724 |
| Pla2g4a | 18783 | 2.94 | 0.00867 | 2417.00 | 589.44 | 5.63 | up | 0.00718 | 915.17 | 156.34 | 10.86 | up | 0.00010 |
| Lpar6 | 67168 | 2.15 | 0.00867 | 190.83 | 19.53 | 2.69 | down | 0.12398 | 97.00 | 57.42 | 7.03 | down | 0.03175 |
| Tspan13 | 66109 | 2.94 | 0.00871 | 1414.50 | 160.06 | 1.04 | up | 0.72683 | 496.67 | 124.96 | 2.42 | down | 0.00222 |
| Ucp1 | 22227 | 6.88 | 0.00873 | 66.33 | 20.13 | 4.34 | up | 0.02539 | 6.67 | 11.06 | 1.06 | down | 0.92665 |
| Trem1 | 58217 | 10.32 | 0.00888 | 1284.33 | 642.10 | 20.85 | up | 0.00539 | 129.67 | 58.01 | 5.90 | up | 0.01055 |
| Mmp13 | 17386 | 2.58 | 0.00902 | 3499.67 | 1047.79 | 6.99 | up | 0.00386 | 1352.67 | 377.02 | 4.49 | up | 0.01078 |
| Mat2b | 108645 | 4.53 | 0.00902 | 6013.00 | 2357.00 | 2.75 | up | 0.04511 | 1332.17 | 531.35 | 1.79 | down | 0.09043 |
| Plagl1 | 22634 | 2.61 | 0.00981 | 315.33 | 30.92 | 7.50 | up | 0.02630 | 127.67 | 63.58 | 4.79 | up | 0.02162 |
| Lrrfip1 | 16978 | 2.61 | 0.00984 | 402.67 | 68.01 | 1.41 | up | 0.01896 | 163.00 | 63.50 | 1.55 | down | 0.01731 |
| Ppm1e | 320472 | 2.17 | 0.01044 | 34.50 | 1.32 | 1.70 | down | 0.00281 | 14.33 | 7.57 | 2.43 | down | 0.06499 |
| Saa1 | 20208 | 2.82 | 0.01067 | 396.17 | 85.98 | 2.94 | up | 0.03364 | 167.83 | 116.77 | 1.02 | up | 0.88753 |
| Fam20a | 208659 | 2.86 | 0.01067 | 3498.83 | 592.48 | 2.64 | up | 0.04467 | 1253.17 | 393.78 | 1.33 | up | 0.24572 |
| Cpne8 | 66871 | 2.81 | 0.01107 | 2077.00 | 293.74 | 3.74 | up | 0.00013 | 841.17 | 77.03 | 1.22 | up | 0.05614 |
| Man1a | 17155 | 2.38 | 0.01110 | 9290.50 | 1017.66 | 3.41 | up | 0.01835 | 4382.50 | 775.69 | 1.64 | up | 0.05441 |
| Ccno | 218630 | 6.02 | 0.01134 | 2432.50 | 1060.73 | 10.77 | up | 0.00178 | 355.67 | 36.30 | 2.33 | up | 0.14907 |
| Il10 | 16153 | 13.06 | 0.01156 | 2118.67 | 1326.38 | 78.03 | up | 0.02867 | 150.83 | 46.11 | 7.58 | up | 0.01463 |
| Antxr2 | 71914 | 3.80 | 0.01165 | 2261.83 | 576.49 | 1.62 | up | 0.21731 | 623.00 | 78.00 | 1.53 | down | 0.03964 |
| Tnfaip6 | 21930 | 2.59 | 0.01175 | 64.67 | 1.89 | 14.19 | up | 0.03943 | 27.17 | 23.35 | 6.12 | up | 0.04223 |
| Inhbb | 16324 | 4.49 | 0.01244 | 75.00 | 23.97 | 7.87 | up | 0.01325 | 21.00 | 26.56 | 1.24 | up | 0.54779 |
| Thbd | 21824 | 6.66 | 0.01265 | 730.33 | 362.68 | 1.77 | down | 0.29053 | 94.83 | 41.59 | 8.24 | down | 0.01558 |
| Eaf2 | 106389 | 2.13 | 0.01303 | 50.67 | 10.02 | 3.70 | up | 0.03508 | 28.33 | 5.13 | 1.68 | up | 0.04957 |
| Flrt3 | 71436 | 2.16 | 0.01317 | 1301.50 | 173.59 | 32.42 | up | 0.00518 | 579.50 | 124.62 | 15.19 | up | 0.01100 |
| Calca | 12310 | 2.57 | 0.01334 | 215.50 | 37.25 | 4.50 | up | 0.05062 | 115.00 | 79.02 | 1.67 | up | 0.37356 |
| Frat1 | 14296 | 2.36 | 0.01339 | 97.17 | 39.45 | 2.60 | down | 0.03683 | 35.17 | 19.85 | 5.29 | down | 0.02497 |
| Jag2 | 16450 | 5.73 | 0.01375 | 40.83 | 12.45 | 16.42 | up | 0.00364 | 5.17 | 3.40 | 3.71 | up | 0.01184 |
| Mrpl52 | 68836 | 3.63 | 0.01422 | 1482.67 | 441.28 | 7.50 | up | 0.01028 | 394.50 | 106.63 | 2.07 | up | 0.08785 |
| Socs3 | 12702 | 2.51 | 0.01440 | 71520.60 | 19416.89 | 16.51 | up | 0.00343 | 31123.90 | 10271.08 | 6.51 | up | 0.00099 |
| Zfp36l1 | 12192 | 2.23 | 0.01454 | 3851.67 | 243.43 | 4.20 | up | 0.01087 | 1583.33 | 466.75 | 1.46 | up | 0.22168 |
| Havcr2 | 171285 | 7.39 | 0.01472 | 70.67 | 28.87 | 4.48 | up | 0.02876 | 11.00 | 5.20 | 1.41 | up | 0.02424 |
| Ttc39b | 69863 | 2.08 | 0.01478 | 627.83 | 44.81 | 6.71 | up | 0.01128 | 317.50 | 73.25 | 3.79 | up | 0.01226 |
| Fam110c | 104943 | 6.54 | 0.01487 | 97.17 | 54.36 | 13.87 | up | 0.01221 | 14.50 | 20.48 | 5.41 | up | 0.00785 |
| Saa2 | 20209 | 2.45 | 0.01505 | 704.67 | 159.95 | 2.89 | up | 0.03174 | 361.33 | 239.08 | 1.05 | up | 0.76555 |
| Runx2 | 12393 | 2.87 | 0.01559 | 80.50 | 12.44 | 3.68 | up | 0.00397 | 27.83 | 9.12 | 1.15 | up | 0.47087 |
| Itgav | 16410 | 2.52 | 0.01585 | 166.17 | 8.69 | 11.01 | up | 0.00979 | 60.50 | 21.22 | 5.64 | up | 0.01579 |
| Zfp36l1 | 12192 | 2.53 | 0.01588 | 4101.50 | 471.25 | 4.28 | up | 0.01389 | 1675.83 | 431.77 | 1.31 | up | 0.09354 |
| Bcl2l11 | 12125 | 2.45 | 0.01590 | 367.33 | 114.81 | 8.73 | up | 0.01115 | 167.83 | 50.46 | 4.90 | up | 0.00764 |
| Il1f6 | 54448 | 2.60 | 0.01599 | 229.17 | 53.58 | 47.20 | up | 0.02378 | 96.50 | 64.12 | 15.13 | up | 0.00806 |
| Gcnt2 | 14538 | 3.95 | 0.01617 | 43.67 | 14.01 | 7.07 | up | 0.01808 | 10.67 | 6.81 | 4.29 | up | 0.09916 |
| Flrt3 | 71436 | 2.43 | 0.01683 | 5553.83 | 906.89 | 33.20 | up | 0.00252 | 2729.67 | 188.90 | 17.93 | up | 0.00607 |
| Cd80 | 12519 | 3.06 | 0.01716 | 532.50 | 143.32 | 5.32 | up | 0.01806 | 177.17 | 19.64 | 1.95 | up | 0.07323 |
| Slc38a1 | 105727 | 2.11 | 0.01717 | 420.67 | 88.73 | 3.96 | up | 0.00339 | 229.67 | 66.04 | 1.74 | up | 0.00646 |
| Dusp14 | 56405 | 3.46 | 0.01751 | 146.83 | 75.10 | 8.60 | up | 0.02807 | 40.50 | 23.07 | 2.17 | up | 0.09120 |
| Arid3b | 56380 | 2.03 | 0.01758 | 293.33 | 116.62 | 1.20 | up | 0.44000 | 130.83 | 61.94 | 1.61 | down | 0.04856 |
| Dab2 | 13132 | 2.60 | 0.01771 | 5221.17 | 784.31 | 3.43 | up | 0.00076 | 2155.83 | 367.51 | 1.76 | up | 0.10474 |
| Plekhg3 | 263406 | 2.10 | 0.01772 | 680.00 | 94.95 | 1.91 | down | 0.02022 | 334.50 | 116.23 | 3.79 | down | 0.00489 |
| Runx2 | 12393 | 3.00 | 0.01806 | 38.00 | 8.05 | 2.99 | up | 0.03422 | 17.50 | 13.81 | 1.01 | down | 0.95042 |
| Zfp449 | 78619 | 2.67 | 0.01810 | 26.00 | 20.79 | 1.21 | up | 0.53464 | 2.83 | 1.04 | 3.28 | down | 0.02549 |
| Gprc5a | 232431 | 3.02 | 0.01819 | 247.33 | 45.61 | 6.05 | up | 0.03482 | 73.00 | 8.79 | 2.38 | up | 0.17275 |
| Rgs2 | 19735 | 3.96 | 0.01847 | 14864.00 | 3728.42 | 1.17 | up | 0.43249 | 3773.00 | 1203.17 | 3.17 | down | 0.03218 |
| Rnd3 | 74194 | 2.05 | 0.01864 | 41.83 | 4.54 | 1.35 | down | 0.42053 | 23.83 | 11.51 | 2.44 | down | 0.01170 |
| Nudt4 | 71207 | 2.18 | 0.01865 | 179.17 | 16.25 | 2.37 | up | 0.03620 | 70.83 | 27.61 | 1.09 | up | 0.10587 |
| Mmd | 67468 | 4.50 | 0.01867 | 320.00 | 128.21 | 2.80 | up | 0.02499 | 78.33 | 26.08 | 1.28 | down | 0.34298 |
| Mpzl1 | 68481 | 2.97 | 0.01888 | 315.33 | 124.94 | 4.14 | up | 0.04149 | 116.83 | 89.64 | 1.63 | up | 0.15632 |
| Mmp3 | 17392 | 4.80 | 0.01910 | 365.33 | 191.06 | 23.62 | up | 0.01001 | 70.67 | 22.25 | 6.31 | up | 0.01145 |
| Pitpnc1 | 71795 | 2.22 | 0.01923 | 613.50 | 151.35 | 3.66 | up | 0.01582 | 264.00 | 100.34 | 1.99 | up | 0.04196 |
| Fst | 14313 | 4.89 | 0.01977 | 338.17 | 140.65 | 28.01 | up | 0.00730 | 68.67 | 44.47 | 5.76 | up | 0.13125 |
| Tmem144 | 70652 | 2.47 | 0.02000 | 88.83 | 9.46 | 4.08 | up | 0.01587 | 39.83 | 14.63 | 1.33 | up | 0.06674 |
| Lifr | 16880 | 2.22 | 0.02125 | 109.33 | 24.01 | 3.08 | up | 0.01287 | 41.50 | 12.82 | 1.46 | up | 0.32577 |
| Fam117b | 72750 | 2.30 | 0.02154 | 168.00 | 26.01 | 2.48 | up | 0.01489 | 71.50 | 29.95 | 1.24 | down | 0.32398 |
| Sema6d | 214968 | 2.11 | 0.02219 | 36.00 | 8.05 | 3.75 | up | 0.06072 | 17.17 | 19.11 | 1.23 | up | 0.35389 |
| Nudt4 | 71207 | 2.15 | 0.02273 | 520.50 | 89.45 | 1.49 | up | 0.06721 | 260.00 | 32.45 | 1.53 | down | 0.02843 |
| Plcl1 | 227120 | 3.30 | 0.02281 | 58.17 | 13.00 | 5.62 | up | 0.00479 | 17.67 | 5.13 | 2.27 | up | 0.02475 |
| Hes1 | 15205 | 2.51 | 0.02320 | 153.83 | 36.53 | 8.67 | up | 0.03209 | 59.00 | 21.52 | 4.48 | up | 0.04432 |
| Csf3 | 12985 | 4.74 | 0.02326 | 10887.83 | 5124.02 | 643.38 | up | 0.00359 | 2165.83 | 173.19 | 144.04 | up | 0.00653 |
| Ptger2 | 19217 | 2.44 | 0.02330 | 177.17 | 65.21 | 1.10 | up | 0.74019 | 71.67 | 34.06 | 2.48 | down | 0.03026 |
| Uck2 | 80914 | 4.35 | 0.02383 | 534.33 | 189.15 | 9.80 | up | 0.00480 | 122.67 | 43.97 | 2.72 | up | 0.15994 |
| Sipa1l1 | 217692 | 2.74 | 0.02387 | 5611.33 | 1828.93 | 4.13 | up | 0.01357 | 2195.67 | 378.96 | 1.77 | up | 0.07177 |
| Lepr | 16847 | 12.01 | 0.02415 | 117.00 | 59.35 | 2.61 | up | 0.04175 | 12.50 | 15.40 | 1.04 | up | 0.95236 |
| Maob | 109731 | 3.39 | 0.02457 | 50.17 | 21.70 | 1.62 | up | 0.03510 | 11.33 | 3.75 | 1.46 | down | 0.23715 |
| Eml1 | 68519 | 3.38 | 0.02475 | 312.17 | 69.72 | 25.35 | up | 0.00737 | 89.83 | 24.22 | 13.59 | up | 0.01324 |
| Phactr1 | 218194 | 2.04 | 0.02488 | 118.33 | 23.91 | 1.97 | up | 0.00275 | 53.00 | 12.82 | 1.04 | down | 0.67224 |
| Gcsh | 68133 | 2.70 | 0.02522 | 392.33 | 59.37 | 1.97 | up | 0.01632 | 148.33 | 5.13 | 1.37 | down | 0.29291 |
| Tshz3 | 243931 | 2.21 | 0.02553 | 129.67 | 28.15 | 2.60 | up | 0.00211 | 54.00 | 21.17 | 1.18 | up | 0.38659 |
| Sipa1l1 | 217692 | 2.48 | 0.02619 | 153.17 | 49.08 | 2.87 | up | 0.01610 | 55.00 | 19.97 | 1.21 | up | 0.22300 |
| Pitpnc1 | 71795 | 2.40 | 0.02641 | 846.83 | 217.42 | 3.81 | up | 0.02500 | 377.83 | 187.08 | 1.90 | up | 0.00024 |
| Mospd4 | 72076 | 2.29 | 0.02677 | 143.17 | 46.41 | 2.20 | up | 0.04720 | 77.17 | 61.83 | 1.34 | up | 0.31725 |
| Capzb | 12345 | 2.20 | 0.02763 | 135.50 | 57.94 | 3.97 | up | 0.00922 | 73.50 | 50.57 | 3.11 | up | 0.19065 |
| Cytip | 227929 | 4.96 | 0.02844 | 2511.83 | 1234.43 | 4.56 | up | 0.01481 | 472.33 | 78.03 | 1.04 | down | 0.92163 |
| Cep120 | 225523 | 2.06 | 0.02845 | 96.83 | 19.53 | 1.14 | up | 0.26876 | 44.83 | 20.25 | 1.92 | down | 0.03880 |
| Entpd1 | 12495 | 2.69 | 0.02896 | 135.50 | 40.44 | 2.55 | up | 0.04432 | 51.17 | 12.39 | 1.09 | down | 0.17375 |
| Trem3 | 58218 | 2.33 | 0.02932 | 173.83 | 32.63 | 3.06 | up | 0.01122 | 68.17 | 18.35 | 1.69 | up | 0.07058 |
| Spag9 | 70834 | 2.14 | 0.02955 | 423.00 | 35.07 | 1.85 | up | 0.01678 | 222.83 | 85.31 | 1.02 | down | 0.93231 |
| Atp10d | 231287 | 2.33 | 0.02958 | 368.67 | 39.31 | 2.09 | up | 0.02249 | 162.67 | 49.65 | 1.11 | down | 0.19361 |
| Dedd2 | 67379 | 2.24 | 0.03023 | 1289.67 | 339.15 | 1.49 | up | 0.16241 | 630.17 | 111.42 | 1.46 | down | 0.01356 |
| Zfp36 | 22695 | 2.04 | 0.03104 | 1127.83 | 275.07 | 10.11 | up | 0.00615 | 564.33 | 136.68 | 4.18 | up | 0.00911 |
| Gata2 | 14461 | 4.60 | 0.03125 | 108.67 | 77.00 | 1.45 | up | 0.36545 | 19.33 | 1.53 | 2.96 | up | 0.05016 |
| Trim17 | 56631 | 2.87 | 0.03214 | 61.00 | 28.16 | 2.42 | up | 0.03633 | 18.00 | 6.95 | 1.58 | down | 0.51197 |
| Uck2 | 80914 | 4.59 | 0.03216 | 670.50 | 318.73 | 13.65 | up | 0.00127 | 160.67 | 31.50 | 3.48 | up | 0.09389 |
| Rap1gap2 | 380711 | 2.23 | 0.03231 | 317.00 | 71.23 | 1.79 | up | 0.02095 | 157.33 | 35.83 | 1.06 | down | 0.76288 |
| Nus1 | 52014 | 2.00 | 0.03247 | 3362.33 | 416.19 | 3.09 | up | 0.01684 | 1486.83 | 169.95 | 1.82 | up | 0.03765 |
| Chst11 | 58250 | 2.46 | 0.03287 | 183.67 | 64.84 | 10.44 | up | 0.02179 | 82.67 | 31.81 | 2.62 | up | 0.14116 |
| Lpar6 | 67168 | 2.10 | 0.03289 | 939.67 | 227.42 | 2.61 | down | 0.09624 | 475.33 | 152.68 | 6.27 | down | 0.02848 |
| Slc38a1 | 105727 | 2.10 | 0.03370 | 4292.00 | 453.92 | 3.70 | up | 0.00775 | 2171.67 | 796.94 | 2.05 | up | 0.11173 |
| Trim25 | 217069 | 2.38 | 0.03448 | 422.83 | 96.38 | 6.32 | up | 0.04207 | 174.83 | 39.92 | 2.81 | up | 0.03179 |
| Cadm1 | 54725 | 2.07 | 0.03483 | 1416.00 | 59.11 | 1.85 | down | 0.00774 | 762.33 | 345.27 | 2.11 | down | 0.02256 |
| Hdac5 | 15184 | 2.60 | 0.03565 | 6105.83 | 1631.56 | 1.21 | up | 0.50729 | 2541.83 | 461.92 | 1.90 | down | 0.02335 |
| Chd7 | 320790 | 2.85 | 0.03588 | 2868.83 | 1000.11 | 2.77 | up | 0.03593 | 1068.33 | 272.52 | 1.02 | down | 0.92508 |
| Tnrc6b | 213988 | 2.48 | 0.03634 | 119.17 | 0.76 | 1.80 | down | 0.02496 | 51.33 | 11.15 | 4.26 | down | 0.04952 |
| Dusp14 | 56405 | 4.64 | 0.03644 | 263.67 | 116.90 | 138.02 | up | 0.00732 | 53.67 | 30.07 | 19.00 | up | 0.04635 |
| Tle1 | 21885 | 2.76 | 0.03658 | 477.50 | 153.99 | 2.03 | up | 0.04750 | 179.33 | 32.37 | 1.10 | up | 0.19122 |
| Tmem144 | 70652 | 2.36 | 0.03771 | 122.83 | 14.37 | 5.78 | up | 0.01327 | 57.00 | 14.18 | 2.52 | up | 0.05058 |
| Foxc1 | 17300 | 3.13 | 0.03872 | 42.17 | 11.45 | 4.36 | up | 0.00859 | 11.50 | 9.04 | 1.78 | up | 0.38518 |
| Prox1 | 19130 | 2.56 | 0.03916 | 80.00 | 16.09 | 3.88 | up | 0.00839 | 31.67 | 37.86 | 1.26 | up | 0.62397 |
| Areg | 11839 | 9.82 | 0.03994 | 1061.83 | 889.33 | 381.19 | up | 0.00596 | 91.83 | 30.46 | 48.70 | up | 0.00496 |
| S1pr1 | 13609 | 2.15 | 0.04033 | 427.83 | 101.49 | 3.78 | down | 0.00317 | 204.00 | 39.61 | 6.97 | down | 0.01084 |
| Gls2 | 216456 | 5.71 | 0.04064 | 44.83 | 19.90 | 7.42 | up | 0.00731 | 9.83 | 22.99 | 1.12 | down | 0.19430 |
| Epha2 | 13836 | 2.61 | 0.04072 | 668.67 | 233.91 | 14.46 | up | 0.03426 | 255.00 | 75.35 | 6.59 | up | 0.06372 |
| Smox | 228608 | 2.54 | 0.04210 | 356.00 | 99.16 | 4.98 | up | 0.01666 | 132.83 | 45.84 | 3.42 | up | 0.01186 |
| Fam184b | 58227 | 2.92 | 0.04277 | 38.67 | 37.27 | 3.55 | up | 0.01185 | 10.50 | 4.77 | 2.04 | down | 0.22034 |
| Lrrfip1 | 16978 | 3.20 | 0.04338 | 5281.50 | 924.91 | 3.69 | up | 0.00287 | 1812.50 | 630.51 | 1.40 | up | 0.40372 |
| Cbln3 | 56410 | 2.26 | 0.04417 | 87.50 | 31.32 | 4.11 | up | 0.01107 | 43.33 | 33.01 | 1.64 | up | 0.12052 |
| Bmp6 | 12161 | 3.40 | 0.04551 | 104.33 | 79.39 | 4.49 | up | 0.00310 | 24.17 | 21.66 | 1.64 | up | 0.03225 |
| Phactr1 | 218194 | 2.09 | 0.04587 | 474.33 | 125.86 | 1.74 | up | 0.02762 | 243.83 | 86.78 | 1.01 | up | 0.97768 |
| Ctla2b | 13025 | 2.56 | 0.04809 | 4769.67 | 2050.53 | 1.98 | up | 0.03373 | 1952.00 | 231.78 | 1.12 | down | 0.59446 |
| Stat3 | 20848 | 2.57 | 0.00501 | 48.83 | 5.39 | 4.02 | up | 0.00247 | 15.33 | 15.01 | 1.47 | up | 0.05033 |
| Mt1 | 17748 | 2.24 | 0.01887 | 1015.17 | 329.00 | 8.64 | up | 0.01157 | 512.67 | 232.62 | 4.54 | up | 0.00323 |
| Ralgapa2 | 241694 | 6.43 | 0.02326 | 34.83 | 40.91 | 3.10 | up | 0.09659 | 1.33 | 5.03 | 6.06 | down | 0.15757 |
| Cuedc1 | 103841 | 2.00 | 0.00487 | 1179.33 | 170.81 | 7.31 | up | 0.01406 | 668.17 | 326.76 | 3.64 | up | 0.06841 |
| Arl4a | 11861 | 2.00 | 0.04492 | 1269.33 | 178.61 | 7.17 | up | 0.01145 | 574.17 | 85.92 | 4.05 | up | 0.02788 |
| Cd14 | 12475 | 2.00 | 0.01654 | 190008.17 | 17670.39 | 3.59 | up | 0.02098 | 101534.80 | 19232.10 | 2.17 | up | 0.02369 |
| Niacr1 | 80885 | 2.00 | 0.04394 | 25883.00 | 2490.77 | 10.92 | up | 0.02347 | 13340.67 | 3797.86 | 5.14 | up | 0.04237 |
| Rai14 | 75646 | 2.00 | 0.02227 | 118.83 | 12.27 | 8.74 | up | 0.03304 | 73.83 | 38.03 | 6.58 | up | 0.00269 |
| Parp8 | 52552 | 2.00 | 0.03479 | 734.67 | 159.15 | 2.36 | up | 0.01964 | 359.33 | 83.79 | 1.06 | up | 0.72246 |
| Spnb2 | 20742 | 2.00 | 0.01104 | 55.17 | 8.81 | 1.17 | up | 0.09107 | 31.33 | 12.25 | 2.02 | down | 0.01312 |
| Man1a | 17155 | 2.00 | 0.00184 | 301.33 | 81.73 | 2.67 | up | 0.00325 | 155.00 | 84.04 | 1.67 | up | 0.07203 |
| Hspa4l | 18415 | 2.00 | 0.00249 | 3546.50 | 304.59 | 5.78 | up | 0.00210 | 1817.33 | 528.19 | 3.97 | up | 0.00146 |
| Stat3 | 20848 | 2.00 | 0.02637 | 9585.50 | 919.06 | 3.88 | up | 0.00685 | 4702.83 | 656.17 | 1.75 | up | 0.13248 |
| Stat3 | 20848 | 2.00 | 0.02637 | 9930.50 | 837.49 | 3.88 | up | 0.00685 | 4872.50 | 713.21 | 1.75 | up | 0.13248 |
| Stat3 | 20848 | 2.00 | 0.02637 | 9952.50 | 971.39 | 3.88 | up | 0.00685 | 4858.33 | 925.55 | 1.75 | up | 0.13248 |
| Stat3 | 20848 | 2.00 | 0.02637 | 11467.83 | 1789.86 | 3.88 | up | 0.00685 | 5902.33 | 1212.15 | 1.75 | up | 0.13248 |
| Stat3 | 20848 | 2.00 | 0.02637 | 10612.33 | 1598.60 | 3.88 | up | 0.00685 | 5434.67 | 1066.86 | 1.75 | up | 0.13248 |
| Stat3 | 20848 | 2.00 | 0.02637 | 10684.50 | 1502.05 | 3.88 | up | 0.00685 | 5589.00 | 1168.03 | 1.75 | up | 0.13248 |
| Stat3 | 20848 | 2.00 | 0.02637 | 9186.33 | 2185.10 | 3.88 | up | 0.00685 | 4785.00 | 1088.71 | 1.75 | up | 0.13248 |
| Stat3 | 20848 | 2.00 | 0.02637 | 8303.83 | 1985.30 | 3.88 | up | 0.00685 | 4415.83 | 855.03 | 1.75 | up | 0.13248 |
| Stat3 | 20848 | 2.00 | 0.02637 | 7974.50 | 738.33 | 3.88 | up | 0.00685 | 3856.17 | 668.96 | 1.75 | up | 0.13248 |
| Stat3 | 20848 | 2.00 | 0.02637 | 7827.00 | 1379.08 | 3.88 | up | 0.00685 | 4032.33 | 825.28 | 1.75 | up | 0.13248 |
| Serinc5 | 218442 | 2.00 | 0.00117 | 515.67 | 56.85 | 1.04 | down | 0.30226 | 268.17 | 77.55 | 2.20 | down | 0.02315 |
| Cacna1d | 12289 | 2.00 | 0.02399 | 1481.67 | 260.36 | 2.47 | up | 0.00002 | 759.50 | 138.78 | 1.75 | up | 0.07741 |
